# High-confidence structural annotation of metabolites absent from spectral libraries

**DOI:** 10.1038/s41587-021-01045-9

**Published:** 2021-10-14

**Authors:** Martin A. Hoffmann, Louis-Félix Nothias, Marcus Ludwig, Markus Fleischauer, Emily C. Gentry, Michael Witting, Pieter C. Dorrestein, Kai Dührkop, Sebastian Böcker

**Affiliations:** 1 Chair for Bioinformatics, Faculty of Mathematics and Computer Science, Friedrich Schiller University Jena, Jena, Germany; 2 International Max Planck Research School ‘Exploration of Ecological Interactions with Molecular and Chemical Techniques’, Max Planck Institute for Chemical Ecology, Jena, Germany; 3 Collaborative Mass Spectrometry Innovation Center, Skaggs School of Pharmacy and Pharmaceutical Sciences, University of California, San Diego, San Diego, CA USA; 4 Metabolomics and Proteomics Core, Helmholtz Zentrum München, Neuherberg, Germany; 5 Chair of Analytical Food Chemistry, TUM School of Life Sciences, Technical University of Munich, Freising-Weihenstephan, Germany; 6 Departments of Pharmacology and Pediatrics, University of California, San Diego, San Diego, CA USA; 7 Present Address: School of Pharmaceutical Sciences, University of Geneva, Geneva, Switzerland

**Keywords:** Molecular biology, Data processing

## Abstract

Untargeted metabolomics experiments rely on spectral libraries for structure annotation, but, typically, only a small fraction of spectra can be matched. Previous in silico methods search in structure databases but cannot distinguish between correct and incorrect annotations. Here we introduce the COSMIC workflow that combines in silico structure database generation and annotation with a confidence score consisting of kernel density *P* value estimation and a support vector machine with enforced directionality of features. On diverse datasets, COSMIC annotates a substantial number of hits at low false discovery rates and outperforms spectral library search. To demonstrate that COSMIC can annotate structures never reported before, we annotated 12 natural bile acids. The annotation of nine structures was confirmed by manual evaluation and two structures using synthetic standards. In human samples, we annotated and manually validated 315 molecular structures currently absent from the Human Metabolome Database. Application of COSMIC to data from 17,400 metabolomics experiments led to 1,715 high-confidence structural annotations that were absent from spectral libraries.

## Abstract (web summary)

COSMIC outperforms spectral library search for metabolite annotation and annotates previously unseen structures.

## Main

The discovery and elucidation of novel metabolites and natural products is cost-, time- and labor-intensive; usually, one restricts this work to a handful of compounds carefully selected via intricate prior experiments (see, for example, refs. ^1,2^). In contrast, liquid chromatography (LC) coupled to mass spectrometry (MS) allows a relatively comprehensive metabolome analysis of a biological system. LC–MS analysis can detect hundreds to thousands of metabolites from only small amounts of sample; tandem mass spectrometry (MS/MS) individually fragments the observed metabolites and records their fragment masses. Public repositories containing metabolomic LC–MS/MS data^3–5^ are growing quickly, but repurposing these data at a repository scale remains non-trivial.

Structural annotation via MS/MS is usually carried out by spectral library search, but annotations are intrinsically restricted to compounds for which a reference spectrum (usually based on commercially available chemicals) is present in the library. Despite ongoing discussions on how many detected features actually correspond to metabolites^6–8^, it is widely conjectured that a large fraction of compounds remain uncharacterized^9–11^. Beyond establishing a ranking of candidates, the score of the best-scoring candidate in the library (the hit) is used to evaluate the confidence of an annotation: a low hit score indicates that a wrong candidate has been selected, potentially because the correct answer is absent from the library. Evaluation can be carried out using ad hoc score thresholds or by statistical methods such as false discovery rate (FDR) estimation^12^.

Recently, in silico methods were developed that allow searching in substantially more comprehensive molecular structure databases^13–18^ (see Online Methods for details). In principle, in silico methods can annotate structures not present in all current structure databases, overcoming the boundaries of known (bio)chemistry: databases of hypothetical compound structures can be generated combinatorially^19–21^, by modifying existing metabolite structures^22,23^, or through machine learning^24–26^. Two requirements have to be met by an in silico method to be useful for automated annotation of compounds at a repository scale. First, it must not rely on ‘metascores’ that integrate information such as citation frequencies or production volumes into the annotation process^27^; this information is clearly not available for hypothetical, novel compounds. CSI:FingerID^15^, which is best-of-class among in silico methods^18^, does not rely on such information. Second, we have to separate correct and incorrect annotations, as is the case for library search or peptide annotation in shotgun proteomics; this allows one to concentrate downstream analysis on novel compounds most likely to be correctly annotated. Naturally, one might want to use the hit score of an in silico method to differentiate between correct and incorrect hits, as is done for spectral library search; but separation via hit scores of current in silico methods turns out to be impossible.

## Results

### Method overview

**Fig. 1:**
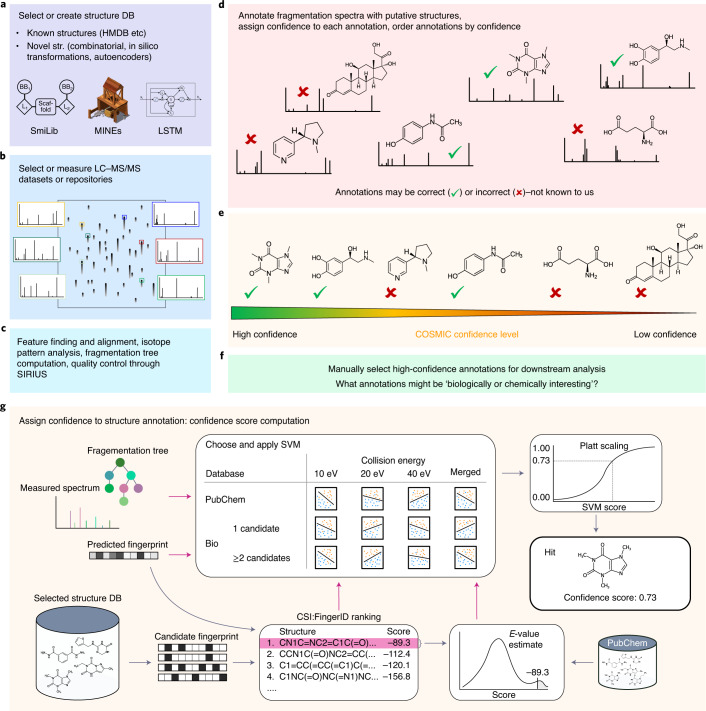
COSMIC workflow. **a**, Select or create a structure database; this can be an existing structure database such as the HMDB or generated explicitly for this purpose. **b**, Select or measure an LC–MS/MS dataset or select a complete data repository (data repurposing). **c**, Data processing through SIRIUS. **d**, Structure annotation of fragmentation spectra through CSI:FingerID; only the candidate that is top ranked by CSI:FingerID is considered. We stress that, at this point, there is no ordering of hits. **e**, Each hit (structure annotation) is assigned a confidence score; annotations are then ordered by confidence, allowing users to concentrate on high-confidence annotations. **f**, High-confidence annotations can be used to develop or test a biological hypothesis. **g**, Detailed confidence score computation for the structure annotation of a spectrum (hit) applied in **e**, including feature calculation (magenta arrows), *E* value estimation, selection and application of the appropriate SVM and Platt scaling. Notably, COSMIC can annotate metabolites at an early stage of a biological analysis. DB, database; str., structure; MINE, metabolic in silico network expansions; LSTM, long short-term memory.

Here we present the COSMIC (Confidence Of Small Molecule IdentifiCations) workflow that combines selection or generation of a structure database, searching in the structure database with CSI:FingerID and a confidence score to differentiate between correct and incorrect annotations. COSMIC can annotate a substantial fraction of metabolites with high confidence and at low FDR; our evaluations indicate that COSMIC outperforms spectral library search for this purpose, simultaneously expanding the considered compound space. COSMIC can process data at a repository scale, allowing us to repurpose the quickly growing public metabolomics data. We demonstrate this by processing 20,080 LC–MS/MS datasets using the COSMIC workflow, annotating thousands of features with structures for which, at present, no reference MS/MS data are available. Doing so, COSMIC might allow us to flip the metabolomics workflow (Fig. 1): we might concentrate on metabolites annotated with high confidence, without the need for intricate prior experiments, and try to develop a biological hypothesis from these annotations. Annotated fragmentation spectra can subsequently be searched in other datasets via ‘classical’ spectral library search at the repository scale^28^, allowing a more comprehensive annotation of public metabolomics datasets. COSMIC does not require the user to retrain it for individual datasets.

For each fragmentation spectrum, COSMIC considers only the structure candidate that is top ranked by CSI:FingerID as an annotation; COSMIC neither changes annotations (re-ranks structure candidates) nor discards any annotations. COSMIC’s confidence score combines *E*-value estimation and a linear support vector machine (SVM) with enforced directionality (Fig. 1g). First, we calibrate CSI:FingerID scores using *E*-value estimates^29^. Because it is non-trivial to generate decoys for small molecule structures, we use candidates in PubChem^30^ as a proxy of decoys. We model the score distribution as a mixture distribution of log-normal distributions and estimate *P* value and *E* value of a hit score using the kernel density estimate of the PubChem candidate scores. Second, we use an SVM to classify whether a hit is correct. Besides the calibrated score, COSMIC’s confidence score uses features such as score differences to other candidates, the total peak intensity explained by the fragmentation tree and the cardinality of the molecular fingerprints (Supplementary Table 1). To lower chances of overfitting, we restricted learning to a linear SVM; in addition, we enforced directionality of features. This means that we decided upfront whether high values or low values of a feature should improve our confidence in an annotation. For example, a high CSI:FingerID score of a hit should increase, but must never decrease, our confidence that the hit is correct (Supplementary Table 1). Some features require that there exist at least two candidates as they compare, for example, the CSI:FingerID score difference between the hit and the runner-up. For instances with only a single candidate structure, we, therefore, trained separate SVMs that do not use such features. Third, we map decision values of the SVM to posterior probability estimates using Platt scaling^31^.

### Method evaluation

The highest-ranked candidate for some query fragmentation spectrum is called a hit; it can be either the correct candidate (correct hit) or an incorrect candidate (incorrect hit). We want to decide whether a given hit is correct or incorrect; our evaluations will not consider structure candidates beyond the highest-ranked candidate. COSMIC’s confidence score is meant to separate correct hits (via high confidence score) from incorrect hits (via low confidence score). We first demonstrate that one cannot use hit scores of current in silico tools to differentiate correct and incorrect hits. We show this for four leading in silico tools that participated in the Critical Assessment of Small Molecule Identification (CASMI) 2016 contest^18^: MetFrag^13^, MAGMa+^16^, CFM-ID^14^ and CSI:FingerID^15^. For any reasonable in silico method, score distributions of correct and incorrect candidates differ substantially. However, the score of the correct candidate competes with all incorrect candidates for this query; by design, there are orders of magnitude more incorrect candidates than correct candidates (Supplementary Fig. 1). Incorrect hits result either from an incorrect candidate receiving a higher score than the correct candidate or from queries where the correct candidate is missing. Incorrect hits can have high scores, whereas correct hits might have low scores.

**Fig. 2:**
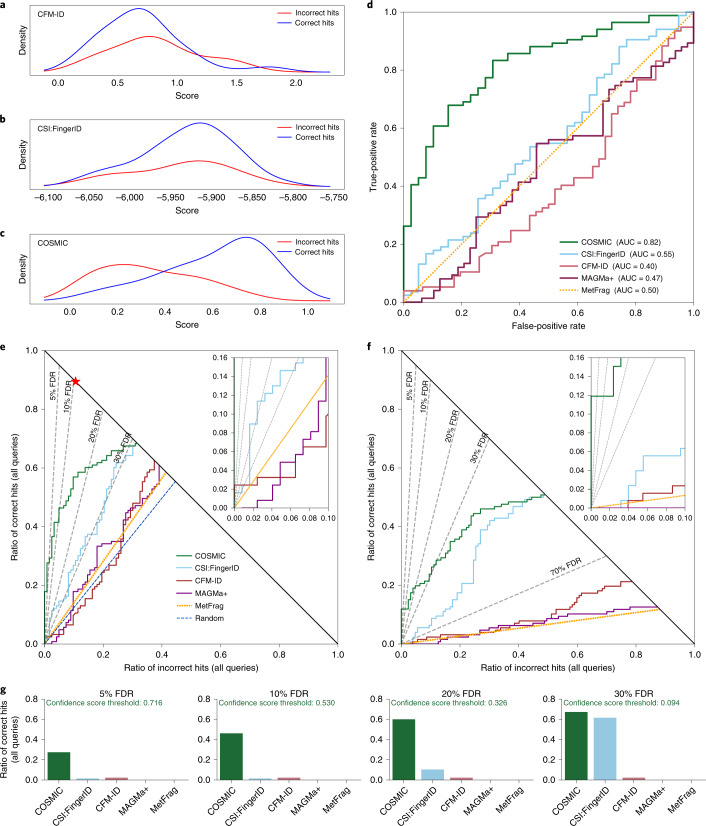
Separation by hit score for different in silico tools, using the CASMI 2016 contest submissions. Positive ion mode; candidates retrieved by molecular formula. **a**–**e**, Searching the biomolecule structure database (*n* = 123 queries). **f**, Searching in ChemSpider (*n* = 127 queries). **a**–**c**, Kernel density estimates of the score mixture distribution (correct and incorrect hits) for CFM-ID (**a**) and CSI:FingerID (**b**), ensuring structure–disjoint training data through cross-validation, and COSMIC (**c**). Kernel density estimates do not allow for a direct comparison of different tools. **d**, ROC curves for MetFrag, MAGMa+, CFM-ID, CSI:FingerID (ensuring structure–disjoint training data) and COSMIC. MetFrag normalizes scores, so the ordering of hits is exactly random. **e**,**f**, Hop plots for the same tools, searching the biomolecule structure database (**e**) or ChemSpider (**f**). FDR levels are shown as dashed lines; FDR levels are exact, not estimated (Methods). The blue dashed line in **e** indicates random scores, resulting in random ordering of candidates and hits; the red star in **e** is the best possible search result. **g**, Bar plots for the ratio of correct hits returned at FDR 5%, 10%, 20% and 30%, searching the biomolecule structure database. Again, FDR levels are exact. This information can also directly be read from the hop plot (**e**) (see Extended Data Fig. 1 for details). We also report COSMIC’s confindence score thresholds corresponding to each level. **a**–**g**, CSI:FingerID and COSMIC are computed here; all other scores are from ref. ^18^. Source data

**Extended Data Fig. 1:**
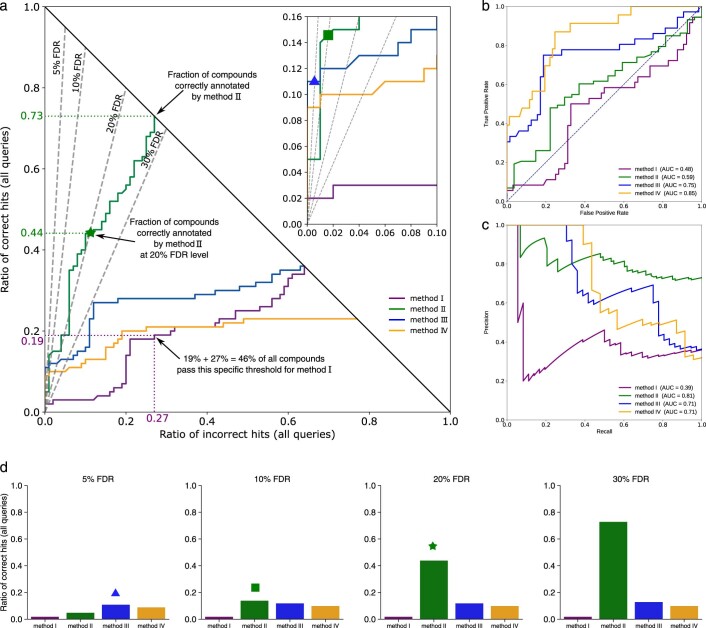
Introducing hop plots. (a) Hop plots allow us to simultaneously assess a methods annotation rate *and* its power to separate correct and incorrect hits. Two methods with identical annotation rate will end up in the same point (*x*, *y*) with *x* + *y* = 1, see methods I and III; these methods can differ substantially in their separation power. The plot shows which method performs best for a desired number of correct annotations (horizontal lines, not shown), incorrect annotations (vertical lines, not shown), or false discovery rate (FDR, dashed lines). For example, if we are willing to accept three incorrect annotations from a total of *N* = 100 queries, then method IV clearly outperforms method I; this ordering is reversed if we consider all queries (*x* + *y* = 1). FDR levels correspond to lines through the origin; a hop curve may cross or touch some FDR line multiple times, or only in the origin. We report the maximum number of correct annotations among all crossing points. For example, method II returns 55 hits (44 correct, 11 incorrect) at FDR 20 % (star). We are usually interested in small FDR values such as FDR 10 %, so a zoom-in shows where different curves cross the corresponding FDR lines: For example, method III returns 11 hits (all correct) at FDR 5 % (triangle, zoom-in), and method II returns 15 hits (14 correct) at FDR 10 % (square, zoom-in). See Online Methods for further details. (b) ROC plot and (c) precision-recall curve for the data shown in (a). Both plots (b) and (c) hide the information that method II is by far the most powerful method. (d) Bar plots for four FDR levels. Notably, the information from the bar plot can directly be read from the hop plot: We mark the corresponding values by star, triangle and square, compare to the corresponding marks in (a). Source data

For CASMI 2016, 127 synthetic standards were measured in positive ion mode using LC–MS/MS of 22 mixes on an Orbitrap instrument^18^ (see Fig. 2 for CASMI 2016 results). Receiver operating characteristic (ROC) curves allow a direct comparison of the separation power of different methods (Fig. 2d). Area under the curve (AUC) of ROC curves was between 0.40 and 0.55 for MetFrag, MAGMa+, CFM-ID and CSI:FingerID, which is not substantially better or even worse than random (AUC 0.5). In comparison, COSMIC reached AUC 0.82. ROC curves ignore the total number of correct annotations of individual methods, so AUCs can be misleading. Here we introduce hop plots (Methods and Extended Data Fig. 1), allowing us to assess the number of correct hits that a method reaches for any given FDR (Fig. 2ef). We stress that, at this point, we are considering exact FDR values, not FDR estimation (Methods). Clearly, we are particularly interested in small FDR values. Using COSMIC, we correctly annotated 57 hits with FDR below 10% when searching the biomolecule structure database (123 queries) and 16 hits with FDR 0% by searching ChemSpider^32^ (127 queries). In comparison, MetFrag, MAGMa+ and CFM-ID did not annotate a single compound at FDR 70% when searching ChemSpider, whereas using the CSI:FingerID score resulted in zero annotations at FDR 40%. Compared to the CSI:FingerID score, COSMIC improved the average rank of correct annotations from 56.5 to 33.9 when searching the biomolecule structure database (see Supplementary Fig. 2 for CSI:FingerID results without structure–disjoint evaluation).

The CASMI dataset is comparatively small, and results are prone to stochastic fluctuations; hence, we thoroughly evaluated COSMIC using two large datasets. We used ten-fold cross-validation on the COSMIC training dataset (National Institute of Standards and Technology (NIST), Orbitrap MS/MS) and an independent reference dataset with 3,291 compounds (forensics/toxicology library, Agilent, quadrupole time-of-flight (QTOF) MS/MS). We must use reference datasets for evaluation, as the correct answer is usually unknown for biological datasets. Unless indicated otherwise, all evaluations were again carried out structure–disjoint. In biological experiments, fragmentation spectra are often recorded at exactly one collision energy; to this end, we compiled three spectral libraries for individual collision energies, plus one with merged spectra from three collision energies. Furthermore, fragmentation spectra from biological samples seldom reach the same quality as reference spectra; to this end, we added (medium or high) noise to the reference spectra before evaluation. We search in a biomolecule structure database combined from several public databases. For 22.70% of the queries in cross-validation and 17.29% from independent data, the correct answer was missing from the searched structure database. Hence, COSMIC cannot correctly annotate these compounds; we did not discard these queries, as, in application, the correct true structure might, indeed, be not part of the searched database. Added noise, single collision energy spectra and unsolvable instances result in CSI:FingerID annotation rates (34.91–49.15% correct hits in cross-validation and 41.80 –59.98% on independent data) that are substantially smaller than those previously reported^15,33^.

**Fig. 3:**
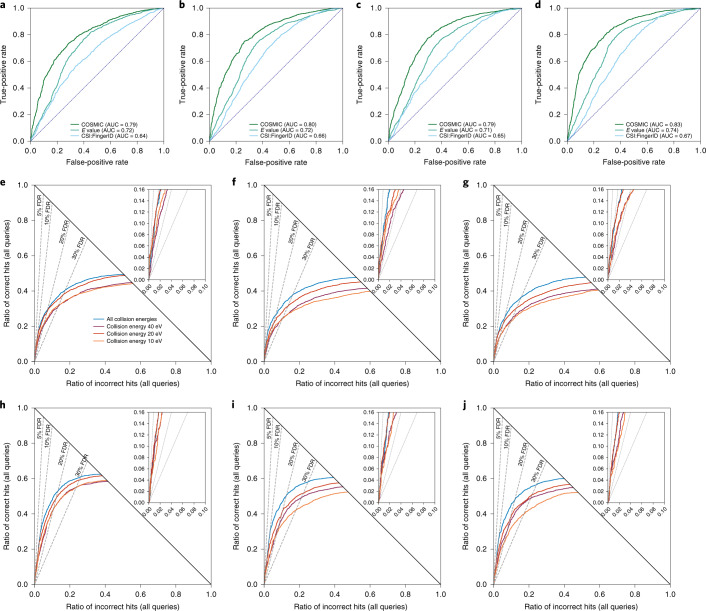
Evaluation of separation searching in the biomolecule structure database. **a**–**d**, Comparison of CSI:FingerID score, calibrated score (*E* value) and COSMIC confidence score. ROC curves, structure–disjoint evaluation, independent data and medium noise (*n* = 3,013). 10 eV (**a**), 20 eV (**b**), 40 eV (**c**) and merged spectra (**d**) (‘all collision energies’). In each plot, all curves end in the same number of correct hits (1,829 for **a**, 1,901 for **b**, 1,765 for **c** and 1,948 for **d**), so a hop plot would not contain additional information. **e**–**j**, Evaluation of COSMIC confidence score: hop plots for different collision energies. **e**–**g**, Structure–disjoint cross-validation; queries are Orbitrap MS/MS data (*n* = 3,721). **h**–**j**, Independent data with structure–disjoint evaluation; queries are QTOF MS/MS data (*n* = 3,013). No added noise (**e**,**h**), medium noise (**f**,**i**) and high noise (**g**,**j**). FDR levels are shown as dashed lines; FDR levels are exact, not estimated (Methods). Source data

**Extended Data Fig. 2:**
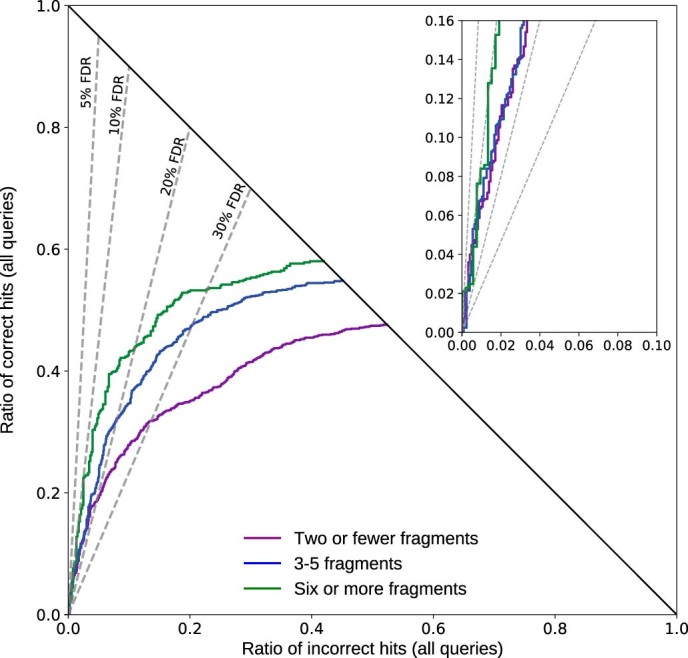
Evaluation of separation vs. number of intense peaks in the query spectrum. Independent data, 10 eV, structure-disjoint evaluation, medium noise, searching the biomolecule structure database. We binned query spectra into three categories (up to 2 fragments, 3 to 5 fragments, 6 or more fragments), based on the number of peaks in the query spectrum with relative intensity at least 5 %. We observe that the number of peaks has a clear impact on the annotation performance of CSI:FingerID, but a weaker impact on the separation performance of COSMIC’s confidence score. Source data

We empirically established that CSI:FingerID scores can be modeled as a mixture distribution of log-normal distributions (Supplementary Fig. 3). The calibrated score (*E* value) showed slightly better separation than the CSI:FingerID score (Fig. 3). We used ten-fold cross-validation for training and evaluation of the confidence score SVM and ensured structure–disjoint evaluation for independent data. See Supplementary Table 1 and Supplementary Fig. 4 for weights given to the features of the confidence score. Besides (calibrated) CSI:FingerID scores, score differences between hit and runner-up and the number of candidates turned out to be highly important features. Other features, such as a simple quality measure for the predicted molecular fingerprint or the score of the fragmentation tree, received weights close to zero. Separation between correct and incorrect hits for the confidence score is much stronger than for the (calibrated) CSI:FingerID score (Fig. 3). We found that mass had no pronounced effect on separation via the confidence score; in contrast, the number of candidates in the structure database had a strong effect (Supplementary Figs. 5 and 6). Furthermore, the number of intense peaks in a query spectrum has a clear effect on CSI:FingerID’s annotation performance but a weaker effect on COSMIC’s separation performance (Extended Data Fig. 2).

**Fig. 4:**
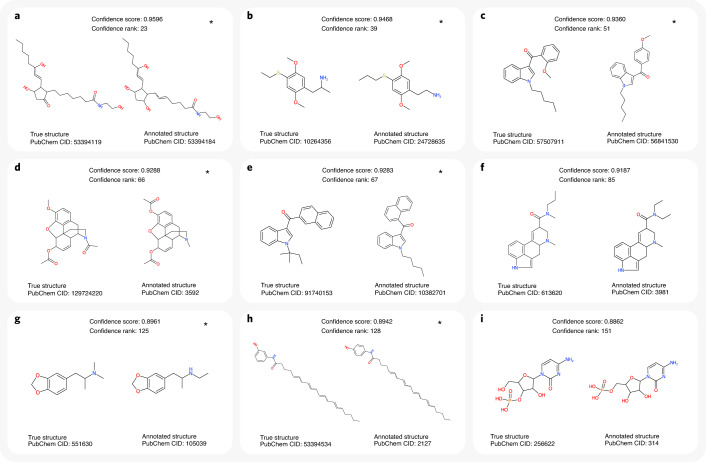
Examples of incorrect annotations with highest confidence scores. Queries are cross-validation data, merged spectra, medium noise, biomolecule structure database and structure–disjoint evaluation. Evaluations were carried out using reference spectra, so the true structure behind each query spectrum is known to us but not known to CSI:FingerID or the confidence score. Each query spectrum is annotated with the structure that is top ranked by CSI:FingerID; this pair is called ‘hit’ and can be either correct (annotation is identical to the true structure) or incorrect. All hits were then ordered by confidence score; it is inevitable that some incorrect hits will receive a high confidence score. Of the 151 hits with confidence scores above 0.8862, 142 were correct (not shown here), and only nine were incorrect (**a**–**i**). Incorrect annotation (CSI:FingerID top-ranked structure) is on the right, and corresponding true structure is on the left. Incorrect annotations might or might not be structurally similar to the true structure (compare to Extended Data Fig. 3). Notably, the nine incorrect annotations with highest confidence score (**a**–**i**) show very high structural similarity to the corresponding true structures. This is particularly noteworthy as the confidence score machine learning model has not been trained taking into account this structural similarity. If incorrect hit *i* is at rank *n*, this implies that *n* − *i* of the *n* − 1 top-ranked hits are correct, and only *i* − 1 are incorrect, corresponding to exact FDR (*i* − 1)/(*n* − 1). For example, only eight of 150 hits with highest confidence score were incorrect (exact FDR 5.33%) for confidence score threshold 0.8863. ‘Confidence rank’ is the rank of the (incorrect) hit in the complete ordered list of hits, and ‘PubChem CID’ is the PubChem compound identifier number. Instances where the true structure was not contained in the biomolecule structure database are marked by an asterisk. For these instances, a correct annotation by CSI:FingerID is impossible; at the same time, it is highly challenging for the confidence score to identify these hits as ‘incorrect’. In seven cases, molecular graphs of the incorrect hit and true structure differ by the theoretical minimum of two edge deletions. Query spectra: NIST 1210761/62/64 (**a**), NIST 1617825/29/34 (**b**), NIST 1320583/85/91 (**c**), NIST 1429464/65/71 (**d**), NIST 1483460/63/69 (**e**), NIST 1247455/57/63 (**f**), NIST 1480825/30/34 (**g**), NIST 1418771/73/80 (**h**) and NIST 1276453/55/59 (**i**).

**Extended Data Fig. 3:**
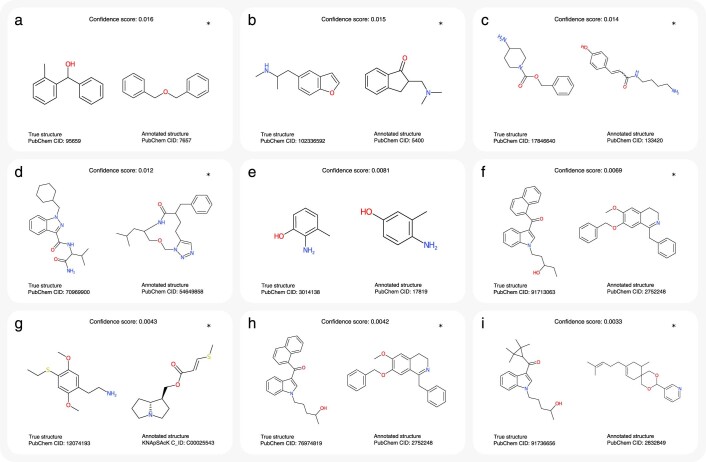
Examples of incorrect annotations with lowest confidence scores. Queries are cross-validation data, merged spectra, medium noise, biomolecule structure database, structure-disjoint evaluation. (a–i) Incorrect hits with lowest confidence scores. Top-ranked structure on the right and corresponding true structure on the left. ‘PubChem CID’ is PubChem compound identifier number. Instances where the true structure was not contained in the biomolecule structure database are marked by an asterisk. For (g), the structure of the top hit is not contained in PubChem; we report the KNApSAcK compound identifier (‘C_ID’) instead. For (a) and (e), molecular graphs of incorrect hit and true structure differ by the theoretical minimum of two edge deletions. For (a), the query spectrum was heavily distorted, and only 8.6 % of peak intensities were explained by the fragmentation tree. For (e), the three top-ranked candidates — including the correct one — were structurally highly similar and received almost identical CSI:FingerID score. Hence, COSMIC rightfully showed little confidence in these (incorrect) hits. Query spectra: (a) NIST 1544714/19/23, (b) NIST 1322859/64/69, (c) NIST 1627646/51/56, (d) NIST 1462584/87/93, (e) NIST 1340388/91/96, (f) NIST 1320854/56/62, (g) NIST 1386503/07/12, (h) NIST 1305770/72/78, (i) NIST 1325235/37/43.

**Extended Data Fig. 4:**
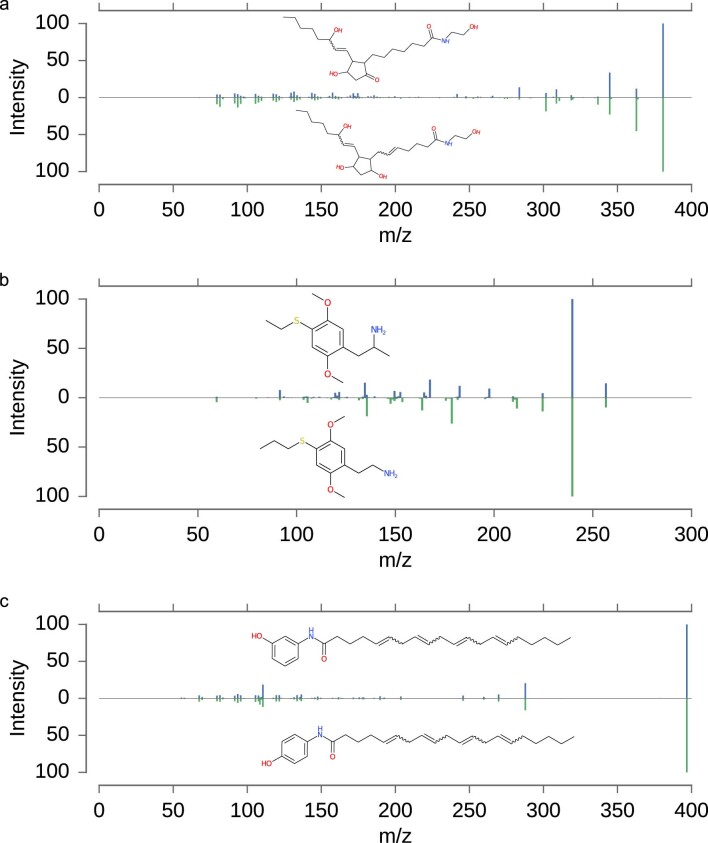
Comparison of fragmentation spectra for high-scoring incorrect hits from Fig. 4. These correspond to compound pairs where COSMIC search resulted in an incorrect hit; would spectral library search be able to avoid these incorrect annotations? For three incorrect hits from Fig. 4 (**a**,**b**,**h**) there exist merged spectra; for the remaining six incorrect hits, no such data are available. Be reminded in all three cases (**a**,**b**,**h**), the true structure was not contained in the searched molecular structure database. Merged spectrum and structure of true structure shown top, merged spectrum and structure of incorrect hit bottom. Merged spectra were combined from 10 eV, 20 eV and 40 eV spectra as described in the Methods section. (a) Mirror plot for Fig. 4a, confidence 0.9596, cosine score 0.8566. (b) Mirror plot for Fig. 4b, confidence 0.9468, cosine score 0.9432. (c) Mirror plot for Fig. 4h, confidence 0.8942, cosine score 0.9968. In all three cases, the cosine score is above 0.85, and would result in a high-confidence but incorrect library search annotation if one of the spectra was in the library, the other our query. For (c) we argue that no method could possibly distinguish between these structures based on the MS/MS data. Merged spectra: (a) correct NIST 1210761/62/64, incorrect hit NIST 1215622/23/27; (b) correct NIST 1617825/29/34, incorrect hit NIST 1386465/69/74; (c) correct NIST 1418771/73/80, incorrect hit NIST 1375293/295/301.

Inevitably, some incorrect hits received a high confidence score and, hence, would be wrongly regarded as ‘probably correct’. Figure 4 shows the nine incorrect hits with the highest confidence scores when searching independent data with medium noise. In seven of nine cases, the true structure was not contained in the biomolecule structure database. In all nine cases, the true structure was highly similar to the corresponding hit; in contrast, the bottom nine incorrect hits generally showed little structural similarity to the corresponding true structures (Extended Data Fig. 3). Notably, the confidence score machine learning model has not been trained taking into account this structural similarity. We then compared fragmentation spectra for three structure pairs from Fig. 4; for each pair, fragmentation spectra are, indeed, highly similar (Extended Data Fig. 4), with cosine score between 0.85 and 1.00. Hence, spectral library search might result in the same high-confidence misannotations.

**Extended Data Fig. 5:**
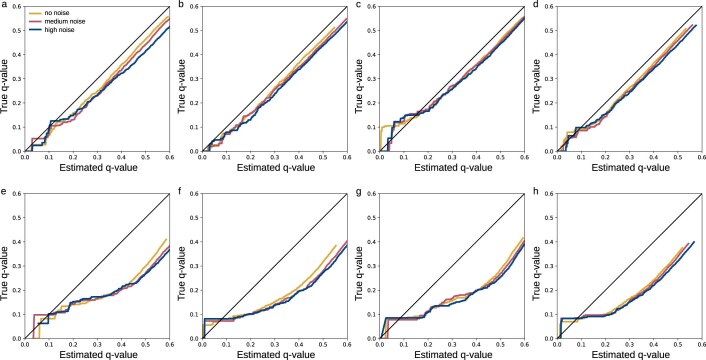
False discovery rate estimation. Q-Q plot of true vs. estimated q-values with no added noise, medium noise, and high noise. (a–d) cross-validation, *N* = 3 721. (a) 10 eV, (b) 20 eV, (c) 40 eV, (d) merged spectra. (e–h) Independent data, *N* = 3 013. (e) 10 eV, (f) 20 eV, (g) 40 eV, (h) merged spectra. The ‘step’ at the beginning of most curves in (e–h) is not an issue of FDR estimation, but due to the fact that no non-zero (true) q-values below this exist in the dataset. Source data

Recall that all FDR values reported so far are exact; FDR estimation refers to the task of estimating the exact FDR value as accurately as possible without knowing what hits are correct and incorrect. FDR estimation for small molecule annotation is highly challenging^12,34^; this is an intrinsic problem of small molecule annotation, as the assumption of incorrect hits being random is fundamentally violated^35^. We transferred COSMIC confidence scores to FDR estimates^12,36^, but, as expected, these estimates were of mediocre quality only (Extended Data Fig. 5). In particular, estimates for independent data were highly conservative: estimated *q* values were much larger than true *q* values. Consequently, confidence score values must be treated as a score but not as the probability that the annotation is correct.

We also trained classifiers for searching in PubChem instead of the biomolecule structure database. These classifiers showed a worse performance (Supplementary Fig. 7), and we observed a substantial drop of correct annotations for small FDR values. Again, COSMIC strongly outperformed both *E* values and the CSI:FingerID score.

**Extended Data Fig. 6:**
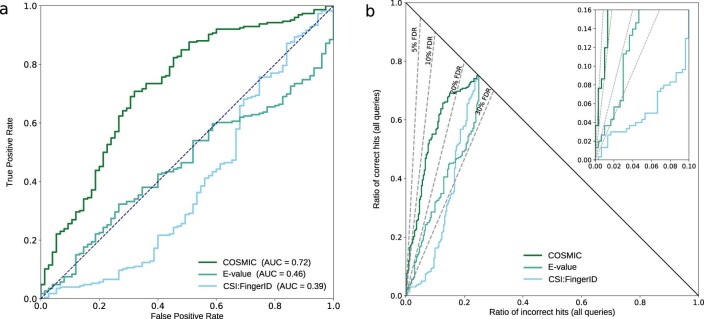
Separation results for the Sciex dataset. Comparison of CSI:FingerID score, calibrated score (E-value) and COSMIC confidence score. Positive ion mode, structure-disjoint evaluation. MS/MS spectra were recorded as ramp spectra with collision energy 20 eV to 50 eV; we used the ‘merged spectra’ model of the confidence score. (a) ROC plot and (b) hop plot for searching the biomolecule structure database, *N* = 301. FDR levels shown as dashed lines; FDR levels are exact, not estimated (Online Methods). CSI:FingerID correctly annotated 226 queries (75.1 %) in this dataset. Notably, separation by both the CSI:FingerID score and the calibrated score is worse than random on this dataset. COSMIC’s performance is particularly remarkable as the confidence score uses both the CSI:FingerID score and the calibrated score as features. COSMIC correctly annotated 23 hits with FDR below 5 %, and 166 hits with FDR below 15 %. Source data

Finally, we evaluated COSMIC using another complex mixture of synthetic standards measured by LC–MS/MS. Different from the CASMI dataset that was measured on an Orbitrap instrument, the Sciex dataset contains QTOF MS/MS data. Data were measured using 43 complex mixtures from 314 standards. Here, we observe basically the same differences in separation power between the CSI:FingerID score and COSMIC’s confidence score (Extended Data Fig. 6). Notably, both the CSI:FingerID score and the calibrated score perform worse than random for ordering hits in this dataset, whereas COSMIC annotates a large fraction of hits at small FDR.

### Evaluation against spectral library search

**Fig. 5:**
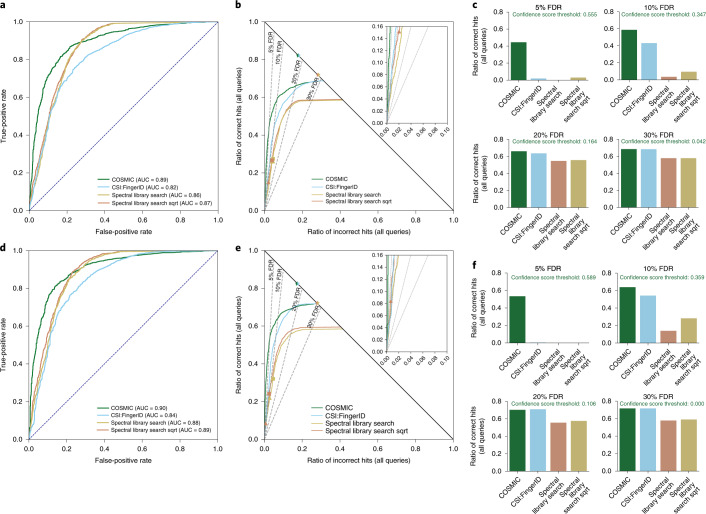
Comparison to spectral library search and separation without structure–disjoint evaluation. Query spectra (independent dataset) distorted with medium noise. COSMIC is searching the biomolecule structure database. ROC curves (**a**,**d**), hop plots (**b**,**e**) and bar plots (**c**,**f**) for collision energy 20 eV (**a**–**c**) and merged spectra (**d**–**f**). Bar plots (**c**,**f**) for FDR levels 5%, 10%, 20% and 30%. There is no overlap in fragmentation spectra between training data and independent data, but we do not remove training data for which we find the same structure in the independent dataset. To this end, 2,192 of the *n* = 3,013 structures from the independent dataset (72.75%) are also present in the spectral library. We compare search performance and separation of COSMIC, the CSI:FingerID score and spectral library search. All three methods use basically the same MS/MS data. For spectral library search, we compute the normalized dot product using either regular peak intensities or the square root of peak intensities (‘Spectral library search sqrt’)^46^. Spectral library search candidates were restricted to those with the correct molecular formula for each query. Query spectra are QTOF MS/MS data, whereas the spectral library contains a mixture of QTOF and Orbitrap MS/MS data. The spectral library is 16-fold smaller than the biomolecule structure database, giving library search a large competitive edge in evaluation. Notably, COSMIC results in substantially more correct annotations than library search for all reasonable FDR levels; FDR levels are exact, not estimated (Methods). For spectral library search, markers show commonly used cosine score thresholds 0.9 (triangle) and 0.8 (square), respectively. Finally, stars indicate the best possible annotation results, for CSI:FingerID/COSMIC and library search. sqrt, square root. Source data

**Extended Data Fig. 7:**
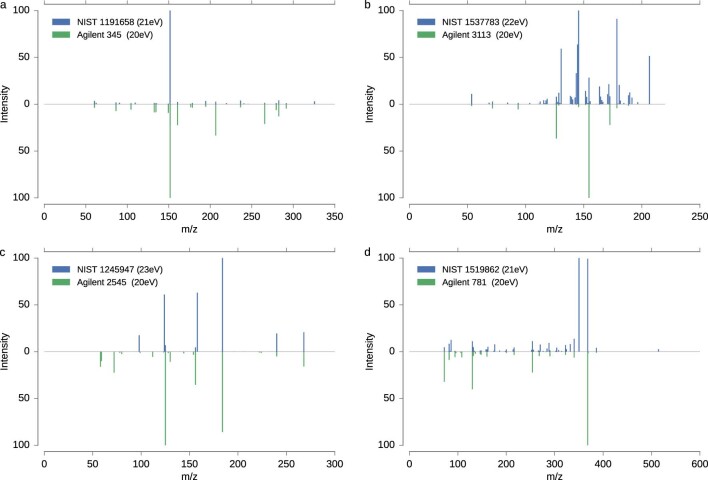
Mirror plots of low-scoring library hits that were correctly annotated with high confidence using COSMIC. Shown is the query spectrum (bottom) from the independent dataset, plus the top-scoring reference spectrum (top) from the spectral library, that is, the CSI training dataset without merging spectra. Cosine scores were calculated using regular intensities (cosine) as well as square root of intensities (cosine-sqrt). All query spectra consist of a single 20 eV collision energy measurement with medium noise added. Reference spectra consist of a single collision energy measurement with no added noise; shown is the spectrum with the *highest* cosine, among *all* spectra in the spectral library for this compound. (a) Spectra of Thiophanate, PubChem CID 3032792, molecular formula C_14_H_18_N_4_O_4_S_2_. Reference spectrum NIST 1191658, query spectrum Agilent PCDL 345. Correct COSMIC annotation with confidence 0.9092, cosine 0.0637, cosine-sqrt 0.3165. (b) Spectra of Chlorbufam, PubChem CID 16073, molecular formula C_11_H_10_ClNO_2_. Reference spectrum NIST 1537783, query spectrum Agilent PCDL 3113. Correct COSMIC annotation with confidence 0.9347, cosine 0.1949, cosine-sqrt 0.3523. (c) Spectra of Duloxetine, PubChem CID 60835, molecular formula C_18_H_19_NOS. Reference spectrum NIST 1245947, query spectrum Agilent PCDL 2545. Correct COSMIC annotation with confidence 0.9283, cosine 0.5197, cosine-sqrt 0.4767. (d) Spectra of Proscillaridin, PubChem CID 5284613, molecular formula C_30_H_42_O_8_. Reference spectrum NIST 1519862, query spectrum Agilent PCDL 781. Correct COSMIC annotation with confidence 0.9720, cosine 0.6312, cosine-sqrt 0.4852. Unlike the commercial Agilent library, the query spectra shown here are uncurated and artificial noise was added.

We also evaluated the de-replication power of COSMIC in comparison to spectral library search. For this evaluation, CSI:FingerID and the confidence score were trained without cross-validation, and query spectra came from the independent dataset. Hence, this evaluation is not structure–disjoint but still spectrum–disjoint: not a single query spectrum is part of the training data. For spectral library search, the complete training data were used as the spectral library. One might expect that targeting novel compounds (the true purpose of the COSMIC workflow) instead of de-replication comes at a price: the biomolecule structure database is more than an order of magnitude larger than Global Natural Product Social Molecular Networking (GNPS)^37^ and NIST spectral libraries, and we cannot rely on direct spectral comparison. Somewhat unexpectedly, COSMIC annotated substantially more compounds for all reasonable FDR levels (Fig. 5). At FDR 5%, COSMIC outperformed library search 1,415 hits to 52 hits at 20 eV and 1,701 hits to 1 hit using merged spectra, respectively. Notably, COSMIC correctly annotated compounds with high confidence, although query spectrum and reference spectrum were (highly) dissimilar, with cosine scores between 0.06 and 0.63 (Extended Data Fig. 7). We also observe that separation using the original CSI:FingerID score is much better than in structure–disjoint evaluations (Figs. 2 and 3). We attribute this increased separation power to the overlap in structures between training and evaluation data. Structures for which a fragmentation spectrum is present in the training data of CSI:FingerID often receive high CSI:FingerID hit scores, similarly to library search.

### Searching for novel bile acid conjugates

COSMIC allows us to expand structure annotation beyond the space of known molecules, making it possible to explore novel (bio)chemical processes. To demonstrate this, we used COSMIC to search for novel bile acid conjugates. Bile acids are amphipathic molecules that help in solubilization of lipids in the small intestine but have been found to also act as important signaling molecules^38^. Bile acids and their conjugates show a large structural diversity; exact bile acid profiles can be highly species dependent^39^. Recently, a fifth mechanism of the bile acid metabolism by the microbiome was discovered in mice/humans^40^. In that study, three novel bile acid conjugates with phenylalanine, tyrosine and leucine were found. This finding supports the possibility that other bile acids conjugated with different amino acids could exist (taurocholic and glycocholic acids are the two other known historically).

**Fig. 6:**
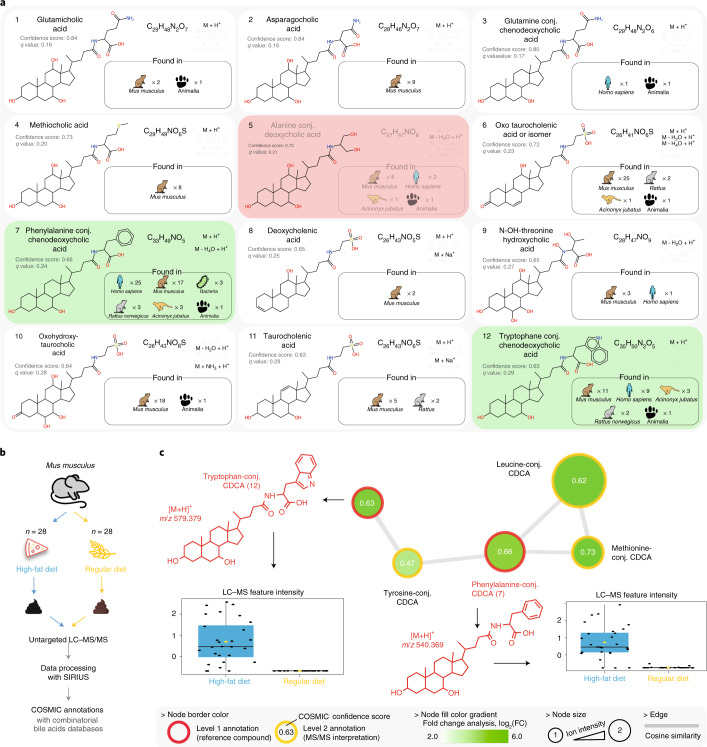
Applying COSMIC to discover novel bile acid conjugates in a mice fecal dataset. **a**, Top 12 highest-scoring COSMIC annotations of ‘truly novel’ bile acid conjugates. Bile acid conjugates that are also present in PubChem are omitted from the list; see Supplementary Table 2 for the complete list. For each bile acid conjugate, we report its chemical name, putative structure, molecular formula and adducts of annotations for this structure. In addition, we report confidence scores and estimated *q* values; note that the exact FDR is 0% for the top 4 bile acid conjugates and 8.3% for the top 12 (compare to Extended Data Fig. 5). We also report species and number of datasets with spectral matches from a MASST search. Two annotations verified by authentic standards are highlighted in green and the single incorrect annotation in red. **b**, Experimental design and the data processing and annotation carried out with COSMIC. **c**, MS-based molecular network of novel bile acid conjugates annotated with COSMIC and the combinatorial bile acids structure database. Two annotations (7 and 12) were validated using synthetic standards, and the other annotations were manually inspected. Fold change analysis showed that all these bile acid derivatives were predominantly observed in mice fed an HFD. Box plots depict the first and third quartiles as well as the median. Whiskers extend to the smallest and largest value but no further than 1.5× interquartile range from the hinges. *n* = 56 independent biological experiments. conj., conjugate; FC, fold change. Source data

**Extended Data Fig. 8:**
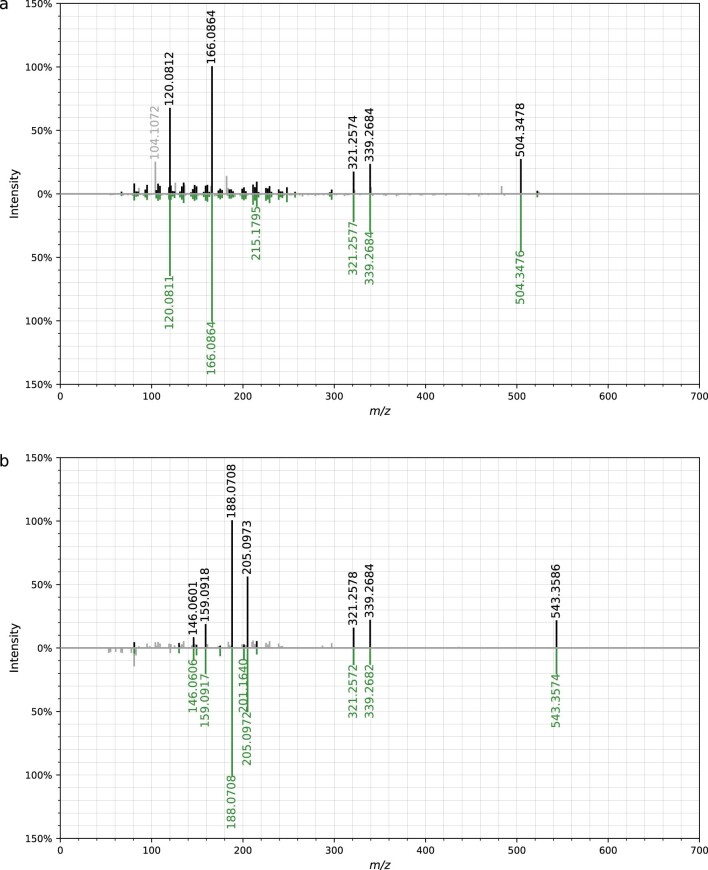
Mirror plots of fragmentation spectra for novel bile acid conjugate annotations. Query spectra above, reference spectra below the x-axis. Reference and query spectra of Phe-CDCA **7** (a) and Trp-CDCA **12** (b). Reference and query spectra were both measured on Q Exactive Orbitrap instruments. See Online Methods for the comparison of retention times.

We explored this hypothesis applying COSMIC to a public mice fecal metabolomics dataset. Plausible bile acid conjugate structures were computed by combinatorially adding amino acids to bile acid cores, yielding 28,630 plausible bile acid conjugates. The COSMIC workflow was then applied to search the combinatorial bile acid conjugate structure database. CSI:FingerID annotations were ordered by COSMIC confidence score. Processing the dataset took 5 h of wall clock time, using a compute node with 96 cores. To establish which of the annotated structures were ‘truly novel’ (Supplementary Table 2 and Supplementary Data 1), the structures were searched in PubChem, and known structures were discarded; the top 12 best-scoring ‘truly novel’ structures proposed by COSMIC (Fig. 6a) were verified by manual interpretation of the fragmentation spectra (Supplementary Figs. 8–19). In addition, we synthesized two annotated structures to validate annotations of phenylalanine-conjugated chenodeoxycholic acid (Phe-CDCA, 7 in Fig. 6a) and tryptophan-conjugated chenodeoxycholic acid (Trp-CDCA, 12) (Extended Data Fig. 8).

Inspection of the fragmentation spectra showed characteristic fragment ions for the bile acid core structure: *m/z* 337.2526 and *m/z* 319.2420 for cholic acid derivatives and *m/z* 339.2682 and *m/z* 321.2577 for a dehydroxylated bile acid core structure, presumably chenodeoxycholic acid (CDCA) given the validation for 7 and 12. The nature of the amino acid residue was confirmed from the observation of the specific amino acid fragments. First, we observed novel bile acids derivatives conjugated for the newly discovered conjugation^40^ with phenylalanine (*m/z* 166.0863, Phe-CDCA, 7). Most significantly, COSMIC enabled the discovery of completely novel amino acids bile acid conjugations. This includes bile acids conjugated with glutamine (fragment *m/z* 147.0764, glutamicholic acid 1 and Glu-CDCA 3), asparagine (fragment *m/z* 133.0608, asparagocholic acid, 2), methionine (fragment *m/z* 150.0583, methiocholic acid, 4) and tryptophan (fragment *m/z* 205.0972, Trp-CDCA, 12). In addition, a bile acid conjugated with a non-canonical amino acid was annotated (N-OH threonine, fragment *m/z* 134.0440, 9). Other annotated derivatives had modified bile acid cores, including dehydration/reduction/oxidation, and were supported by the analysis of the fragmentation pattern as for putative oxotaurocholenic acid (6), deoxycholenic (8), oxohydroxytaurocholic acid (10) and taurocholenic acid (11). A single COSMIC annotation was incorrect (5). Inspection revealed that it was wrongly interpreted as an in-source fragment. However, the expected fragment for a serinol-conjugated bile acid was not observed (Supplementary Fig. 12), and further interpretation of the fragmentation spectrum supported that it was likely the protonated ion of the alanine-conjugated CDCA.

Molecular networking analysis (Fig. 6b,c) showed that the validated annotations (7 and 12) were part of a molecular network including leucine-conjugated CDCA as well as other CDCA conjugates annotated by COSMIC but not among the top 12—namely, methionine-conjugated CDCA and tyrosine-conjugated CDCA. Inspection of corresponding fragmentation spectra showed that the COSMIC annotations were consistent (Supplementary Figs. 20 and 21). These bile acid conjugates were predominantly observed in the high-fat diet (HFD) group, and similar results were observed for the other top 12 novel bile acid conjugates (Supplementary Fig. 22). Previous research showed strong perturbation of bile acid secretion in mice subjected to an HFD^41^. Different patterns were observed for host-produced primary standard bile acids (cholic acid, ursocholic acid and muricholic acid) and microbially produced secondary bile acids (deoxycholic acid, hyodeoxycholic acid and chenodeoxycholic acid) (Supplementary Fig. 23). These secondary bile acids were relatively depleted in the HFD group, whereas primary bile acids were relatively stable among the experimental groups. Notably, glycocholic acid, the well-known host-produced conjugated bile acid, also had a stable intensity among groups, whereas taurocholic acid intensity was higher in the HFD group. To further interpret these results, we calculated the ratio between each of the top 12 novel bile acid conjugates versus three bile acids highly abundant in mice feces (deoxycholic acid, hyodeoxycholic acid and taurocholic acid). We found that ratios were systematically higher in the HFD group (Supplementary Figs. 24–26). We also investigated the three novel microbially produced bile acid conjugates from ref. ^40^ (Supplementary Figs. 27 and 28). Notably, these bile acid conjugates show similar relative abundance profiles as the top 12 novel bile acid conjugates. Microbially produced secondary bile acids are relatively depleted in the HFD group; as gut microbiota are altered by an HFD diet^42^, our results suggest the involvement of microbial species in the production of the top 12 novel bile acid conjugates (compare to ref. ^40^).

The novel bile acids were searched in all the public mass spectrometry data repositories^37^ by performing a search using the Mass Spectrometry Search Tool (MASST)^28^. Matching fragmentation spectra were observed in public datasets (Fig. 6a), predominantly consisting of data from animal fecal samples, mostly from rodents and humans. A match for Phe-CDCA (7) was observed in a bacterial culture of two opportunistic pathogens (*Escherichia coli* and genus *Stenotrophomonas*) and resonates with previous findings on the fifth mechanism of bile acid metabolism by the microbiome^40^.

### Repository-scale annotation of novel metabolites

**Extended Data Fig. 9:**
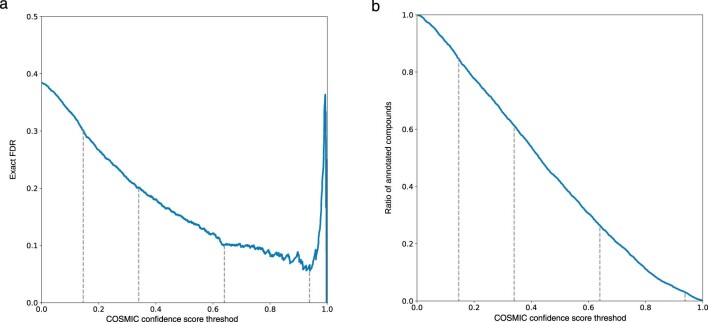
COSMIC confidence score vs. exact FDR and ratio of annotated compounds. Independent data (Agilent, QTOF), 20 eV, medium noise, *N* = 3, 013. We vary the confidence score threshold and present the resulting exact FDR (a) and the ratio of annotated compounds (b). Dashed lines indicate COSMIC confidence score thresholds of 0.94, 0.64, 0.34, and 0.14, corresponding to exact FDR levels of rougly 5 %, 10 %, 20 %, and 30 %, respectively. The spike for high tresholds beyond 0.9 is an artifact of the small number of hits that pass this threshold; hence, a few incorrect hits with high confidence score can lead to high FDR. In practice, confidence scores depend on numerous factors such as the overall quality of the data and the identity of the query compounds. Hence, these thresholds come with no guarantee in either direction: For example, in the CASMI 2016 dataset, a smaller confidence score threshold of 0.53 corresponded to exact FDR 10 %, and using the abovementioned threshold of 0.64 would have returned fewer hits than possible. Nevertheless, these thresholds may serve as a starting point for practitioners. Source data

**Extended Data Fig. 10:**
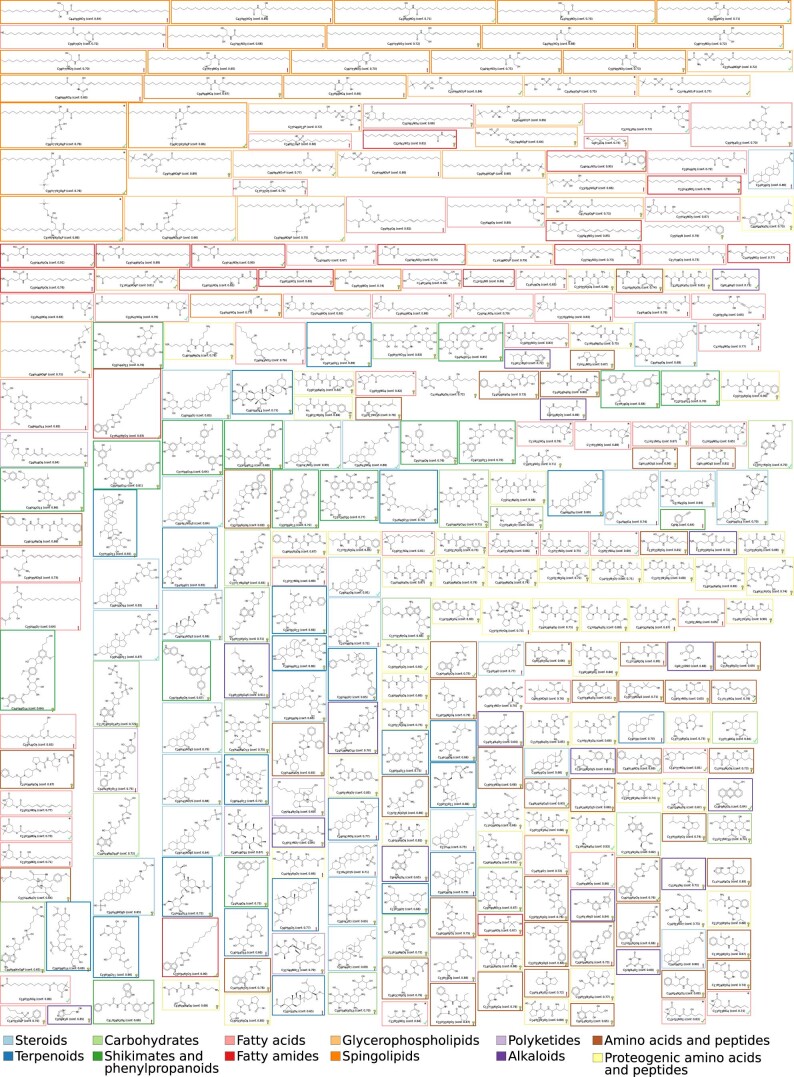
The 315 molecular structures not contained in HMDB annotated with high confidence in the human dataset. Confidence score threshold 0.64 was used. For none of these structures, reference MS/MS data are available. Structures are shown with identification number (ID), molecular formula and COSMIC confidence score. Structures present in the latest version of HMDB (Feb 2021) are marked by an asterisk. Colors indicate compound classes. Notably, 48 compounds were annotated as proteinogenic peptides; these structures were absent from HMDB but are clearly no novel metabolite structures. Lipid structures must be interpreted with some care: It is understood that neither COSMIC nor any other method can deduce, say, the position of the double bond in a carbon chain from MS/MS data alone; rather, this happens to be the candidate present in our biomolecule structure database.

The Human Metabolome Database (HMDB)^43^ contains the by far most comprehensive collection of molecular structures found in or on the human body, with version 4.0 encompassing 114,265 structures. However, certain molecular structures connected to human metabolism might currently be missing from this database. To test this hypothesis, we searched the human dataset against the biomolecule structure database; this comprises ten MassIVE datasets^37^ with 2,666 LC–MS/MS runs from different sources (serum, plasma, lips, tongue, teeth, fecal and urine). We used a confidence score threshold of 0.64, roughly corresponding to FDR 10% (Extended Data Fig. 9). We concentrated on those hits with structures absent from the HMDB. This resulted in 436 high-confidence structure annotations; 121 (27.8%) of the structures were present in our MS/MS training data, leaving us with 315 structures for which no MS/MS reference data are available (Extended Data Fig. 10 and Supplementary Table 3). The HMDB database being used for excluding structures dates back to August 2018; since then, at least 26 of these structures were added to the HMDB. This indicates that many of the novel structures are, indeed, present in human samples. We manually verified the 315 structures, of which 48 are proteinogenic peptides (peptides made from proteinogenic amino acids), by checking common neutral losses and fragments and by comparison of spectra against reference spectra from similar compounds. Based on characteristic fragmentation patterns, different acyl-carnitines and N-acyl-amino acids not part of the HMDB were annotated. N-acyl amino acids are well-known uncoupling agents in mitochondria^44^. From 30 spectra annotated as acyl-carnitines with high confidence, 21 were presumably correct based on manual verification. N-oleyl-leucine represents one particular example of an N-acyl amino acid annotated with high confidence; the annotation was verified using a reference spectrum that was not part of the COSMIC or CSI:FingerID training data (Supplementary Fig. 29). A MASST search^28^ in GNPS gave 84 datasets putatively containing a similar spectrum, 38 being human datasets. For two additional high-confidence hits, reference spectra were available and showed high similarity to the query spectra (Supplementary Fig. 29). Hits are available at https://bio.informatik.uni-jena.de/cosmic/; users can view, discuss and verify annotated structures there.

To further demonstrate COSMIC’s power to annotate metabolites at a repository scale, we then searched the Orbitrap dataset, consisting of 123 MassIVE datasets^37^ and 17,414 LC–MS/MS runs (Supplementary Table 4), against the biomolecule structure database. We again used a confidence score threshold of 0.64. This resulted in 3,530 metabolite structures annotated with high confidence, of which 1,815 were present in the training data. Discarding those, we are left with 1,715 novel structure annotations (Supplementary Fig. 30 and Supplementary Table 5); for comparison, the data used to train CSI:FingerID and COSMIC comprise 16,703 structures. Again, hits are available at https://bio.informatik.uni-jena.de/cosmic/.

We prepared spectral libraries from the above-mentioned high-confidence annotations. Processing the two datasets took 4 d of wall clock time for the human dataset and 21 d for the Orbitrap dataset, using a compute node with 96 cores. On average, a single LC–MS/MS run was processed in less than 2 min. For comparison, we processed one ‘typical’ MassIVE dataset with 44 LC–MS/MS runs on a laptop computer; this took 150 min of wall clock time.

## Discussion

Annotation scores of current in silico tools are not suited to separate correct from incorrect hits. Here we introduced the COSMIC workflow that assigns confidence scores to structure annotations. We thoroughly evaluated COSMIC using multiple spectral libraries, LC–MS/MS runs of standards and biological data, including the manual confirmation of 11 novel bile acid conjugates annotated in mice fecal samples. Annotation rates as well as separation were consistently better when using merged spectra, usually followed by 40-eV fragmentation spectra. COSMIC clearly outperformed spectral library search for de-replication; this is notable, as COSMIC has not been designed or optimized for this task. Remember that, in our evaluations, only the exact structure was regarded as correct; however, small structure modifications (Fig. 4) are hard and potentially impossible to tell apart using MS/MS data alone. This is an intrinsic limitation not of COSMIC but of small molecule MS/MS in general and requires orthogonal information to overcome; however, these incorrect annotations often contain viable structure information. Indeed, COSMIC’s incorrect annotations with high confidence are often structurally highly similar to the true structure; these incorrect annotations can, therefore, still be valuable for the structural annotation of a compound.

We demonstrated that COSMIC can be used to search for novel metabolites and rapidly test biological hypotheses. More specifically, we found additional amino acid conjugation of bile acids beyond those previously identified by repurposing public datasets^40^, opening the gate for studying their precise structure and biological relevance. Notably, more than 90% of the annotations for the top 12 bile acid conjugates suggested by COSMIC turned out to be correct. These annotations will help to further explore bile acid metabolism. Only the glycine and taurine bile acid conjugates were previously known in humans, despite 170 years of research in that field^38^.

We further demonstrated COSMIC’s power by repurposing data from 20,080 LC–MS/MS runs, providing high-confidence hits in a biomolecule structure database; for 49% of these hits, no reference spectra were available in our training data. In particular, we annotated 267 metabolites in human datasets absent from the HMDB with no reference MS/MS data available, compared to 108 such metabolites with reference MS/MS data. Annotations may now serve as starting points for generating biological hypotheses or to expand existing spectral libraries. We have used a threshold of 0.64 for the confidence score, roughly corresponding to FDR 10%; this number might serve as a practical guidance but is clearly no guarantee in either direction (Extended Data Fig. 9).

Notably, COSMIC complements compound class annotation tools such as CANOPUS^45^. COSMIC targets molecular structure annotations but annotates only a fraction of the compounds in a sample; in contrast, CANOPUS annotates practically all compounds in a sample for which fragmentation spectra have been measured but is restricted to annotating compound classes. Hence, both methods provide viable information; which method is better suited depends on the underlying research question.

COSMIC’s confidence score must not be mistaken as the probability that an annotation is correct; this is impossible by design of the score. We speculate that accurate FDR estimation from fragmentation spectra of small molecules will remain highly challenging.

## Methods

### General considerations

Establishing the stereochemistry from fragmentation spectra is highly challenging and beyond the power of automated search engines; hence, only the two-dimensional structure is considered when evaluating a hit structure. We consider the identity and connectivity (with bond multiplicities) of the atoms but ignore the stereo-configuration for asymmetric centers and double bonds.

The term ‘novel compound’ has previously been used to describe conflicting and imprecisely defined concepts, such as when an unexpected compound is detected in a sample or organism or whether compounds have previously been described in the literature. Throughout this paper, a structure is considered ‘novel’ if no MS/MS data from a compound with the same structure are present in the training data; hence, the compound cannot be annotated through spectral library search. We noted above that stereoisomers (compounds with identical structure, such as L-threose, D-threose, L-erythrose and D-erythrose) show highly similar fragmentation. Hence, for L-threose to be novel, the training data must not contain MS/MS data for L-threose, D-threose or (L- or D-)erythrose. In our evaluations, we ensure that all compounds are novel using structure–disjoint cross-validation.

Similarly, a ‘truly novel’ compound refers to a compound structure absent from large public databases such as PubChem^30^ or ChemSpider^32^; quotation marks are in place, as the (non-public) database GDB-17 (ref. ^47^) contains 166 billion hypothetical structures of small molecules, and ‘truly novel’ compounds might already be in there. For CSI:FingerID and other in silico methods that do not rely on metascores, there is no difference to search in a database of ‘truly novel’ hypothetical structures or to search in PubChem or the biomolecule structure database. It is understood that correct annotation rates will deteriorate if the database we search in becomes too large.

COSMIC targets biomolecules—that is, products of nature as well as synthetic products with potential bioactivity, including drugs, toxins, food, cosmetics and other xenobiotics. This restriction of focus is due to the available MS/MS training data.

Regarding COSMIC and its annotations, it must be understood that COSMIC only proposes structure annotations; the user has to decide which of these putative annotations will be analyzed further and potentially verified using orthogonal data, such as retention time, comparison with synthetic standards, spike-in experiments, isolation or nuclear magnetic resonance (NMR) experiments.

### FDRs

Given a list of hits, the FDR of this list is the number of incorrect hits in the list divided by the size of the list. Hence, to compute FDR, we must know the exact number of correct and incorrect hits. Throughout this paper, evaluations were carried out using reference data so that the true structure underlying any query spectrum was unknown to the method but known to us. To this end, all reported FDR rates are exact, unless indicated otherwise. At this point, there is no need to employ methods for FDR estimation^12,34,48,49^; such methods try to accurately estimate the exact FDR in application, where we do not have knowledge of correct and incorrect hits. Accurate FDR estimation remains a highly non-trivial problem in general statistics as well as many fields of application (see also below).

### In silico methods and related work

So-called ‘in silico methods’ allow us to search in a molecular structure database using MS/MS data as our query. Most methods follow one of three paradigms. (1) Combinatorial fragmenters^13,50–52^ try to explain the query spectrum using the candidate structure, combinatorially breaking bonds in the molecular structure graph. (2) Other methods try to predict the fragmentation spectrum of a given compound structure^14,53,54^; this allows us to search in the structure database by spectral matching. (3) Alternatively, we can transform the query spectrum into information about the query structure and then use this structure information to search in the structure database^15,55,56^. Later publications basically present minor modifications of these ideas; an exception is the Input Output Kernel Regression (IOKR) variants of CSI:FingerID^17,57^, which use molecular fingerprints but circumvent the prediction of individual molecular properties, instead predicting similarity of a candidate to the query by regression.

Some methods use so-called ‘metascores’ that integrate information about citation frequencies or production volume^51,58,59^. We stress that ‘metascores’ have nothing in common with ‘metadata’, except for the prefix; metadata are information about the experimental setup and the biological sample, whereas these metascores use side information unrelated to the actual experiment. These metascores usually perform well in evaluations but come with several severe restrictions; in the context discussed here, the most important restriction is that the above side information is not available, and metascores, therefore, are not applicable for any ‘truly novel’ structure, such as novel bile acid conjugates. Furthermore, metascores tend to prefer highly cited ‘blockbuster metabolite’ candidates; hence, evaluation results, which are carried out using mainly such ‘blockbuster metabolites’, are often exaggerated. Similar limitations are associated with metascores based on taxonomy^60^ as, again, this information is not available for ‘truly novel’ structures. Thus, we ignored metascore methods in our evaluations.

Finally, some tools use networks for structure annotations; networks may be based on spectral similarity in the LC–MS/MS run or structural similarity in the metabolite database^60–63^.

### Structure databases

Different from previous studies^15,33^ where structures were derived from International Chemical Identifier (InChI) strings, molecular structures were standardized using the PubChem standardization procedure^30^. In particular, a canonical tautomeric form was chosen, as solvent, temperature and pH in the sample influence the dominating tautomeric species. Standardization of compounds not in PubChem was carried out using the web service at https://pubchem.ncbi.nlm.nih.gov/rest/pug/. PubChem standardization has changed multiple times over the last years without further noticing of users; to this end, it is possible that some non-PubChem compounds were standardized slightly differently than structures from the MS/MS training data.

We searched in the following structure databases with COSMIC:

- For the CASMI 2016 evaluation^18^, we downloaded structures from the CASMI 2016 results web page (http://casmi-contest.org/2016/). Candidate structures were provided as part of the blinded contest and originally retrieved from ChemSpider^32^.
- The biomolecule structure database is a union of several public structure databases, including HMDB^43^, ChEBI^64^, KEGG^65,66^ and UNPD^67^. The resulting database contains 391,855 unique structures of biomolecules and compounds that can be expected to be present in biological samples.
- The HMDB structure database^43^ was downloaded on 8 August 2018 and contains 113,983 compounds and 95,980 unique structures with mass up to 2,000 Da.
- The PubChem structure database^30^ was downloaded on 16 January 2019 and contains 97,168,905 compounds and 77,153,182 unique covalently bonded structures with mass up to 2,000 Da. We added all missing structures from the biomolecule structure database, which resulted in a total of 77,190,484 unique structures.
- A combinatorial database of 28,630 bile acid conjugate structures was generated with SmiLib v2.0 (refs. ^19,20^), downloaded from http://melolab.org/smilib/. SmiLib generates chemical structures by combining scaffolds and building blocks provided as SMILES (Simplified Molecular Input Line Entry Specification). We curated a list of initial bile acid ‘scaffolds’ that represent common steroid cores (that is, cholic acid, deoxycholic acid, hyocholic acid and chenodeoxycholic acid). Initial scaffolds were modified manually with common phase 2 metabolism reactions (that is, glucuronidation, acetylation, sulphation and methylation) and resulted in 322 scaffolds. To generate bile acid conjugates, scaffolds were combined with 91 building blocks, including proteinogenic and non-proteinogenic amino acids, along with their N-hydroxylated and N-methylated version, and acyls moieties. Stereochemical information was removed before the database generation with SmiLib. Notably, the bile acid conjugate structure database also contains unconjugated bile acids; for the sake of brevity, we will, nevertheless, refer to ‘bile acid conjugates’ without explicitly mentioning this fact.

### MS/MS reference datasets and noise addition

For evaluations, we limited ourselves to MS/MS spectra recorded in positive ion mode, as there are generally more such spectra available. This is not a restriction of COSMIC, and the publicly available version can also process negative ion mode data. Evaluations were carried out using reference measurements, as we do not know the correct answers for biological datasets.

For the CASMI 2016 evaluation, MS/MS spectra were downloaded from the CASMI web page (http://casmi-contest.org/2016/). MS/MS spectra were measured on a Q Exactive Plus Orbitrap (Thermo Fisher Scientific) with 20/35/50 HCD nominal collision energies. Twenty-two mixes of synthetic standards were measured in one LC–MS run each, using data-dependent acquisition mode and inclusion lists. Each mix contained 10–94 compounds. A reversed-phase C18 column was used (see ref. ^18^ for details). In full, MS/MS data of 127 compounds measured in positive ion mode were provided as part of the contest. Fragmentation spectra from different collision energies were merged.

For the Sciex dataset, authentic standards from different compound libraries and single reference standards were used. Specifically, the Agilent LC/MS Pesticide Comprehensive mix, Sigma-Aldrich Bile Acid/Carnitine/Sterol Metabolite Library of Standards, Sigma-Aldrich Fatty Acid Metabolite Library of Standards and Sigma-Aldrich Acid Metabolite Library of Standards were used. Standards were dissolved in suitable solvents and mixed in 43 mixtures in such a way to avoid overlap of isomeric and isobaric substances. Standard mixtures were analyzed using a Sciex Exion AD liquid chromatography system coupled to a Sciex X500R QTOF MS system. Separation was achieved on a Phenomenex Kinetex F5 column (150 mm × 2.1 mm ID, 2.6 μm particle size) with a gradient from eluent A (100% H_2_O + 0.1% formic acid) to eluent B (100% acetonitrile + 0.1% formic acid) using the following gradient: 100/0 at 0 min, 100/0 at 2.1 min, 5/95 at 14 min, 5/95 at 16 min, 100/0 at 16.1 min and 100/0 at 20 min. Column temperature was set to 30 ^∘^C and flow rate to 200 μl min^−1^. Data were acquired by data-dependent acquisition of MS/MS spectra using a collision energy ramp from 20 eV to 50 eV. The MS was automatically recalibrated every five injections in MS1 and MS/MS mode. MS/MS spectra for the standards were extracted using the Sciex OS 2.0 software and stored as a .txt file. SIRIUS .ms files and MassBank records were generated using a custom R script.

To train CSI:FingerID, we used a combined dataset from MassBank^68^, GNPS^37^ and the NIST 2017 database. Reference MS/MS data were measured on different high-resolution instruments from multiple vendors. The CSI training dataset contains 16,703 structures with 23,965 independent MS/MS measurements. As an independent dataset, we used the commercial MassHunter Forensics/Toxicology PCDL library (Agilent Technologies) with 3,243 structures and 3,462 independent MS/MS measurements, all measured on an Agilent QTOF instrument. Unlike the commercially available library, these mass spectra were not curated. When discussing reference dataset evaluations, independent MS/MS measurements will be referred to as ‘compounds’ for the sake of brevity.

Previous evaluations of CSI:FingerID^15,33^ were carried out using fragmentation spectra that merged all available collision energies. Here, we also want to evaluate COSMIC’s power if query spectra are recorded at a single collision energy, because LC–MS/MS datasets are often recorded in this way. To this end, we compiled fragmentation spectra sets for both training and independent data using single collision energies—namely, 10 eV, 20 eV and 40 eV. To ensure that COSMIC results are comparable among different collision energies, we used only those compounds for which all three collision energies are available. In the independent data, this is the case for all compounds; but, in the training data, only NIST entries pass this criterion. Hence, the COSMIC training dataset exclusively contains spectra from NIST, all of which were measured on an Orbitrap instrument; and, consequently, all cross-validation results on this dataset exclusively use MS/MS data from Orbitrap instruments. In case the NIST library did not contain fragmentation spectra for the exact collision energies 10 eV, 20 eV and 40 eV, we allowed for a deviation of up to 4 eV; in case fragmentation spectra for more than one collision energy were present in this interval, we used the one with collision energy closest to the desired one. Finally, merged spectra were generated by combining these three spectra (pseudo-ramp spectra).

Fragmentation spectra in reference libraries often have much better quality (more signal peaks, fewer noise peaks and better signal-to-noise) than fragmentation spectra from a biological LC–MS/MS run. To simulate this effect in our reference datasets, we ‘added noise’ to each fragmentation spectrum. Distorting spectra followed similar principles as the generation of decoy spectra^12^: we distorted spectra similar to what we expect for experimental spectra. For example, adding noise peaks with (uniform) random mass will result in spectra that are notably different from experimental ones^12^. We simulated two noise models: medium noise and high noise.

- We simulated a global mass shift (bias) by drawing a random number δ^*^ from ${{{\mathcal{N}}}}(0,{\sigma }_{\,{{\mbox{mb}}}\,}^{2})$ and then shifting every peak mass *m* by δ^*^ *m*. The standard deviation *σ*_mb_ was chosen as *σ*_mb_ = (10/3) × 10^−6^ (medium noise) or *σ*_mb_ = (15/3) × 10^−6^ (high noise), so that the 3*σ*_mb_ interval represents a 10-ppm shift for medium noise and a 15-ppm shift for high noise.
- We simulated individual mass deviations by drawing, for each peak with mass *m* individually, a random number *δ* from ${{{\mathcal{N}}}}(0,{\sigma }_{\,{{\mbox{md}}}\,}^{2})$ and shifting the peak by *δ* *m*. The standard deviation *σ*_md_ was chosen so that the 3*σ*_md_ interval represents a 10-ppm shift for medium noise and a 20-ppm shift for high noise.
- We simulated intensity variations in the spectrum: each peak intensity was multiplied by an individual random number *ϵ* drawn from ${{{\mathcal{N}}}}(1,{\sigma }_{\,{{\mbox{id}}}\,}^{2})$. Variance was chosen as ${\sigma }_{\,{{\mbox{id}}}\,}^{2}=1$ for medium noise and ${\sigma }_{\,{{\mbox{id}}}\,}^{2}=2$ for high noise. Furthermore, 0.03 times the maximum peak intensity of the spectrum was subtracted from each peak intensity. If a peak intensity fell below the threshold of one thousands of the maximum intensity in the spectrum, the peak was discarded.
- Finally, we added ‘noise peaks’ to the spectrum. As uniformly choosing the mass of a noise peak would result in peaks that are too easy to spot and sort out by our subsequent analysis^12^, we, instead, used peaks that appeared in other measured spectra. In pre-processing, a pool of ‘noise peaks’ was gathered from the fragmentation spectra, using all peaks that did not have a molecular subformula decomposition of the known molecular formula of the precursor. For each spectrum, *α* *n* of these ‘noise peaks’ were added to the spectrum, where *n* is the number of peaks in the spectrum and *α* = 0.2 for medium noise and *α* = 0.4 for high noise. Intensities of ‘noise peaks’ were adjusted for maximum peak intensities in the contributing and receiving spectrum.

Parameters for medium noise and high noise were chosen in a way that the similarity between the original spectrum and the distorted spectrum reached a particular level, measured by the cosine score (dot product); for the cosine score, we allowed a mass deviation of 7 ppm when matching peaks. Precursor ion peaks were not considered for cosine score calculation, as their high intensities overshadow the lower-intensity peaks. For medium noise, the cosine score between the original and the distorted spectrum had a median value of 0.880. For high noise, the median cosine score was 0.714. Datasets with different noise levels were used for evaluations only but not to train CSI:FingerID or individual confidence score SVMs.

Adding noise to the fragmentation spectra might result in an empty or almost empty spectrum, which would be regarded as insufficient for structure annotation in applications. To this end, we removed fragmentation spectra with, at most, one peak. To ensure that evaluation results are comparable between collision energies and noise levels, we discarded the compound from all libraries if a fragmentation spectrum with, at most, one peak resulted for at least one collision energy and noise level. Doing so, 3,314 compounds were removed from the COSMIC training dataset, and 171 compounds were removed from the independent dataset. Substantially more compounds were removed from the COSMIC training dataset because many training dataset spectra have only few peaks, increasing chances that noisy spectra contain, at most, one peak. Here, 10-eV noisy spectra contain, at most, one peak for 75% of the 3,314 removed compounds; 20-eV noisy spectra for 27%; and 40-eV noisy spectra for 11% (a compound can exhibit sparse spectra for more than one collision energy).

This resulted in eight libraries: four libraries with 4,046 compounds each for the COSMIC training dataset and four libraries with 3,291 compounds each for the independent dataset. Notably, the COSMIC training dataset is a proper subset of the CSI training dataset; if we simply refer to ‘training data’ throughout this manuscript, this refers to the full CSI training dataset and includes the COSMIC training dataset. Recall that the COSMIC training dataset contains Orbitrap MS/MS data only, whereas the independent dataset contains QTOF MS/MS data only.

### Biological datasets and data processing

- For the mice fecal dataset, we analyzed LC–MS/MS data of 278 samples from a public metabolomics dataset (MassIVE data repository, MSV000082973). This dataset comes from a previously published study^69^. LC–MS/MS experiments were conducted on a Q Exactive Orbitrap instrument (Thermo Fisher Scientific). In brief, the fecal mice metabolome was analyzed by untargeted metabolomics from fecal pellet aqueous–methanol (1:1) extracts from specimens of an atherosclerosis mouse model (*Mus musculus* atherosclerosis-ApoE^−^^/−^). Specimens were either exposed or not exposed to intermittent hypoxia or hypercapnia (IHH). In addition, two groups were fed with an HFD or a regular diet; each group consists of 28 specimens.
- For the human dataset, we analyzed ten MassIVE datasets from the MassIVE data repository (MSV000083559, MSV000079651, MSV000080167, MSV000080469, MSV000080533, MSV000080627, MSV000081351, MSV000082261, MSV000082629 and MSV000082630). The dataset contains fecal, plasma, urine, lips, tongue and teeth samples from humans, all acquired on Q Exactive Orbitrap instruments (Thermo Fisher Scientific) in positive ion mode. Runs were acquired using C18 reversed-phase ultra-high-performance liquid chromatography. Only files with extensions ‘.mzML’ or ‘.mzXML’ were considered, and LC–MS runs containing spectra in profiled mode were discarded. This resulted in 2,666 LC–MS/MS runs being processed.
- For the Orbitrap dataset, we followed the idea of ‘flipping the workflow’ and reanalyzing public data at a repository scale. We restricted ourselves to MassIVE datasets measured on a Q Exactive Orbitrap instrument (Thermo Fisher Scientific), as this instrument had the largest number of MassIVE datasets. We applied no other constraints with regard to analyzed organism and LC setup, resulting in 264 public MassIVE datasets (downloaded on 20 February 2020). MassIVE datasets containing only spectra in profiled or negative ion mode were discarded, leaving us with 123 MassIVE datasets. Sample types range from environmental to natural products and include biological samples from at least 30 different species, covering diverse genera and phyla. Only files with extensions ‘.mzML’ or ‘.mzXML’ were considered, and LC–MS/MS runs containing spectra in profiled or negative ion mode were discarded, leading to 17,414 LC–MS/MS runs being processed. See Supplementary Table 4 for a list of all MassIVE datasets.

SIRIUS 4 was used to process LC–MS/MS runs and MassIVE datasets provided in mzML or mzXML format. Feature detection in SIRIUS 4 is similar in spirit to a targeted analysis. Instead of searching for all features in a run, SIRIUS first collects all fragmentation spectra and their precursor information and then searches for features that are associated with those fragmentation spectra (precursor ions, adduct ions and isotope peaks). Adducts and isotopes were detected using predefined lists of mass differences. Fragmentation spectra assigned to the same feature (precursor ion) are merged using an agglomerative clustering algorithm based on cosine distance. Compounds with mass beyond 700 Da were discarded to avoid high running time. MassIVE datasets that exceeded 600 LC–MS/MS runs were split to reduce memory consumption.

We use both isotope patterns and fragmentation patterns to determine the molecular formula de novo using SIRIUS 4 with default parameters and mass accuracy of 10 ppm. CSI:FingerID with default parameters was used to rank structure candidates. We use SIRIUS default soft thresholding of molecular formulas when querying CSI:FingerID structure candidates. For confidence score computation, we restrict the candidate list to those candidates with the same molecular formula as the highest-scoring candidate (hit). We used the highest-scoring structure candidate and the corresponding fragmentation tree, isotope pattern and structure candidate list features for COSMIC.

For the mice fecal dataset, SIRIUS results were imported into GNPS, and data were further annotated and explored by performing feature-based molecular networking and spectral library search on GNPS. The statistical and fold change analysis was performed using MetaboAnalyst 4.0 (ref. ^70^) for samples from control mice (not exposed to IHH) that were fed either an HFD or a regular diet.

### ROC characteristics and hop plots

We are given a list of hits, one for each query, ordered by score. Each hit can either be positive (correct annotation) or negative (incorrect annotation). Varying a score threshold, we can modify the number of hits reported to the user; our goal is to report all positives and to reject all negatives. True positives (*TP*s) and false negatives (*FN*s) are positives (correct hits) that pass or do not pass the threshold; similarly, false positives (*FP*s) and true negatives (*TN*s) are incorrect hits that pass or do not pass the threshold. For any score threshold, we plot the true positive rate *TP*/(*TP* + *FN*) (ratio of reported correct hits among all correct hits) against the false positive rate *FP*/(*FP* + *TN*) (ratio of reported incorrect hits among all incorrect hits), resulting in a ROC plot. The AUC of the ROC curve is the integral of the ROC curve; the random score, corresponding to a random ordering of hits, reaches AUC 0.5. A method may reach AUC below 0.5, meaning that the hit score performs worse than random. Different from binary classification, we must not invert ‘predictions’ to reach a better AUC. Logic dictates that the directionality of the hit score (such as, ‘high scores are good’) is fixed by the candidate identification task. The AUC measure makes no difference between the (highly relevant) lower-left and the (mostly irrelevant) upper-right of the ROC curve.

In contrast to binary classification, two methods can differ in the number of positives (correct hits, correct annotations) that they reach for the complete list of queries. This is a peculiarity of the identification task and has no equivalent in binary classifier evaluation, where the number of positives and negatives is determined by the dataset. ROC curves do not asses the number of positives; in particular, two methods can have identical ROC curves, although one method reaches twice as many correct hits. We introduce hop plots (inspired by the hop plant *Humulus lupulus* ranking to a supporting wire) to integrate this information. We again vary the score threshold but normalize reported correct hits and incorrect hits by the total number of hits (queries) *N* = *TP* + *FN* + *TN* + *FP*, plotting *TP*/*N* versus *FP*/*N* (Extended Data Fig. 1). The resulting curve starts in the origin (0,0) and ends in some point (*x*, *y*) ∈ [0,1]^2^ with *x* + *y* = 1, where *y* is the ratio of correct hits for the complete list of queries. The hop curve lies in the lower-left triangle; random ordering of hits corresponds to a straight line from the origin to some point (*x*, *y*) with *x* + *y* = 1. For perfect results, the hop curve is a straight line between the origin and (0,1); in the worst case, it is a straight line from the origin to (1,0). Hop plots allow us to answer questions such as, ‘If I fix some FDR, how many true discoveries will a method return?’ We stress that, to draw a ROC curve or a hop plot, we must have complete information about true and false positives and negatives, so we can calculate the exact FDR as *FP*/(*FP* + *TP*). A zoom-in allows us to compare methods in the particularly interesting region close to the origin. Both ROC curves and hop plots allow us to visually compare the performance of a method for different datasets in one plot; here, the total number of hits *N* is different for each curve.

Besides ROC curves, precision recall curves are frequently used to asses the performance of a binary classifier. Similarly to ROC curves, precision recall curves are not appropriate for the identification task, because ‘recall’ is normalized to the number of correct identifications, which is usually different for two methods. As ‘precision’ equals one minus FDR, ‘precision’ can directly be read from a hop plot, too.

We can calculate the AUC of a hop plot by mirroring the curve at the line *x* + *y* = 1 before taking the integral. A method with identification rate *y* ∈ [0,1] for the complete list of queries will have AUC between *y*^2^ and *y*^2^ + 2(1 − *y*)*y* = 1 − (1−*y*)^2^, with random ordering reaching area *y*^2^ + (1 − *y*)*y* = *y*. But, much like the AUC of a ROC curve, this number does not tell us whether a method performs well at the (highly relevant) lower-left or the (mostly irrelevant) upper-right of the curve; hence, we refrain from reporting hop plot AUC.

### Training CSI:FingerID and structure–disjoint evaluation

We trained an array of SVMs for fingerprint prediction from MS/MS data as described in refs. ^15,33,56^. Training of CSI:FingerID was carried out using merged spectra with all available collision energies from the CSI training dataset. In contrast, single collision energy and merged spectra libraries, as well as noisified spectra, were not used when training CSI:FingerID but only in validation of COSMIC. We used PubChem-standardized structures^71^ when computing the molecular fingerprint of a compound. In evaluations, we used the CSI:FingerID ‘covariance score’ from ref. ^72^ to rank candidates, comparing the probabilistic query fingerprint and each structure candidate fingerprint. A hit was regarded as correct if the PubChem-standardized structures of query and top rank were identical.

As noted above, all evaluations were carried out structure–disjoint. For the ten-fold cross-validation, we partitioned the training data into ten disjoint batches of almost identical size, ensuring that all fragmentation spectra from compounds with identical structure (such as L-threose and D-erythrose) end up in the same batch. Otherwise, L-threose could be part of the training data when evaluating on D-erythrose and vice versa. For each batch, we trained the fingerprint SVM array using the remaining nine batches; we evaluated on the tenth batch. In this way, we ensured that all compounds are novel for CSI:FingerID. For each query, MS/MS training data for the corresponding structure, including independent MS/MS measurements, were not available for CSI:FingerID.

CSI:FingerID evaluations on the independent dataset were again executed structure–disjoint. We additionally trained an SVM array using the complete CSI training dataset. Given an MS/MS query from the independent data, we checked if the structure of the query is also part of the training data. If so, we used the appropriate SVM array from cross-validation for fingerprint prediction; otherwise, we used the SVM array trained on the complete training data. Again, this ensured that all structures were novel in evaluation.

### Score calibration and *E*-value estimation

The *P* value of a score is the probability that a score this high or higher would be expected by chance; the *E* value is the expected number of random hits with this score or higher. Kim et al.^73^ suggested to use *E* values for peptide database searching; MS-GF *E*-value computation uses dynamic programming, based on the linear nature of peptides. Keich et al.^29^ calibrated peptide database search scores using decoys. Both approaches are conceptually hard to adopt for metabolite annotation. Metabolites have highly non-linear structure, and no methods have been suggested to generate reasonable decoy molecular structures for small molecules^12^.

We suggest using the distribution of scores of PubChem^30^ candidates as a proxy for the score distribution of incorrect hits. We empirically established that scores of an individual MS/MS query roughly followed a log-normal distribution; for other queries, the score distribution was multimodal (Supplementary Fig. 3). In particular, a small fraction of candidates had a much higher score than expected from the single log-normal distribution; ignoring this would result in inflated calibrated scores.

The log-normal distribution is a reasonable proxy if there are only few samples available. To model multimodal distributions as well as distributions that deviate from the log-normal distribution, we suggest using a kernel density estimate of the probability density function. Clearly, we do not have to ‘compute’ the kernel density; instead, we want to know the *E* value under the resulting distribution. For the ease of presentation, we do not use log-normal kernel functions but, instead, model the log-transform of the scores by normal kernel functions, which is mathematically equivalent. Let ${y}_{i}:={{\mathrm{ln}}}\,{x}_{i}$ for *i* = 1, …, *n* be the log-scores of the PubChem ‘proxy decoys’ excluding the hit score, and let $y:={{\mathrm{ln}}}\,x$ be the log-score of the hit. We first determine the bandwidth of the kernel function; we use Silverman’s rule of thumb, first determining the standard deviation $\hat{\sigma }$ of the sample *y*_1_, …, *y*_*n*_ and then setting

$$h:=1.059223841\times \hat{\sigma }{n}^{-1/5}.$$

We also tested other bandwidth estimation procedures but did not find a substantial difference (data not shown). For the Gaussian kernel $K(u):=\frac{1}{\sqrt{2\pi }}\exp (-\frac{1}{2}{u}^{2})$, we reach

$$K\left(\frac{y-{y}_{i}}{h}\right)=\frac{1}{\sqrt{2\pi }}\exp \left(-\frac{{(y-{y}_{i})}^{2}}{2{h}^{2}}\right)$$

so this is just the usual probability density function of the normal distribution times *h*, which cancels out in the kernel estimator. We calculate

1 $$\,{E}\,=\frac{m}{n}\times \mathop{\sum}\limits_{i=1,\ldots ,n}\left[\frac{1}{2}-\frac{1}{2}{{\mathrm{erf}}}\,\left(\frac{y-{y}_{i}}{\sqrt{2}h}\right)\right]$$

where *m* is the number of candidates in the biomolecule structure database.

### Confidence score computation

Our method of confidence estimation is inspired by the Percolator method for peptide identification in shotgun proteomics^74,75^. Different from there but similar to refs. ^76,77^, we do not train a classifier for an individual LC–MS run to ‘boost’ annotations; instead, we train classifiers only once using the reference measurements, which are then applied to the biological data. As noted by Käll et al.^74^, this approach is highly prone to overfitting. Characteristics of correct and incorrect hits might vary among experiments, instrument types, compounds present in the sample and others. Here, we have taken extensive measures to counter overfitting, such as ‘noisifying’ spectra and the restriction to linear SVMs.

We repeated the following for each collision energy (10 eV, 20 eV, 40 eV and merged spectra) and trained individual SVMs using spectra without added noise from that energy as training data. Features of the linear SVMs are shown in Supplementary Table 1. All features were individually standardized. Parameter *C* ∈ {10^−5^, 10^−4^, . . . , 10^5^} of each SVM was chosen by a nested cross-validation. We used quadratic hinge loss and *l*_2_ regularization. SVMs were trained using LIBLINEAR^78^.

For each collision energy, we trained three classifiers. (1) When searching PubChem, we used all appropriate features (all but Features 20–22) from Supplementary Table 1. Searching the biomolecule structure database, not all queries result in two or more candidates; but some features from Supplementary Table 1 require a candidate list of at least size two, such as the difference between score of highest-scoring versus runner-up candidate. To this end, we trained two classifiers for the biomolecule structure database. (2) The regular SVM assumes that there are at least two candidates; it uses all features from Supplementary Table 1 but is trained only on the appropriate subset of the training data. (3) The single-candidate SVM uses only the appropriate sub-features (all but Features 1–4, 10 and 13) but can be trained using all training data. For instances with two or more candidates, we uniformly selected one candidate.

The resulting linear classifiers showed clear signs of overfitting. For example, some features received weights that were counterintuitive, such as negative weight for the quality of the SIRIUS fragmentation tree or the CSI:FingerID score. Recall that the actual hit was chosen by CSI:FingerID as the candidate with the highest score; hence, logic dictates that the CSI:FingerID score of the hit must not receive a negative weight when deciding whether a hit is correct or incorrect. The same is true for selecting the best fragmentation tree by SIRIUS. To this end, we enforced directionality of the features. For each feature, we decided manually whether a high value of the feature would increase or decrease our confidence in an annotation. For example, a high CSI:FingerID score should clearly increase our confidence and so should a small *E* value. See Supplementary Table 1 for enforced directions. Notably, enforcing directionality can be achieved by a regular SVM optimization without additional constraints, allowing us to use established SVM solvers. For each feature with enforced directionality, we augmented one training sample where the corresponding feature was set to a large (positive or negative) value ± *β*, whereas all other features were kept at zero; the sample received a positive label (correct hit). If the absolute feature value *β* > 0 is large enough, then an optimal solution must use the feature in the desired direction; the actual value *β* is of minor importance due to the hinge loss of SVM optimization. To avoid potential numerical instabilities when finding the solution, *β* should not be chosen too large. Here, we used *β* = 10^7^; using absolute feature values 10^8^ and 10^9^ resulted in basically identical models, and differences are of no practical consequence (data not shown). Notably, some features received non-zero weights for the classifier with enforced directionality, despite the fact that these features received ‘counter-intuitive’ weights in the unrestricted optimization. For example, feature ‘FP Length Hit’ was repeatedly given negative weight in cross-validation but had high positive weight if we enforced directionality (unrestricted weight − 0.00165, restricted weight 0.0568 in the same cross-validation fold).

When training the COSMIC SVMs, all CSI:FingerID fingerprint predictions of training spectra were carried out structure–disjoint using CSI:FingerID cross-validation models. The COSMIC training dataset was then partitioned for ten-fold cross-validation in the same fashion as for CSI:FingerID training. Hence, cross-validation evaluation of COSMIC is again structure–disjoint, and all compounds are novel. Similarly to above, we also ensured structure–disjoint evaluations on the independent dataset by choosing the appropriate SVM from cross-validation for computing the confidence score. When applying the model to independent data, we capped feature values. For each feature from Supplementary Table 1, we record the minimum and maximum feature value in our training data. When applying the model, feature values exceeding these thresholds are set to the respective threshold value. We do so to prevent exaggerated decision values caused by unexpectedly high values of one or more features.

We map decision values to posterior probability estimates using Platt probabilities^31^. Platt^31^ proposed to use a sigmoid function as an approximation of posterior probabilities: ${\mathbb{P}}(y=\,{{\mbox{correct}}}\,| x)\approx {P}_{A,B}(f)\equiv \frac{1}{1+\exp (Af+B)}$, where $f=f(x)\in {\mathbb{R}}$ is the decision value for hit *x* and *y* ∈ {correct, incorrect} is its label. We estimated parameters $A,B\in {\mathbb{R}}$ using maximum likelihood^31,79^ as implemented in LIBSVM^80^.

Using a linear classifier enables explainable machine learning; see Supplementary Table 1 for feature weights after normalization of the three classifiers for merged spectra. We observe that certain features have weight close to zero; this might indicate that the feature is indeed uninformative, that the feature does not measure what we intended to measure or that our training data are insufficient to learn a reasonable weight.

Recall that confidence SVMs were trained exclusively on spectra without added noise. We also trained SVMs from a combined dataset with all noise levels but found that results were of identical quality when applied to the same evaluation dataset (data not shown).

Unlike Percolator^74,75^, we do not learn a confidence score for individual LC–MS datasets. We do so because it is non-trivial to generate reasonable decoys for small molecules and, more importantly, because incorrect hits in the target database are often not random (Fig. 4)^35^. This potentially explains why the calibrated *E*-value score presented here does not allow for a satisfactory separation. Also unlike Percolator, we do not use our scores to re-rank candidates^74,75^. All of our candidates share the same molecular formula, fragmentation tree and predicted fingerprint; these features are meaningless for re-ranking. To this end, curves of CSI:FingerID and COSMIC in hop plots (Figs. 2 and 5) always end in the same point (*x*,*y*) with *x* + *y* = 1.

In application, a model with the exact collision energy of the experimental measurement might not be available; in this case, the model with the smallest difference in collision energy (such as the 40-eV model for 35 eV collision energy) is chosen by COSMIC.

### FDR estimation

Recall that the FDR equals *FP*/(*FP* + *TP*) where *TP* is the number of true positives (correct hits above some score threshold) and *FP* is the number of false positives (incorrect hits above the same score threshold). Also recall that, to compute this exact FDR, we must know the exact numbers *FP* and *TP*. However, in applications, we do not have this information; in this case, we need some method to estimate FDR values. Returning random numbers would be an admissible method for FDR estimation, albeit a useless one; to this end, a method for FDR estimation has to be validated against exact FDR values, to assess its accuracy. In application, a user selects an acceptable FDR level, and we want to return as many hits as possible so that the list of hits meets the pre-selected FDR. The *q* value of a hit is the smallest FDR at which this hit is part of the output list.

We now show how to transform COSMIC confidence scores to FDR estimates. The confidence score is an estimated posterior probability of the hit being correct; to this end, it is one minus the posterior error probability for this hit. Hence, we can use the confidence score to estimate the FDR of the top *k* hits^12,36^. Let *p*_*j*_ be the posterior error probability for hit *j* for *j* = 1, …, *n* and assume that the hits are ordered by confidence score, so *p*_*j*_ ≤ *p*_*j*+1_. Viewing the annotations as (not necessarily independent) Bernoulli trials, the expected number of incorrect annotations for the top *k* hits is $\mathop{\sum }\nolimits_{j = 1}^{k}{p}_{j}$, and the expected FDR is

2 $${\widehat{{FDR}}}_{k}=\frac{1}{k}\times \mathop{\sum }\limits_{j=1}^{k}{p}_{j}.$$

Because hits have been ordered by posterior error probability, FDR estimates ${\widehat{{FDR}}}_{k}$ are monotonically increasing, so ${\widehat{{FDR}}}_{k}$ is also the *q* value estimate for hit *k*.

We evaluate the accuracy of our FDR estimates by plotting exact *q* values against estimated *q* values in a Q–Q plot (Extended Data Fig. 5); this has to be carried out using reference data where exact FDR values can be calculated.

### Comparing molecular structures

The Tanimoto coefficient measures the similarity of two molecular structures. Any Tanimoto coefficient is based on a particular set of molecular properties, constituting the fingerprint type. For consistency, we use the same fingerprint type (molecular properties) throughout this manuscript that we have trained SVMs for as part of CSI:FingerID. The Tanimoto coefficient is the Jaccard index of the two sets of molecular properties—that is, the cardinality of the intersection of the two sets divided by the cardinality of the union of the two sets. The advantage of the Tanimoto coefficient is that it can be quickly calculated, in particular if we have pre-computed the fingerprints of all molecular structures of interest.

For highly similar molecular structures, such as the pairs in Fig. 4, it is not advisable to employ the Tanimoto coefficient, as it is not apt to accurately measure such high similarity. Instead, we represent the two molecular structures as graphs and ask for a minimum number of edges that have to be removed from the graphs, such that the resulting graphs are isomorphic; naturally, hydrogen atoms are ignored in this computation. This is the maximum common edge subgraph (MCES) problem. Using the number of removed edges to estimate dissimilarity is an appropriate measure for highly similar molecules, as we explicitly do not demand that the resulting subgraph is connected. The MCES problem is NP-complete, as it generalizes subgraph isomorphism. See, for example, ref. ^81^ for a discussion of available methods for solving MCES exactly and heuristically.

For the molecular structures in Fig. 4, it is straightforward to manually find optimal solutions. The ‘top hit’ structure can be transformed into the ‘correct hit’ structure via two edge deletions for examples **a**–**c** and **f**–**i**, whereas examples **d** and **e** require four edge deletions. Because both graphs have the same number of edges, we require at least two edge deletions for non-isomorphic graphs.

### CASMI 2016 re-evaluation

Scores of MetFrag, MAGMa+, CFM-ID, CSI:FingerID (original) and CSI:FingerID IOKR were downloaded from the CASMI 2016 results web page (http://casmi-contest.org/2016/, category 2, automated methods). We only consider tools that scored all candidates. CSI:FingerID (original) and CSI:FingerID IOKR were not executed structure–disjoint, as CASMI is a blinded competition. We computed scores for the structure–disjoint evaluation of CSI:FingerID using CSI:FingerID 1.2.0.

We used hit scores (score of the top-ranked candidate for each query) to order hits. For consistency, we restricted the set of candidate structures to those with the correct molecular formula for all tools. We performed evaluation either using all ChemSpider candidates or restricting the search to those ChemSpider candidates that are simultaneously found in our biomolecule structure database. In four cases, this resulted in an empty list of candidates, and these queries were excluded from evaluation. In 13 cases, the set of candidates did no longer contain the correct structure; these queries were not excluded from evaluation. As expected^82^, MetFrag, MAGMa+ and CFM-ID profit more from restricting the set of candidates than CSI:FingerID^15^; hence, annotation rates varied less than those reported in the CASMI evaluation^18^. In fact, even randomly choosing one of the remaining candidates resulted in a decent annotation rate when searching the biomolecule structure database. In 38 cases, only a single candidate remained; and, in 33 cases, the candidate list contained two or three structures. Even if there is only a single candidate, the score that some in silico tool assigns to this candidate is important information, as we use it to order hits.

The fact that scores of in silico tools, including CSI:FingerID, cannot be used to decently separate correct and incorrect hits might be unexpected for users, but tools and scores were not developed with this application in mind. To this end, our findings must not be misunderstood as a critique of these tools or their developers.

COSMIC confidence scores were computed as described above, using the confidence score model for ‘merged spectra’. We ensured structure–disjoint evaluation (all compounds novel) for both CSI:FingerID and COSMIC, as detailed above. For both ChemSpider and the biomolecule structure database, we used the confidence score variant for searching the biomolecule structure database; this is reasonable as the number of ChemSpider candidates is often substantially smaller than the number of PubChem candidates.

For completeness, we also evaluated separation of the original submissions of CSI:FingerID and CSI:FingerID IOKR (Supplementary Fig. 2). As noted, these evaluations were not carried out structure–disjoint; hence, results mix de-replication (structures for which MS/MS data are available in the training data) and novel structure search. We cannot compute confidence scores for the original CSI:FingerID submission, as features required for its computation (Supplementary Table 1) were not recorded when submitting the CASMI entry.

### In-depth method evaluation

For a query fragmentation spectrum, we again assume to know its molecular formula, and we obtained candidates from the structure databases using this molecular formula. In practice, molecular formulas can be established using SIRIUS 4 (ref. ^33^) or ZODIAC^83^. For 325 compounds in the COSMIC training dataset and 278 compounds in the independent data, this resulted in an empty candidate list when querying the biomolecule structure database; these compounds were excluded from evaluation, leaving us with 3,721 queries in cross-validation and 3,013 queries for independent data. For 845 compounds in the COSMIC training dataset and 521 compounds in the independent data, the correct structure is not present in the biomolecule structure database; these compounds were not excluded. We ensured structure–disjoint evaluation (all compounds novel) for both CSI:FingerID and COSMIC.

To evaluate against spectral library search, we generated two spectral libraries based on the CSI training dataset: one library with merged spectra and one library with spectra at individual collision energies as well as merged spectra. We searched merged query spectra in the first library and query spectra containing a single collision energy in the second library. Merged spectra are identical to those used for training CSI:FingerID (see above); this library contains 23,965 spectra. The second library contains all available fragmentation spectra at all available collision energies, plus the merged spectra, and contains 189,979 spectra. Notably, the spectral library contains MS/MS data from QTOF and Orbitrap instruments, whereas all query MS/MS spectra are QTOF data. We argue that this resembles how searching in a public or commercial spectral library is executed in practice. The situation is clearly different for an in-house spectral library, but such libraries are usually one to two orders of magnitude smaller. For 821 query compounds, the correct structure is not present in the spectral library; as for COSMIC, these compounds were not excluded from evaluation. To ensure a fair comparison with COSMIC, spectral library search candidates were restricted to those with the correct molecular formula for each query; in practice, this information is usually not available, and spectral library search might perform worse than reported here. In case the spectral library did not contain at least one candidate with the correct molecular formula of the query, a misannotation with score zero was assumed. We evaluated both the cosine score described above and a cosine score using the square root of intensities.

We also evaluated spectral library search when restricting library spectra to the ‘correct’ collision energy (closest energy from 15 eV to 25 eV for 20-eV queries) but found that both annotation rates and separation were substantially worse than for the combined library (data not shown).

The term ‘spectral library search’ refers to searching for a query fragmentation spectrum in a database of reference fragmentation spectra measured from (usually commercial) standards and then reporting the highest-scoring candidate (hit) under some scoring. Spectral library search must not be mistaken with the task of comparing mass spectra, manually or automated, or with computing a measure of similarity between spectra, such as the cosine score. Comparison of mass spectra is in use for many research questions beyond spectral library search. This includes the manual confirmation of annotations, MASST^28^, as well as CSI:FingerID (and, hence, COSMIC), which uses the cosine score as part of its machine learning framework.

### Sciex dataset evaluation

We queried the biological structure database using the positive ionization mode data. As in the other evaluations, we assumed that the correct molecular formula of each query was known. For 13 queries, this resulted in an empty candidate list; these instances were excluded from our evaluation. For the remaining 301 queries, the correct answer was not present in the biological structure database in four cases; these queries were not excluded. Because fragmentation spectra were recorded as ramp spectra, we used the ‘merged spectra’ confidence score model. We ensured structure–disjoint evaluation both for CSI:FingerID and COSMIC. Nineteen structures from the Sciex dataset were not present in the training data.

### Annotation, manual confirmation and validation of novel bile acid conjugates

For the mice fecal dataset, MS/MS measurements were taken with a collision energy of 30 eV; we used the COSMIC version trained on 40-eV spectra. The bile acid conjugates structure database was used for the annotation. No additional parameters have to be chosen in the COSMIC workflow.

The output of this workflow is an ordered list of 1,456 COSMIC structure annotations (‘MS features’; Supplementary Data 1). In case multiple compounds were annotated with the same structure (for example, compounds being present in multiple runs and different adducts of the same compound), entries in the COSMIC output were merged and represented by the hit with the highest confidence. This reduces the output to 626 unique structure annotations (Supplementary Table 2). Of these, 113 were present in PubChem. Here, we concentrated on the 513 ‘truly novel’ bile acid conjugates. The *q* value estimates reported in Fig. 6a were computed via eq. (2) using only the ‘truly novel’ bile acid conjugates.

The top 12 most confident bile acid conjugate annotations were manually inspected, and the fragmentation was interpreted to check consistency with the structure proposed by COSMIC (Supplementary Figs. 8–19). The fragmentation of bile acid conjugates is characterized by fragment ions and neutral losses from the conjugated amino acid moiety as well as the hydroxylation pattern of the bile acid core. Annotations of two ‘truly novel’ bile acid conjugates—phenylalanine (Phe) and tryptophan (Trp) conjugates of chenodeoxycholic acid (CDCA)—were verified by comparing their fragmentation spectra and retention times with those of synthetic standards. Phe-CDCA (7) and Trp-CDCA (12) were synthesized using a procedure adapted from a previous method by Ezawa et al.^84^. Chenodeoxycholic acid (98.1 mg, 0.25 mmol, 1 eq.) was dissolved in THF (4.9 ml, 0.05 M) and cooled to 0 ^°^C with stirring. Ethyl chloroformate (28 μl, 1.2 eq.) was added, followed by triethylamine (41 μl, 1.2 eq); then, the reaction was stirred for 2 h in an ice bath. After complete conversion of the starting material by TLC, a cold, aqueous solution (4.9 ml) of amino acid (0.37 mmol, 1.5 eq.) and NaOH (14.8 mg, 0.37 mmol, 1.5 eq.) was added in one portion. The reaction was then stirred for 2 h, gradually warming to room temperature. THF was removed in vacuo, and 2 M HCl was added to acidify to pH < 2, at which point a white precipitate appears. The mixture was extracted with ethyl acetate (3 × 20 ml), and the combined organic layers were washed with brine (1 × 50 ml), dried over sodium sulfate and concentrated. The crude material was purified over silica gel by column chromatography eluting with 3–10% methanol in dichloromethane (plus 1% acetic acid, vol/vol) to yield the desired products as confirmed by NMR spectroscopy. NMR spectra were recorded on a Bruker Avance (600 MHz, CryoProbe) spectrometer in CD_3_OD. Signals are reported in ppm with the internal CD_3_OD signal at 3.31 ppm (^1^H) and 49.0 ppm (^13^C) as standard reference peak.

- Phenylalanine-conjugated chenodeoxycholic acid (Phe-CDCA): Product was obtained as a white solid in 91% yield. ^1^H NMR (599 MHz, MeOD): 7.29–7.18 (m, 5H), 4.67–4.61 (m, 1H), 3.81–3.78 (m, 1H), 3.42–3.33 (m, 1H), 3.22 (dd, *J* = 14.4, 4.8 Hz, 1H), 2.93 (dd, *J* = 13.8, 9.0 Hz, 1H), 2.27 (q, *J* = 12.0 Hz, 1H), 2.22–2.17 (m, 1H), 2.10–2.03 (m, 1H), 2.01–1.94 (m, 2H), 1.90–1.81 (m, 3H), 1.77–1.57 (m, 4H), 1.54–1.45 (m, 4H), 1.41–1.26 (m, 5H), 1.24–1.04 (m, 5H), 1.03–0.95 (m, 1H), 0.93–0.86 (m, 7H). ^13^C (151 MHz, MeOD): 175.22, 137.28, 128.88, 128.00, 126.32, 71.47, 67.66, 55.94, 50.13, 42.26, 41.78, 39.65, 39.37, 39.08, 37.06, 35.44, 35.17, 34.83, 34.51, 32.65, 32.45, 31.82, 29.96, 27.84, 23.23, 22.02, 20.39, 17.48, 10.81.
- Tryptophan-conjugated chenodeoxycholic acid (Trp-CDCA): Product was obtained as a white solid in 42% yield. ^1^H NMR (599 MHz, MeOD): 7.56 (d, *J* = 7.8 Hz, 1H), 7.33 (d, *J* = 7.8 Hz, 1H), 7.10–7.06 (m, 2H), 7.00 (t, *J* = 7.8 Hz, 1H), 4.73 (dd, *J* = 8.4, 4.8 Hz, 1H), 3.81–3.77 (m,1H), 3.41–3.32 (m, 2H), 3.18–3.13 (m, 1H), 2.31–2.16 (m, 2H), 2.10–2.03 (m, 1H), 1.98–1.93 (m, 2H), 1.88–1.78 (m,3H), 1.73–1.63 (m, 3H), 1.63–1.58 (m, 1H), 1.54–1.43 (m, 5H), 1.41–1.25 (m, 5H), 1.24–0.94 (m, 7H), 0.91(s, 3H), 0.89 (d, *J* = 7.2 Hz, 3H), 0.63 (s, 3H). ^13^C (151 MHz, MeOD): 176.65, 175.39, 138.01, 128.87, 124.24, 122.37, 119.78, 112.28, 111.12, 72.84, 69.07, 57.22, 54.61, 51.49, 43.62, 43.14, 41.00, 40.72, 40.45, 36.78, 36.53, 36.20, 35.89, 34.02, 33.78, 32.99, 31.33, 29.16, 28.45, 24.60, 23.39, 21.76, 18.86, 12.15.

Reference standards were analyzed by LC–MS/MS using identical experimental conditions as used previously^85^. Retention times were 298.5 s for Phe-CDCA and 294.5 s for Trp-CDCA. Samples from the previous study were re-analyzed to ensure comparability of retention times: the putative Phe-CDCA and Trp-CDCA candidates had retention times of 300.5 s and 294.5 s, respectively. Considering the similarity of their fragmentation spectra (Extended Data Fig. 8) and retention times, these are MSI level 1 identifications. However, these identifications are not unambiguous: isomeric structures, such as Phe-deoxycholic acid, would show the same fragmentation spectrum and the same or very similar retention time. For a conclusive decision, a more detailed analysis method would be required, which is out of the scope of this paper.

Molecular networks were visualized in Cytoscape (v3.7.1)^86^. The MetaboAnalyst web server^70^ was used to process the feature quantification results and perform statistical analysis in Fig. 6c. Quantile normalization and auto-scaling were used. Results of the fold change analysis were mapped onto molecular networks using Cytoscape. Primary (cholic acid, ursocholic acid and muricholic acid) and secondary (deoxycholic acid, hyodeoxycholic acid and chenodeoxycholic acid) bile acids and historically known bile acid conjugates (glycocholic acid and taurocholic acid) were annotated by spectral library search. Similarly, the three bile acid conjugates from ref. ^40^ (tyrosocholic acid, phenylalanocholic acid and leucocholic acid) were annotated by spectral library search. For visualizing the relative feature intensity and ratio (Supplementary Figs. 22–28), box plots were generated directly from the feature quantification results (no normalization and scaling applied). We chose taurocholic acid, deoxycholic acid and hyodeoxycholic acid to compute ratios in Supplementary Figs. 24–26 as these are highly abundant in rat bile and rat feces^87^. MASST^28^ was used to search the annotated bile acid conjugates spectra in all public mass spectrometry datasets, including MassIVE-GNPS^37^, MetaboLights^4^ and Metabolomics Workbench^5^. Parameters and results for these jobs are part of Supplementary Table 2.

### Repository-scale annotation of novel metabolites

To estimate a reasonable COSMIC confidence score cutoff, we made use of our reference data evaluation results. In our evaluation using independent data, collision energy of 20 eV and medium noise, a confidence score threshold of 0.64 corresponded to FDR 10%. Our implicit assumption is that, for the biological data, this threshold will correspond to a similar FDR. It must be understood that we cannot guarantee a similar FDR for structure annotations below, given our inability to accurately estimate FDR. Clearly, many hits with confidence below this threshold will nevertheless be correct.

We searched the human dataset against the biomolecule structure database; this resulted in 114,012 hits. Multiple hits can annotate the same structure; for example, these hits might originate from different LC–MS/MS runs or different adducts. Hence, we report unique structures instead, where the hit with the highest confidence is used as a representative for that structure. This resulted in 24,554 unique structures being annotated, of which 3,167 (12.9%) were present in the CSI training dataset. We now filter the 24,554 structure annotations for high confidence (score threshold 0.64), resulting in 911 structure annotations. Of these high-confidence annotations, 475 (52.1%) were present in the CSI training dataset, leaving us with 436 (47.9%) high-confidence novel structure annotations. Finally, we excluded all hits with structures in the HMDB structure database, resulting in 21,128 unique structure annotations, 436 high-confidence structure annotations and 315 high-confidence structure annotations without reference MS/MS data (Fig. 10). Of the 315 novel structures, 48 were proteinogenic peptides.

We searched 14 character InChI keys of all 267 novel metabolite structures in the current version of the HMDB (February 2021) and found that at least 23 of these structures are present in the current HMDB version. The exact number might be slightly higher, as structures from the current HMDB version were not standardized using the PubChem standardization procedure. Notably, the recent inclusion of structures in the HMDB does not mean that reference MS/MS data are available for these structures.

High-confidence hits were manually evaluated by checking spectra for known neutral losses and fragments that can be explained. Furthermore, spectra were compared against reference spectra from similar structures. The following paragraphs discuss some high-confidence annotations where evaluation based on manual interpretation or newly generated reference spectra was possible. For none of the structures verified by spectral comparison ((2E)-octenoyl-carnitine, N-oleyl-leucine, phenazine-1,6-dicarboxylic acid) were reference spectra available in the training data of COSMIC or CSI:FingerID.

First, acyl-carnitine structures were evaluated by their typical fragmentation. Characteristic fragments are found at *m/z* 85 and *m/z* 144. These are derived from an ene-type loss of the neutral fatty acid yielding *m/z* 144, undergoing a further loss of trimethylamine to yield *m/z* 85. The same loss of trimethylamine can occur from the intact molecule, yielding a fragment found at a neutral loss of 59 Da. Based on this fragmentation pattern, eight high-confidence hits were ruled out and are presumably incorrect annotations. Of these eight bogus annotations, three potentially have the wrong adduct annotation. Based on our manual verification, 21 of 30 annotations of the acyl-carnitines are correct. Furthermore, the query spectrum annotated as (2E)-octenoyl-carnitine showed good agreement with a reference measurement (Supplementary Fig. 29).

Second, several N-acyl-amino acids were manually confirmed. Fragmentation of [M+H]^+^ adducts of N-acyl amino acids typically yields an ene-type of loss of a neutral fatty amide or the neutral loss of a fatty acyl ketene structure. Additional fragmentation yields typical amino acid fragments, allowing to potentially identify the amino acid in more detail. Within the human dataset, N-oleyl-leucine was annotated with a high confidence score. For this structure, reference spectra are now available in MassBank^68^. A high spectral similarity (cosine score 0.85) was found between the spectrum and the reference (Supplementary Fig. 29). Because MS cannot differentiate between isomeric species, the structure might also represent N-oleyl-isoleucine: spectra of N-oleyl-leucine and N-oleyl-isoleucine are both present in MassBank but are indistinguishable. Another example is N-palmitoyl-tryptophan. No reference spectrum is available for this substance, but the observed fragmentation pattern is in good agreement with expected fragmentation, showing *m/z* 205, which relates to tryptophan based on the loss of palmitic acid as ketene, and *m/z* 188, which is related to the loss of palmitic acid as neutral amide. Additional fragments are typically observed in the fragmentation of tryptophan. Using MASST, 12 additional human datasets containing a similar spectrum were identified.

Third, phenazine-1,6-dicarboxylic acid was annotated in a human urine dataset. This metabolite is produced by *Streptomyces* and *Pseudomonas* species^88^, hinting at a potential urinary tract infection. Again, the query spectrum showed good agreement with a reference measurement (Supplementary Fig. 29).

Compound classes in Extended Data Fig. 10 were assigned by NPClassifier^89^. Proteinogenic amino acids and peptides were selected manually. We installed a web interface allowing interested users to browse through structure annotations ordered by confidence, check spectra, access underlying datasets, leave comments and judge the overall quality of the annotation for the human dataset. The web interface is available at https://bio.informatik.uni-jena.de/cosmic.

To demonstrate that COSMIC can be applied at a repository scale, we searched the Orbitrap dataset with 17,414 LC–MS/MS runs against the biomolecule structure database; this resulted in 979,521 hits. Again, multiple hits can annotate the same structure; the above hits correspond to 77,932 unique annotated structures, of which 8,172 (10.5%) were present in the CSI training dataset. We now filter the 77,932 structure annotations for high confidence (score threshold 0.64), resulting in 3,530 structure annotations. Of these high-confidence structure annotations, 1,815 (51.4%) were present in the CSI training dataset, leaving 1,715 (48.6%) high-confidence novel structure annotations (Supplementary Fig. 30). Again, all hits of the Orbitrap dataset can be accessed via a web interface available at https://bio.informatik.uni-jena.de/cosmic.

The above computations were carried out on a compute node with 2 × 48 cores, AMD EPYC 7642 processors and 1 TB RAM. For running times on a laptop computer, we selected a ‘typical’ MassIVE dataset with 44 LC–MS/MS runs (MSV000080553, rosemary samples). We analyzed the data on a common laptop computer (Quad-Core Intel CPU i7-7700HQ, 16 GB RAM). SIRIUS default parameters were used; in particular, fragmentation tree computation (which is the most time-demanding step of the computational analysis) was done exactly for compounds below 350 Da and in hybrid mode above 350 Da. After feature alignment, 1,961 putative compounds with mass 125–968 Da were detected. We restricted the analysis to compounds below 700 Da, resulting in 1,854 compounds to be processed. Overall wall clock running time was 149 min, 59 s. We note that running time is dominated by the number of compounds in a dataset.

### Reporting Summary

Further information on research design is available in the Nature Research Reporting Summary linked to this article.

## Online content

Any methods, additional references, Nature Research reporting summaries, source data, extended data, supplementary information, acknowledgements, peer review information; details of author contributions and competing interests; and statements of data and code availability are available at 10.1038/s41587-021-01045-9.

## Supplementary information

Supplementary Information Supplementary Figs. 1–30 and Supplementary Tables 1 and 4

Reporting Summary

Supplementary Table 2 626 unique structure annotations for bile acid conjugates

Supplementary Table 3 315 unique structure annotations not present in the HMDB annotated in the human dataset

Supplementary Table 5 1,715 novel unique structure annotations from the Orbitrap dataset

## Data Availability

The Sciex dataset has been deposited on GNPS, accession numbers CCMSLIB00006581625 to CCMSLIB00006581938. Input mzML/mzXML files are available at MassIVE (https://massive.ucsd.edu/) with accession numbers MSV000082973 (mice fecal dataset); MSV000084630 (mass spectrometry analysis of the synthetic standards for Phe-CDCA and Trp-CDCA); and MSV000083559, MSV000079651, MSV000080167, MSV000080469, MSV000080533, MSV000080627, MSV000081351, MSV000082261, MSV000082629 and MSV000082630 (human dataset). See Supplementary Table 4 for accession numbers of the Orbitrap dataset. Metadata for synthetic standards of Phe-CDCA and Trp-CDCA were deposited with the dataset MSV000084630. Fragmentation spectra of Phe-CDCA and Trp-CDCA were deposited on GNPS (CCMSLIB00005467952 and CCMSLIB00005716808). Fragmentation spectra of all other manually confirmed bile acid conjugates were also deposited on GNPS; see Supplementary Table 2 for individual spectra IDs. Fragmentation spectra of N-oleyl-leucine (RP029701 ([M+H]^+^, 10 eV), RP029702 ([M+H]^+^, 20 eV) and RP029703 ([M+H]^+^, 40 eV)) and phenazine-1,6-dicarboxylic acid (RP018701 ([M+H]^+^, 10 eV), RP018702 ([M+H]^+^, 20 eV) and RP018703 ([M+H]^+^, 40 eV)) are available from MassBank. The fragmentation spectrum of (2E)-octenoyl-carnitine was deposited on GNPS (CCMSLIB00006581932). Parameters and results of LC–MS/MS processing for the mice fecal dataset are available at https://gnps.ucsd.edu/ProteoSAFe/status.jsp?task=e78a8c8f429a46fcb24f3b34d69aff25. The bile acid conjugate structure database is available at https://github.com/lfnothias/Combinatorial_BileAcids_DB_COSMIC. Spectral libraries generated from the high-confidence COSMIC annotations of the mice fecal, human and Orbitrap datasets are available at https://bio.informatik.uni-jena.de/cosmic/. The biomolecule structure database is a union of the following structure databases: HMDB (http://www.hmdb.ca), KNApSAcK (http://kanaya.naist.jp/knapsack), ChEBI (https://www.ebi.ac.uk/chebi/), KEGG (http://www.kegg.jp/), HSDB (https://toxnet.nlm.nih.gov/cgi-bin/sis/htmlgen?HSDB), MACONDA (http://www.maconda.bham.ac.uk), BIOCYC (http://biocyc.org/), structures from GNPS (https://gnps.ucsd.edu), biological subset of ZINC (http://zinc.docking.org), structures from MassBank (http://www.massbank.jp), UNDP (http://pkuxxj.pku.edu.cn/UNPD), PLANTCYC (http://pmn.plantcyc.org/) and YMDB (http://www.ymdb.ca/compounds/YMDB). SMILES of all structures in the biomolecule structure database can be downloaded from https://bio.informatik.uni-jena.de/cosmic/. The PubChem structure database is available for download from https://ftp.ncbi.nlm.nih.gov/pubchem/Compound/ (16 January 2019). Source data are provided with this paper.

## Source data

Source Data Fig. 2 Statistical Source Data
Source Data Fig. 3 Statistical Source Data
Source Data Fig. 5 Statistical Source Data
Source Data Fig. 6 Statistical Source Data
Source Data Extended Data Fig. 1 Statistical Source Data
Source Data Extended Data Fig. 2 Statistical Source Data
Source Data Extended Data Fig. 5 Statistical Source Data
Source Data Extended Data Fig. 6 Statistical Source Data
Source Data Extended Data Fig. 9 Statistical Source Data

## Code availability

COSMIC is written in Java and is integrated into the current release version of SIRIUS 4. It is open source under the GNU General Public License (v3) and is available for Windows, macOS X and Linux operating systems. We also provide source code, executable binaries, living documentation, training videos and sample data, as well as the public part of the training data, on the SIRIUS website (https://bio.informatik.uni-jena.de/sirius/). A source copy is hosted on GitHub (https://github.com/boecker-lab/sirius/).

Scripts for generating the bile acid conjugate structure database are available at https://github.com/lfnothias/Combinatorial_BileAcids_DB_COSMIC.
