# Supplementary material for: High-confidence structural annotation of metabolites absent from spectral libraries
Source: Nat Biotechnol. 2021 Oct 14;40(3):411–21. doi: 10.1038/s41587-021-01045-9 (PMC8926923; doi:10.1038/s41587-021-01045-9)
Supplement: Supplementary file 1 — Supplementary Figs. 1–30 and Supplementary Tables 1 and 4 [file 41587_2021_1045_MOESM1_ESM.pdf]

nature portfolio

https://doi.org/10.1038/s41587-021-01045-9

Supplementary information

High-confidence structural annotation of metabolites absent from spectral libraries

In the format provided by the authors and unedited

Supplementary Table 1: Features of the COSMIC confidence score and classifier weights for merged spectra. Features 1–19 are used for PubChem and biomolecule structure confidence scores, features 20–22 are exclusive to the biomolecule structure confidence scores. For the linear SVM trained using merged spectra (all collision energies), we provide classifier weights for PubChem ("PC"), biomolecular structure database with one candidate ("bio_1") and two or more candidates ("bio_{2+}"). Clearly, features that require at least two candidates cannot be used for classifier "bio_1". *Unless explicitly stated otherwise*, we consider the candidate list from PubChem for the PubChem classifier, and the candidate list from the biomolecular structure database for the biomolecular structure classifier. CSI:FingerID scores are Modified Platt score from Dührkop *et al.* (*Proc Natl Acad Sci USA* 112, 12580–12585, 2015) and Covariance score from Ludwig *et al.* (*Bioinformatics* 34, i333–i340, 2018). Multiple structures in the candidate list represented by the same fingerprint were treated as a single entry. Column 'Δ' shows if we enforced a feature to have positive ('P') or negative ('N') weight in the classifier. Features are individually normalized.

		Classifier weights				
#	Δ	bio_{2+}	bio_1	PC	Name	Description
1	P	0.1452		0.1167	Log Score Diff. 1	Difference between log scores of highest-scoring vs. runner-up candidate, Modified Platt score
2	P	0.0000		0.0386	Score Diff. 1	Difference between scores of highest-scoring vs. runner-up candidate, Modified Platt score
3	P	0.1104		0.1167	Log Score Diff. 2	Difference between log scores of highest-scoring vs. runner-up candidate, Covariance score
4	P	0.0013		0.0316	Score Diff. 2	Difference between scores of highest-scoring vs. runner-up candidate, Covariance score
5	P	0.0861	0.0424	0.0340	Modified Platt Score	Modified Platt score of highest-scoring candidate
6	P	0.0139	0.0000	0.0000	Covariance Score	Covariance score of highest scoring candidate
7	N	−0.0701	−0.2975	−0.0096	Calibrated Mod. Platt	Calibrated score of highest scoring candidate using Modified Platt scores
8	N	−0.0237	−0.1039	0.0000	Calibrated Covariance	Calibrated score of highest scoring candidate using Covariance scores
9	P	0.0185	0.0137	0.0149	FT Explained Intensity	Sum of normalized peak intensities in the input spectrum which are "explained" by the SIRIUS fragmentation tree
10	N	−0.0753		−0.0900	Log No. Candidates	Logarithm of candidate list size
11	P	0.0000	0.0000	0.0000	FT Score	Score of the SIRIUS fragmentation tree
12	P	0.0000	0.0000	0.0000	Fingerprint Quality	"Quality" of the predicted fingerprint, measured as $\sum_i \max\{1-p_i, p_i\}$ for predicted fingerprint $(p_1,\ldots,p_n)$
13	N	−0.0423		0.0000	Tanimoto Sim. runner-up	Tanimoto Similarity between highest-scoring and runner-up candidate
14	P	0.0012	0.0000	0.0000	Tanimoto Sim. predicted	Tanimoto Similarity between predicted fingerprint and highest-scoring candidate fingerprint
15	P	0.0178	0.0449	0.0234	FP Length Pred.	Cardinality of predicted fingerprint, only properties with posterior probability at least 0.5 are counted
16	P	0.0569	0.1178	0.0314	FP Length Hit	Cardinality of highest ranked candidate's fingerprint
17	P	0.0907	0.0718	0.0480	Rescoring 1	Score of the highest-scoring covariance candidate when scored with the Modified Platt scoring method
18	P	0.0243	0.0000	0.0000	Rescoring 2	Score of the highest-scoring Modified Platt candidate when scored with the covariance scoring method
19	N	0.0000	0.0000	−0.0294	Rescoring Calibrated	Calibrated score of highest scoring Modified Platt scoring candidate, when scored with the covariance scoring
20	P	0.0119	0.0219		Score Diff Bio Pub	Score difference of the top hit in the biomolecular structure database and PubChem, Modified Platt scoring
21	P	0.0133	0.0257		Score Diff Bio Pub	Score difference of the top hit in the biomolecular structure database and PubChem, covariance scoring
22		−0.1119	−0.4195		Log No. Cand. PC	Logarithm of candidate list size in PubChem

Supplementary Table 4: MassIVE accession numbers for the Orbitrap dataset. Corresponding mzML/mzXML files are available from MassIVE (https://massive.ucsd.edu/).

MSV000084873, MSV000084753, MSV000084744, MSV000084741, MSV000084738, MSV000084674, MSV000084630, MSV000084628, MSV000084585, MSV000084576, MSV000084556, MSV000084312, MSV000084289, MSV000084278, MSV000084237, MSV000084143, MSV000084132, MSV000084119, MSV000084118, MSV000084117, MSV000084112, MSV000084107, MSV000084102, MSV000084072, MSV000084062, MSV000084045, MSV000084030, MSV000084020, MSV000084016, MSV000083889, MSV000083888, MSV000083791, MSV000083773, MSV000083749, MSV000083705, MSV000083660, MSV000083651, MSV000083647, MSV000083632, MSV000083631, MSV000083612, MSV000083541, MSV000083523, MSV000083522, MSV000083521, MSV000083483, MSV000083481, MSV000083482, MSV000083475, MSV000083472, MSV000083471, MSV000083470, MSV000083469, MSV000083411, MSV000083396, MSV000083395, MSV000083387, MSV000083383, MSV000083372, MSV000083365, MSV000083306, MSV000083300, MSV000083275, MSV000083274, MSV000083272, MSV000083134, MSV000083110, MSV000083106, MSV000083098, MSV000083094, MSV000083083, MSV000083077, MSV000083073, MSV000082999, MSV000082952, MSV000082869, MSV000082650, MSV000082649, MSV000082647, MSV000082633, MSV000082618, MSV000082616, MSV000082614, MSV000082612, MSV000082608, MSV000082602, MSV000082582, MSV000082480, MSV000082463, MSV000082433, MSV000082402, MSV000082385, MSV000082384, MSV000082383, MSV000082382, MSV000082380, MSV000082379, MSV000082378, MSV000082377, MSV000082331, MSV000082157, MSV000082086, MSV000082085, MSV000082084, MSV000082083, MSV000082082, MSV000082081, MSV000080249, MSV000082048, MSV000081957, MSV000081952, MSV000081949, MSV000081808, MSV000081804, MSV000081492, MSV000081482, MSV000081456, MSV000081097, MSV000080905, MSV000080630, MSV000080628, MSV000079900, MSV000081160

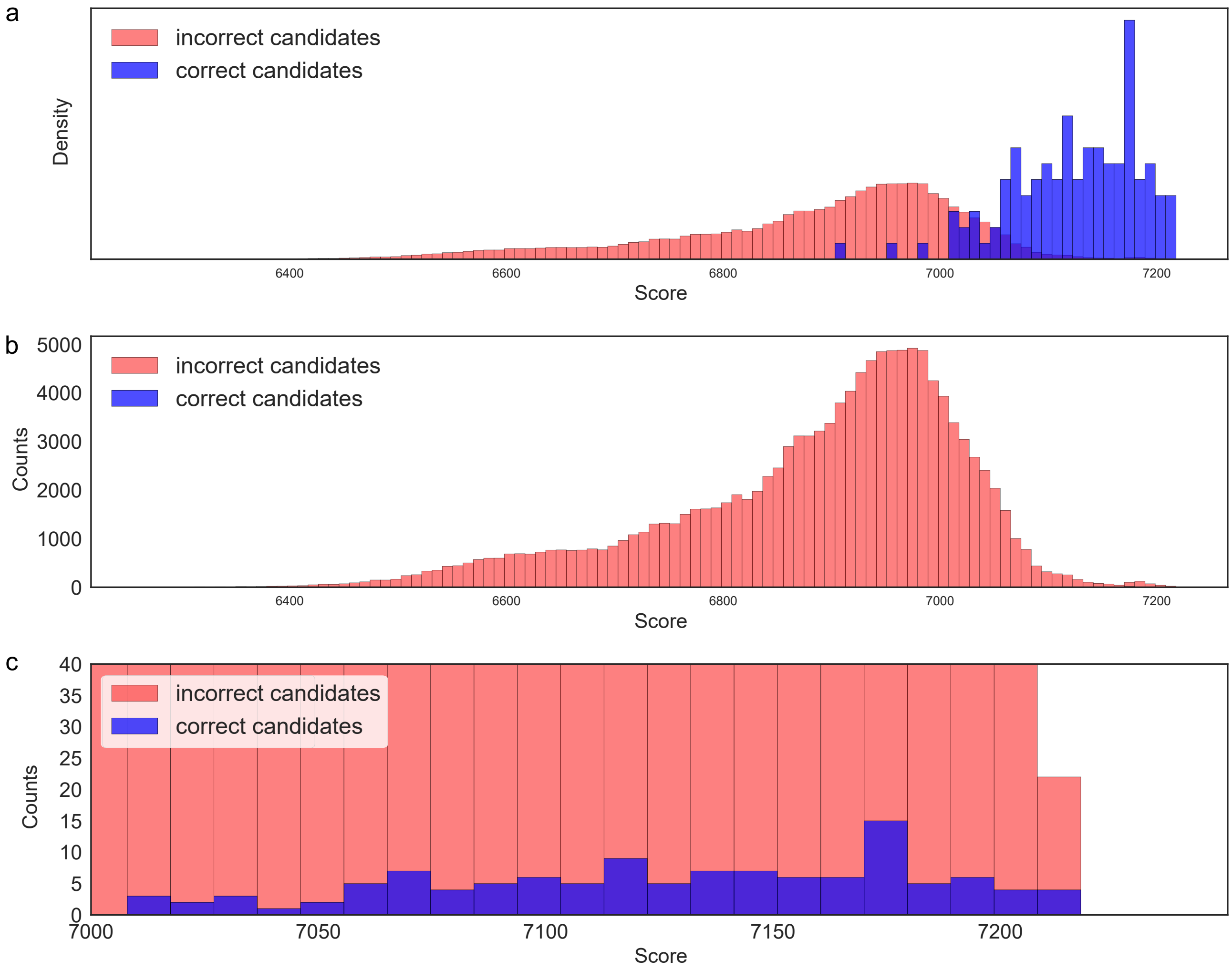

Supplementary Fig. 1: Score distribution of correct and incorrect molecular structure candidates, using CASMI 2016 contest results for CSI:FingerID. Histogram plots displaying all queries and *all candidates* of CASMI 2016 (positive ion mode) simultaneously. There are 120 correct candidates but 123 551 incorrect candidates, so incorrect candidates are three orders of magnitude more common. We plot scores of the original CSI:FingerID submission for CASMI 2016; since CASMI rules required that scores of all candidates are positive, an arbitrary constant value of 10,000 was added to each score. (a) Score distributions when both distributions have been normalized individually. Correct candidates receive a much higher score than a randomly selected incorrect candidate; if this was not the case, then CSI:FingerID would not be able to reach a reasonable annotation rate. (b) Score distributions without normalization; correct candidates are practically invisible in this plot. (c) Zoom-in into (b): We observe numerous incorrect candidates with scores as high as correct candidates.

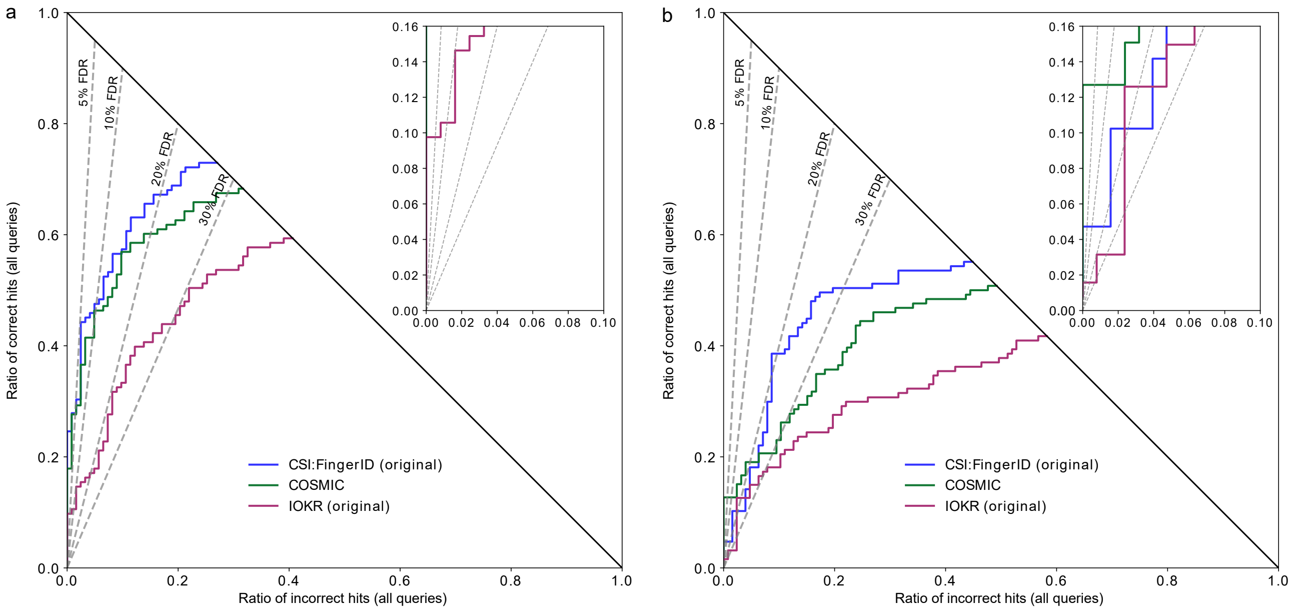

Supplementary Fig. 2: Separation for CSI:FingerID on CASMI 2016 without structure-disjoint evaluation. Hop plots for searching (a) the biomolecule structure database, N = 123 or (b) ChemSpider, N = 127. FDR levels shown as dashed lines. Positive ion mode. "CSI:FingerID (original)" refers to the original CASMI 2016 submission of CSI:FingerID, and "IOKR (original)" is the original submission for its Input Output Kernel Regression (IOKR) variant. In contrast to Fig. 2, these evaluations were not carried out structure-disjoint; CASMI is a blind competition, and the correct answers were unknown to the contestants upon submission. The COSMIC curve is given for comparison; it is copied verbatim from Fig. 2 and *ensures structure-disjoint evaluation*. In agreement with our findings for applying COSMIC without structure-disjoint evaluation (Fig. 5), separation of the original submissions is much better than for CSI:FingerID with structure-disjoint evaluation; but notably, not better than for COSMIC *with* structure-disjoint evaluation. We attribute the increased separation power of the original submissions mostly to the overlap in structures between training and evaluation data.

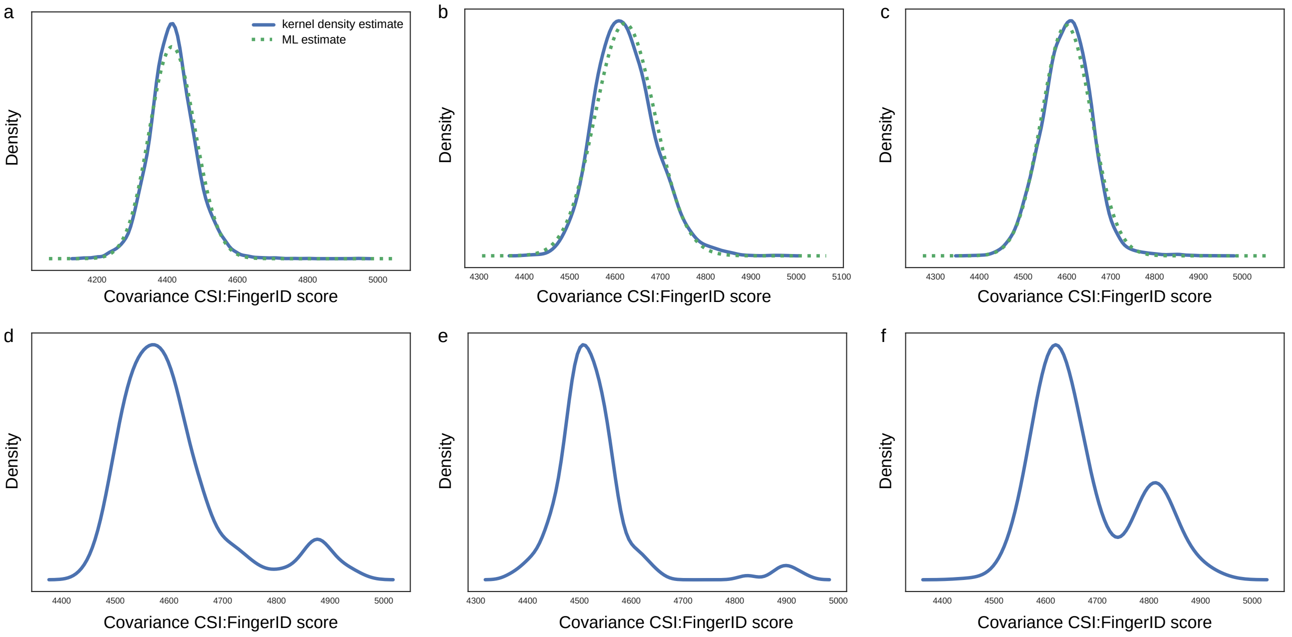

Supplementary Fig. 3: Examples of CSI:FingerID score distributions. Shown are kernel density estimates of candidate scores searching in PubChem. We find that unimodal score distributions (a–c) are often similar to a log-normal distribution ("kernel density estimate"); for comparison, we show the log-normal distribution with parameters fitted by Maximum Likelihood estimation ("ML estimate"). Other score distributions are clearly multimodal (d–f). (a) PyroGlu-Trp, $C_{16}H_{17}N_3O_4$, 4 862 candidates, NIST 1632483. (b) 3-Methyl-L-histidine, $C_7H_{11}N_3O_2$, 3 503 candidates, NIST 1346484. (c) 1,3-Benzodioxole-5-propanamine, $C_{12}H_{17}NO_2$, 15 786 candidates, NIST 1306465. (d) N-(2-Hydroxyethyl)-5(6)-epoxy-8Z,11Z,14Z-eicosatrienamide, $C_{22}H_{37}NO_3$, 471 candidates, NIST 1139175. (e) Methanone, $C_{24}H_{22}FNO_2$, 156 candidates, NIST 1300971. (f) Benzeneethanamine, $C_{18}H_{22}BrNO_3$, 483 candidates, NIST 1380115. Numbers of candidates from PubChem.

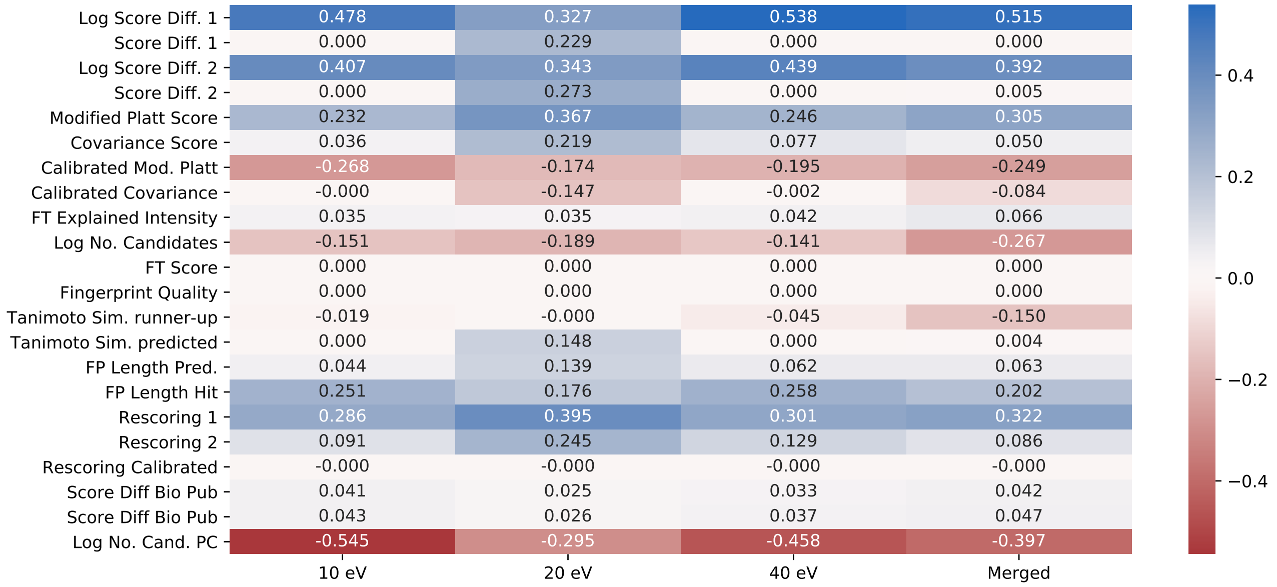

Supplementary Fig. 4: Classifier weights of the COSMIC confidence score. Shown are classifier weights for searching the biomolecular structure database with two or more candidates ("bio_{2+}" in Supplementary Table 1). CSI:FingerID scores are Modified Platt score from Dührkop *et al.* (*Proc Natl Acad Sci USA* 112, 12580–12585, 2015) and Covariance score from Ludwig *et al.* (*Bioinformatics* 34, i333–i340, 2018). Shown are weights for 10 eV, 20 eV, 40 eV and pseudo-ramp spectra ("Merged", all collision energies). Weights for each classifier are normalized to unit norm. We observe that classifiers for 10 eV, 40 eV and merged spectra have similar weights, whereas the classifier for 20 eV distributes weights more uniformly among similar features.

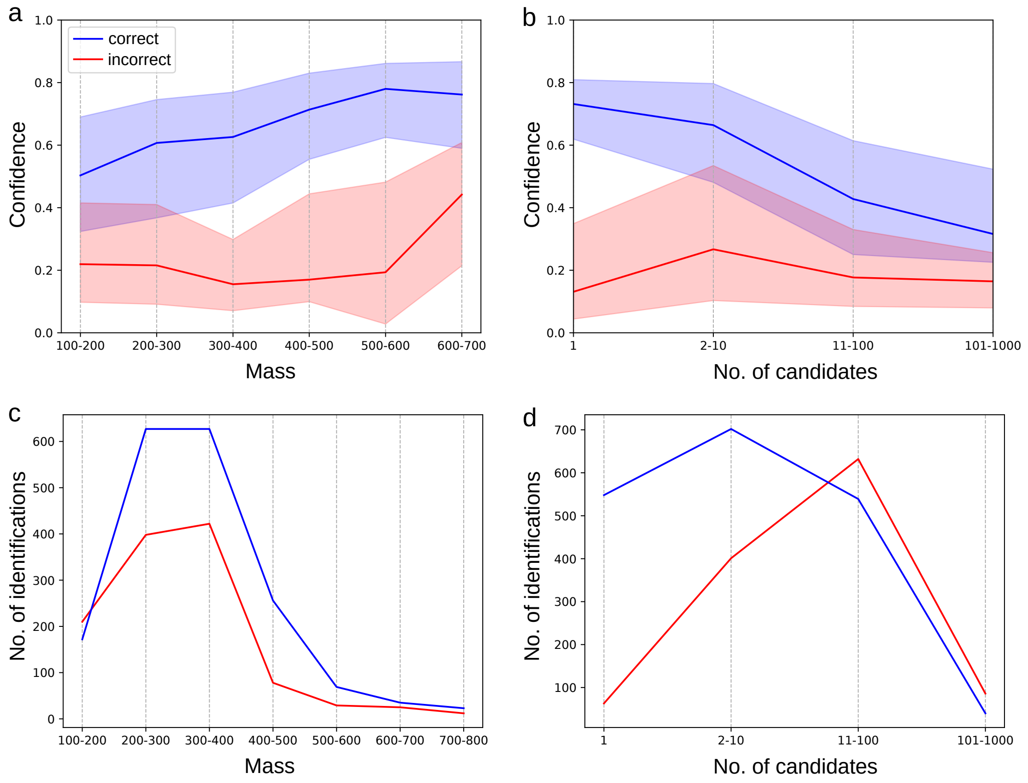

Supplementary Fig. 5: Effect of query compound mass and number of candidates on confidence scores. Independent data, merged spectra ($N = 3\,013$), structure-disjoint evaluation, medium noise, biomolecule structure database. (a,b) Confidence score of correct and incorrect annotations when varying query mass ranges (a) and number of candidates (b). Solid lines show median values, colored areas indicate first (25 %) and third (75 %) quartiles. (c,d) Number of correct and incorrect annotations for varying query mass ranges (c) and number of candidates (d). One compound with mass below 100 Da omitted from (a,c). Only few compounds exist above 500 Da and with more than 100 candidates, so curves (a,b) should be interpreted with care in these regions.

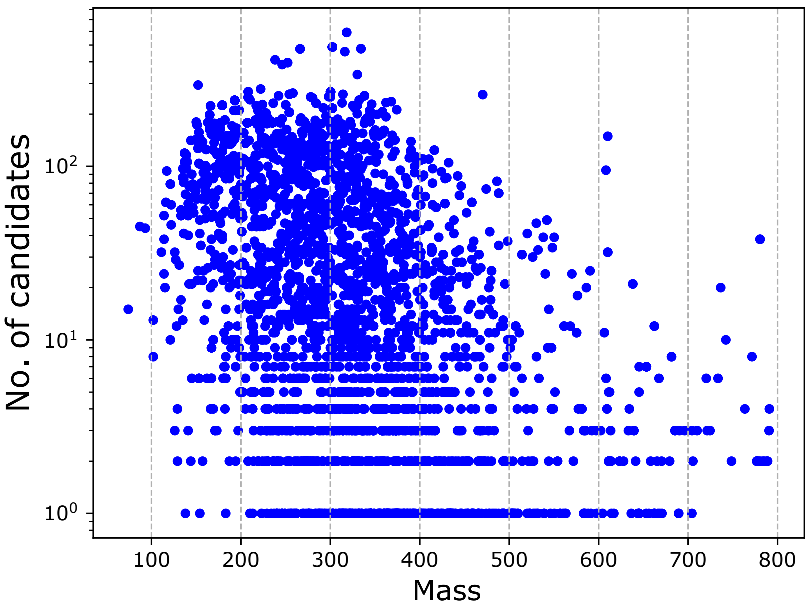

Supplementary Fig. 6: Correlation between the mass of a query compound and the number of candidates retrieved from the database. Note the logarithmic y scale. We display exactly the candidates from Supplementary Fig. 5: Independent data, merged spectra, structure-disjoint evaluation, medium noise, biomolecule structure database.

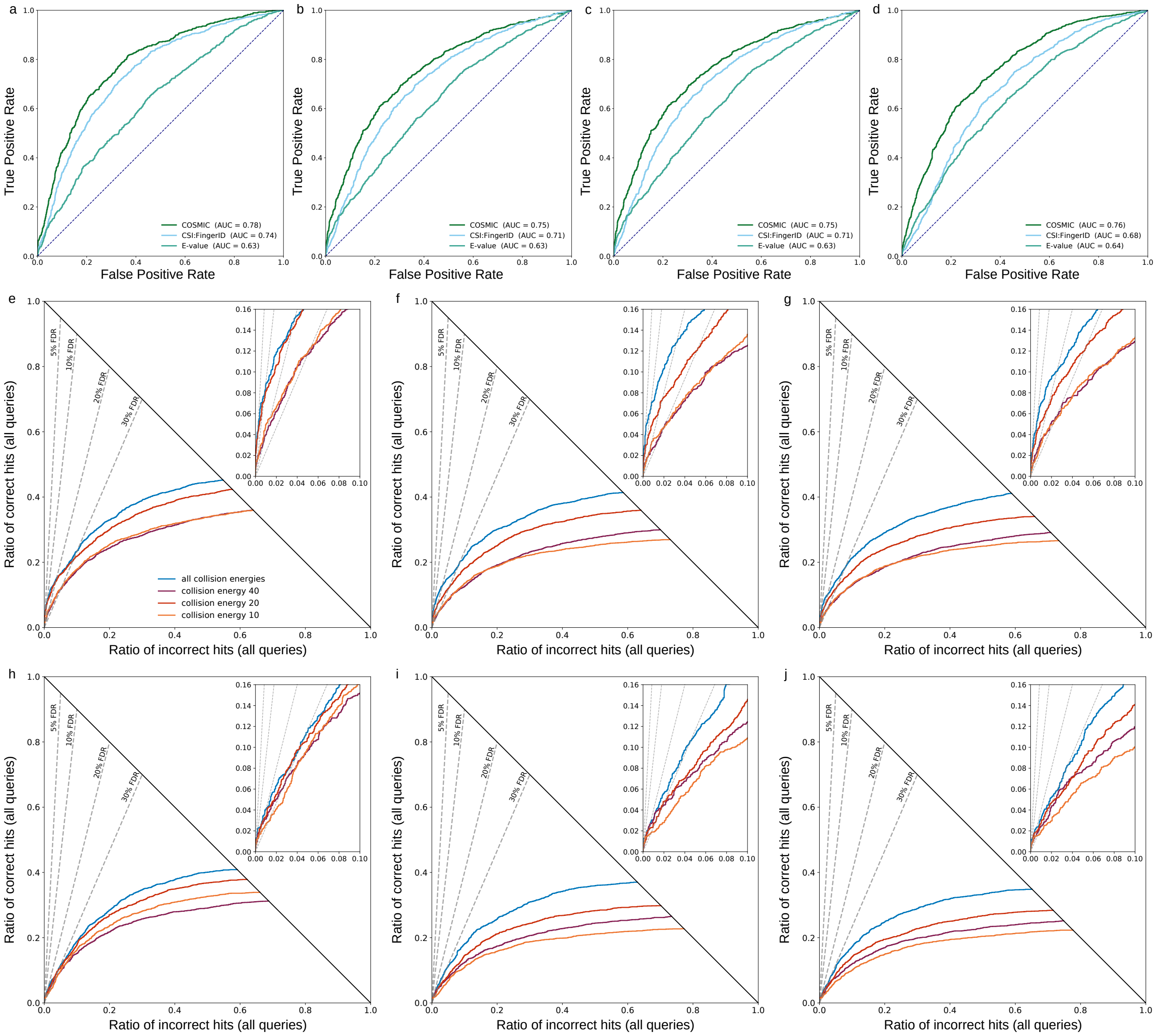

Supplementary Fig. 7: Evaluation of separation searching in PubChem. (a–d) Comparison of CSI:FingerID score, calibrated score and COSMIC confidence score. ROC curves, structure-disjoint evaluation, independent data, medium noise, $N = 3013$. (a) 10 eV, (b) 20 eV, (c) 40 eV, (d) merged spectra ("all collision energies"). Notably, E-values sometimes result in worse separation than the CSI:FingerID score. (e–j) Evaluation of the COSMIC confidence score: Hop plots for different collision energies; notably, these result in substantially different annotation rates. (e–g) Structure-disjoint cross-validation, $N = 3721$. (h–j) Independent data with structure-disjoint evaluation, $N = 3013$. (e,h) No added noise, (f,i) medium noise, (g,j) high noise.

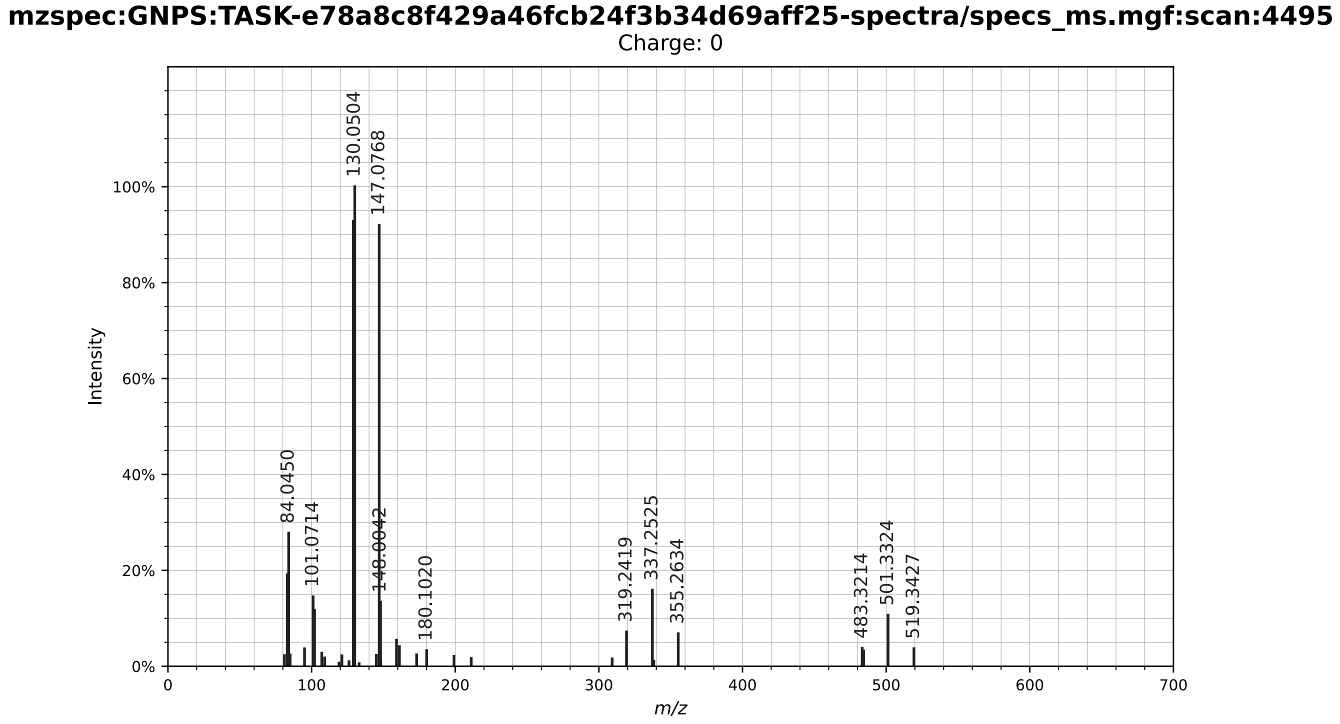

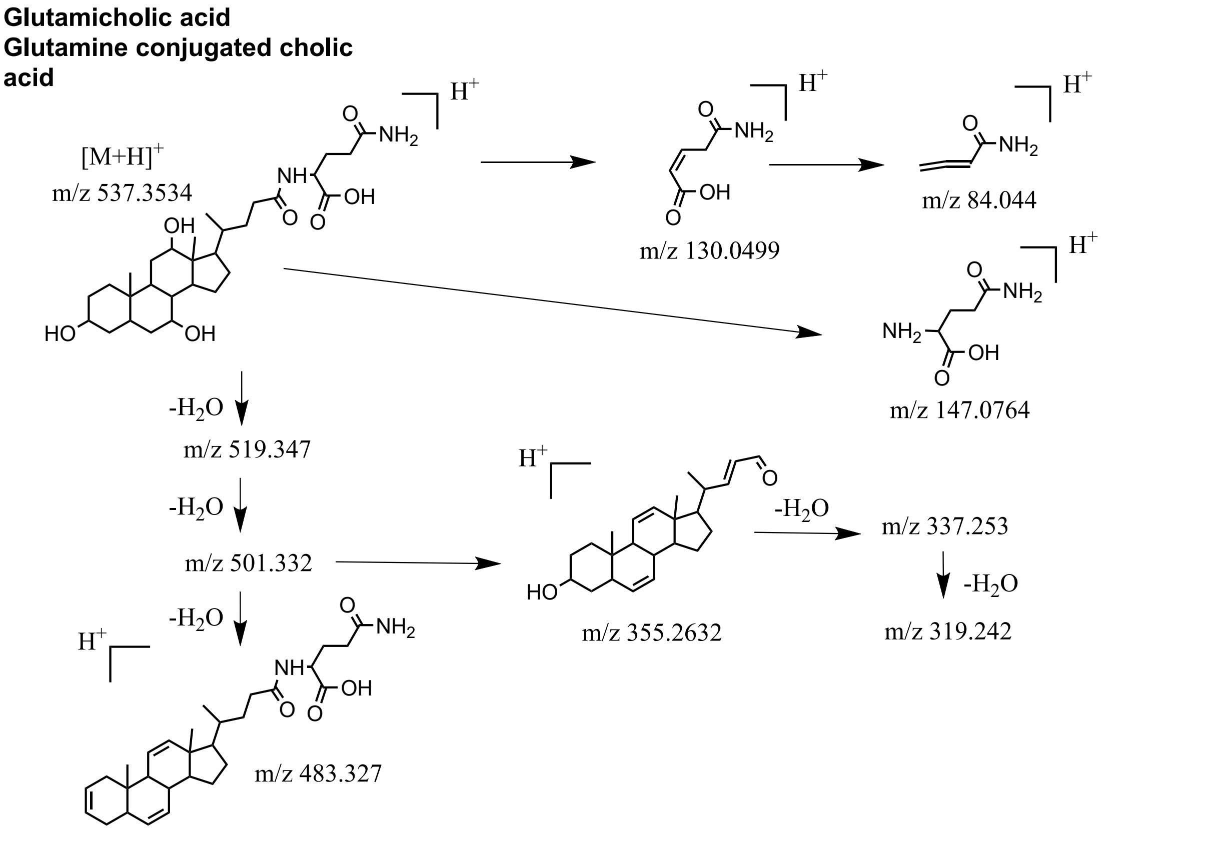

Supplementary Fig. 8: Manual fragmentation analysis of the COSMIC bile acid conjugate 1. Shown is the fragmentation spectrum of m/z 537.354 at 196.2 seconds. Library ID CCMSLIB00005467949, MetabolomicsUSI spectrum link.

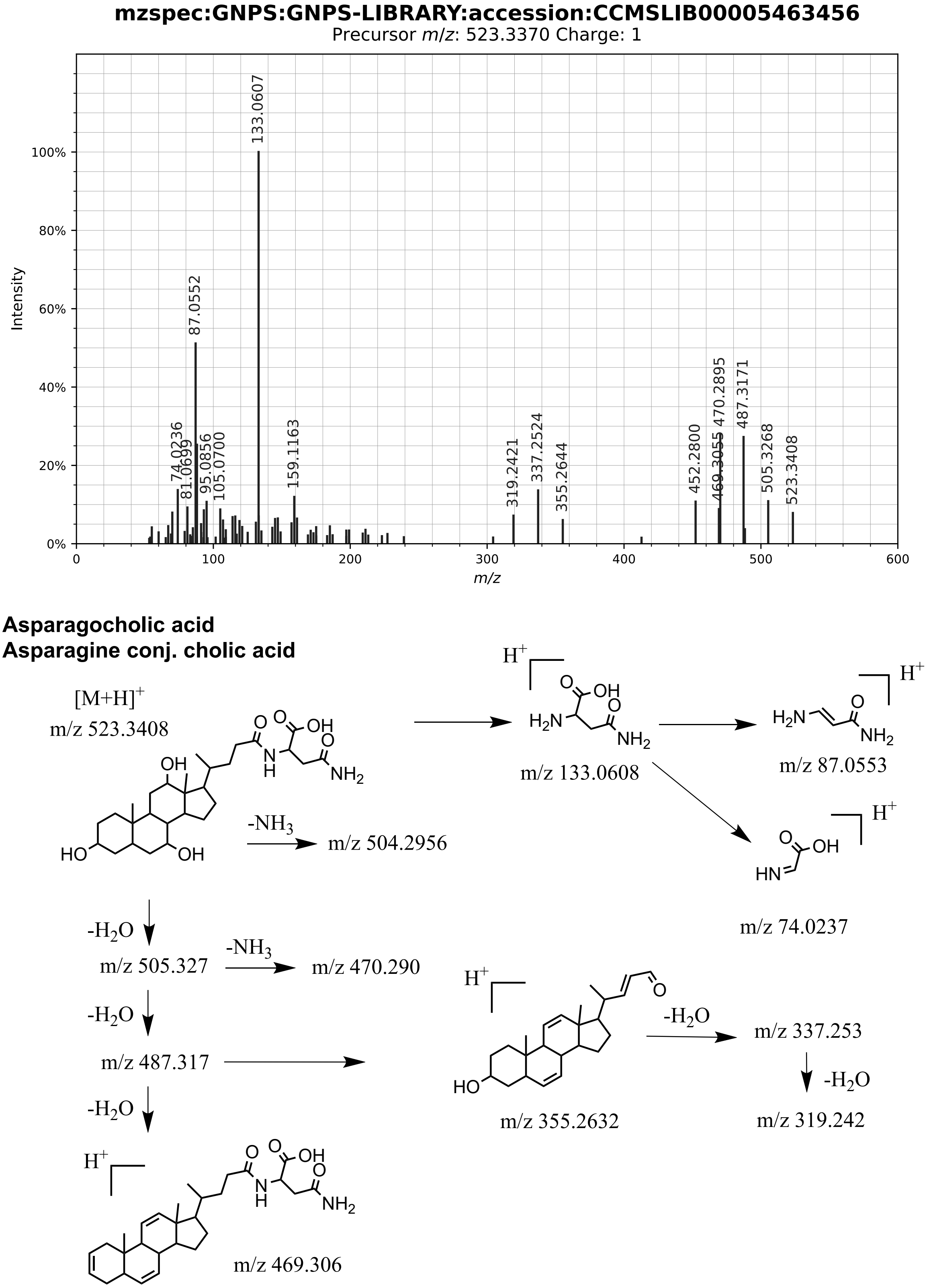

Supplementary Fig. 9: Manual fragmentation analysis of the COSMIC bile acid conjugate 2. Shown is the fragmentation spectrum of m/z 523.3383 at 194.6 seconds. Library ID CCMSLIB00005787996, MetabolomicsUSI spectrum link.

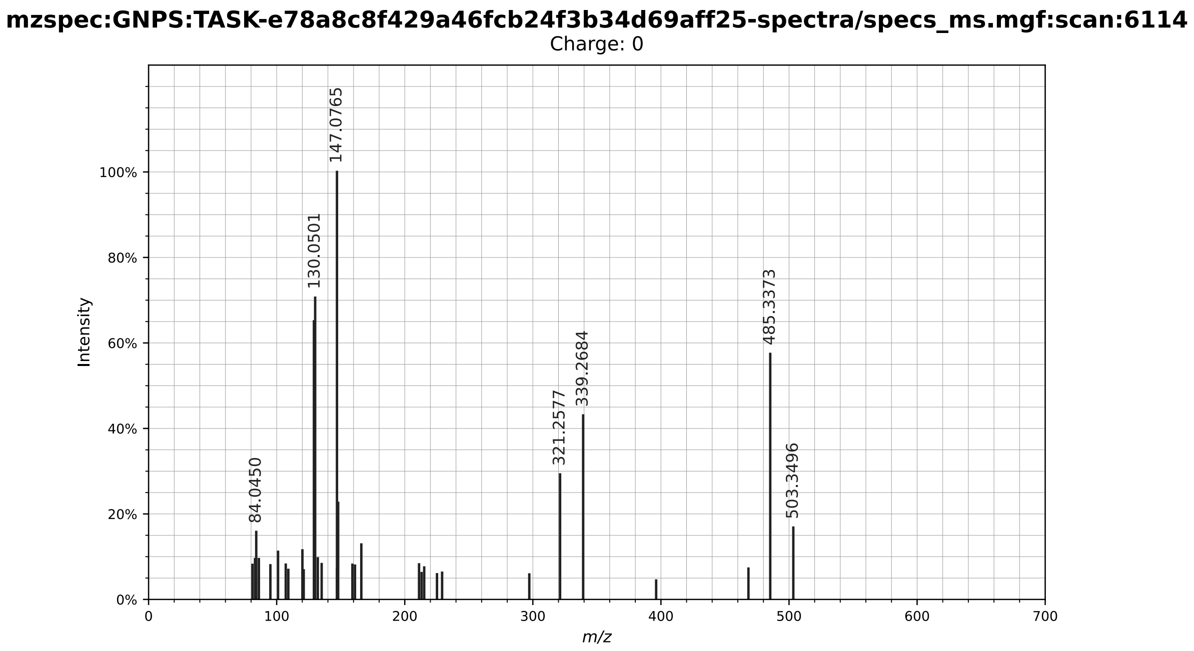

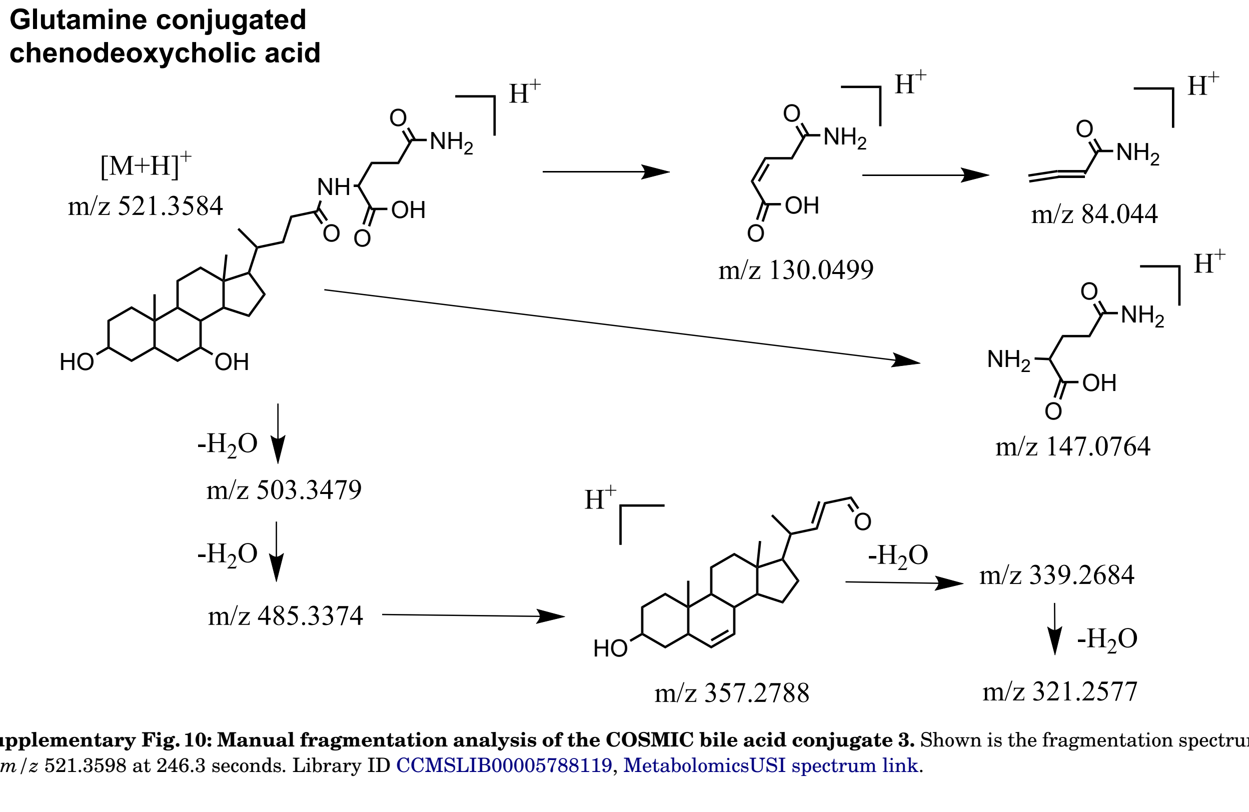

Supplementary Fig. 10: Manual fragmentation analysis of the COSMIC bile acid conjugate 3. Shown is the fragmentation spectrum of m/z 521.3598 at 246.3 seconds. Library ID CCMSLIB00005788119, MetabolomicsUSI spectrum link.

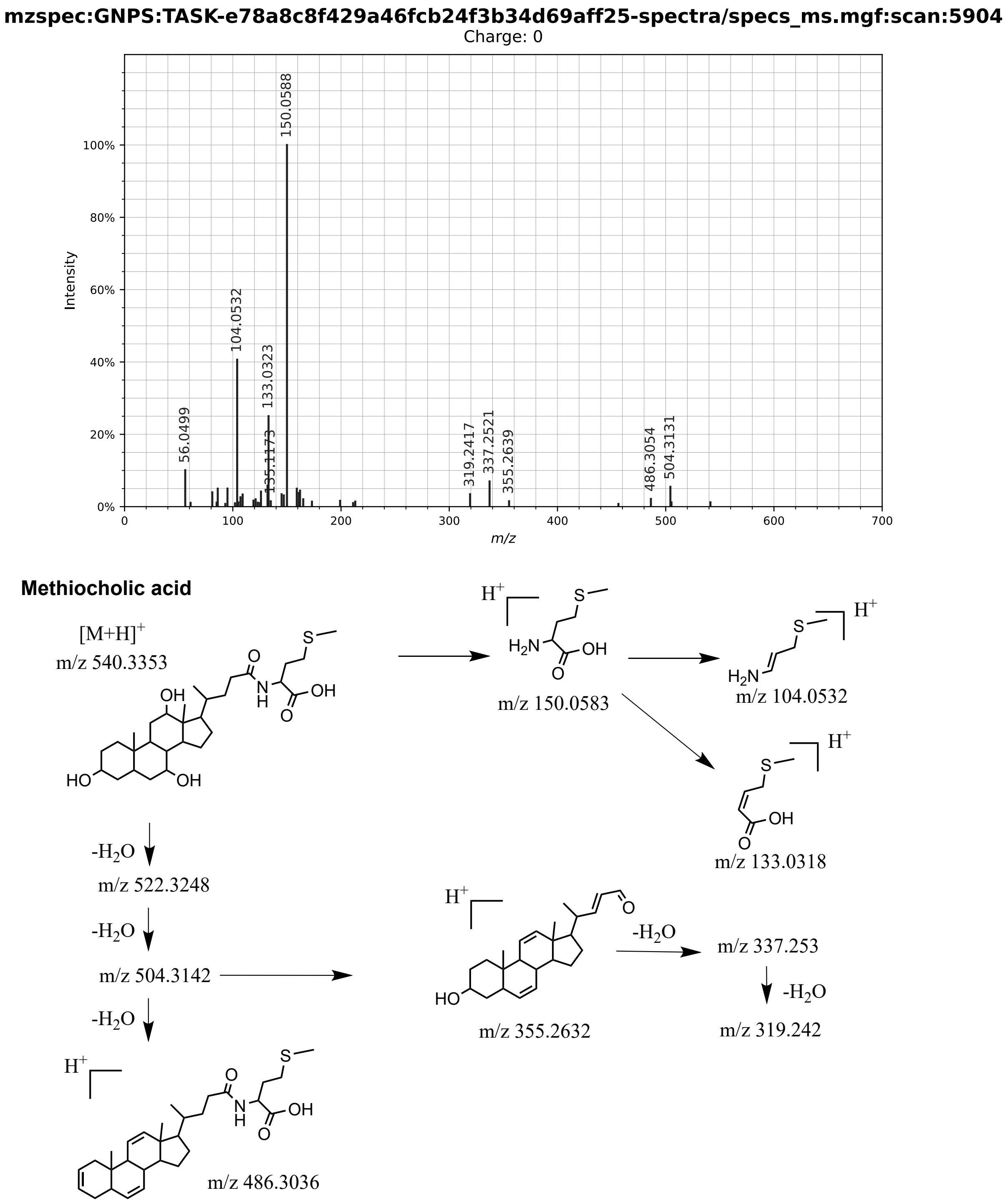

Supplementary Fig. 11: Manual fragmentation analysis of the COSMIC bile acid conjugate 4. Shown is the fragmentation spectrum of m/z 540.3384 at 238.71 seconds. Library ID CCMSLIB00005716809, MetabolomicsUSI spectrum link.

COSMIC annotation 5 - MS/MS m/z 464.3265 at 276 sec.

COSMIC putative candidate
Serinol conj cholic acid [M-H2O+H]+

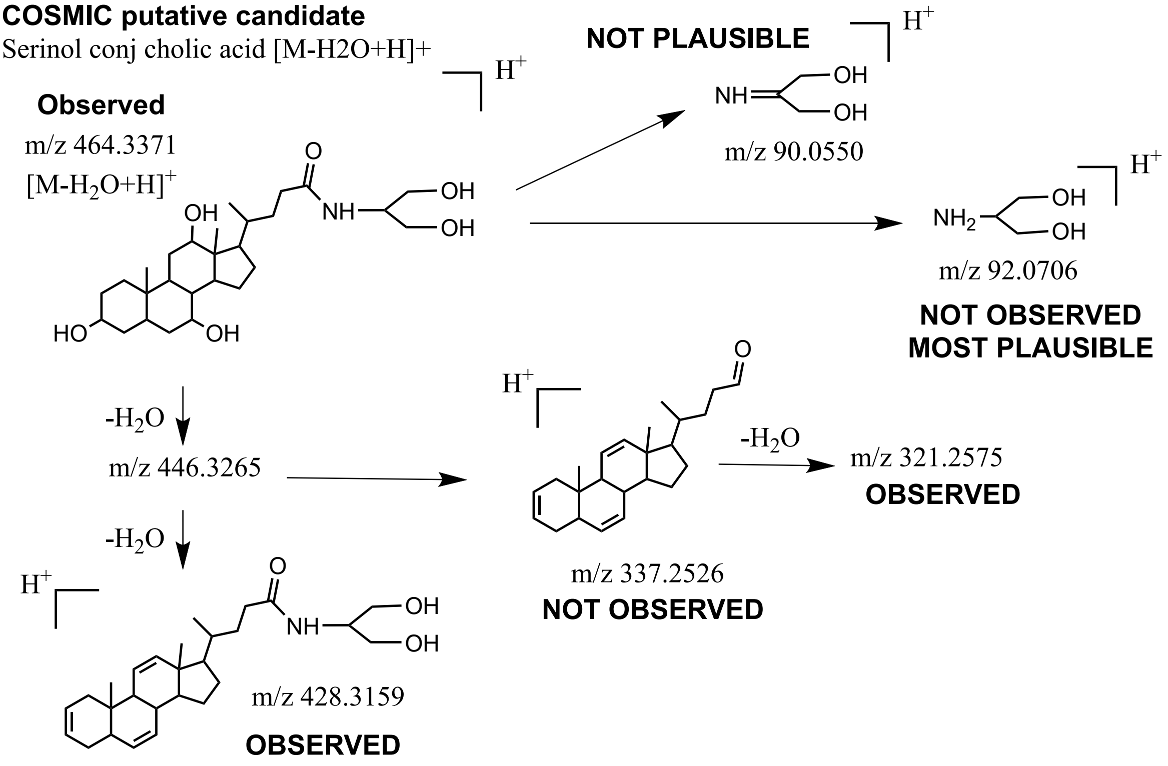

Manual interpretation
Alanine conj chenodeoxycholic acid $[M+H]^+$

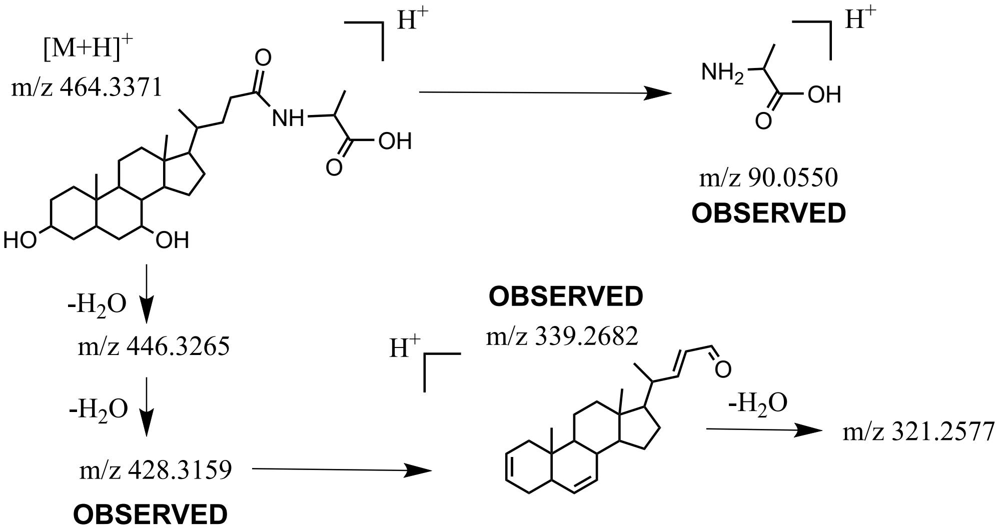

Supplementary Fig. 12: Manual fragmentation analysis of the COSMIC bile acid conjugate 5. Shown is a comparative fragmentation analysis for the fragmentation spectrum of *m/z* 464.3387 at 276.59 seconds. Fragmentation analysis for the COSMIC annotation (Serinol conjugated acid) is shown on top, interpretation of more likely structure (Alanine conjugated chenodeoxycholic acid)on bottom. Library ID CCMSLIB00005788120, MetabolomicsUSI spectrum link.

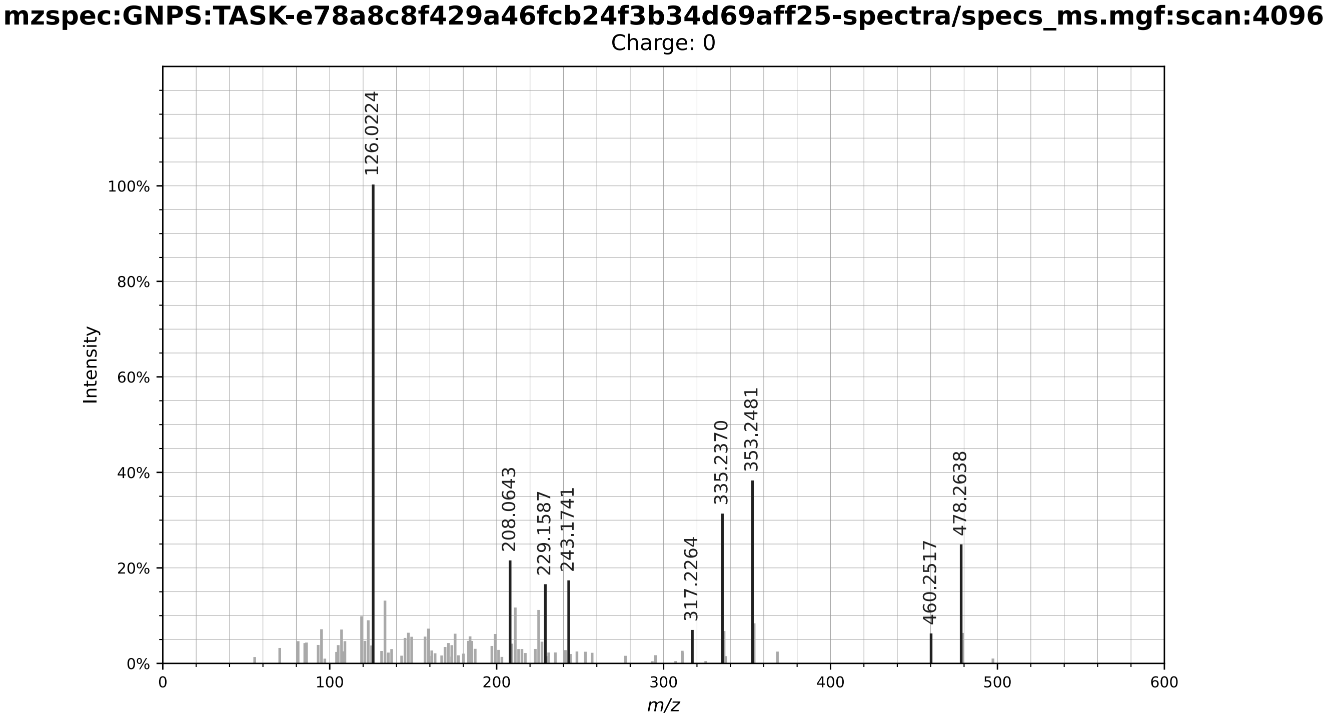

Oxo-taurocholenic acid

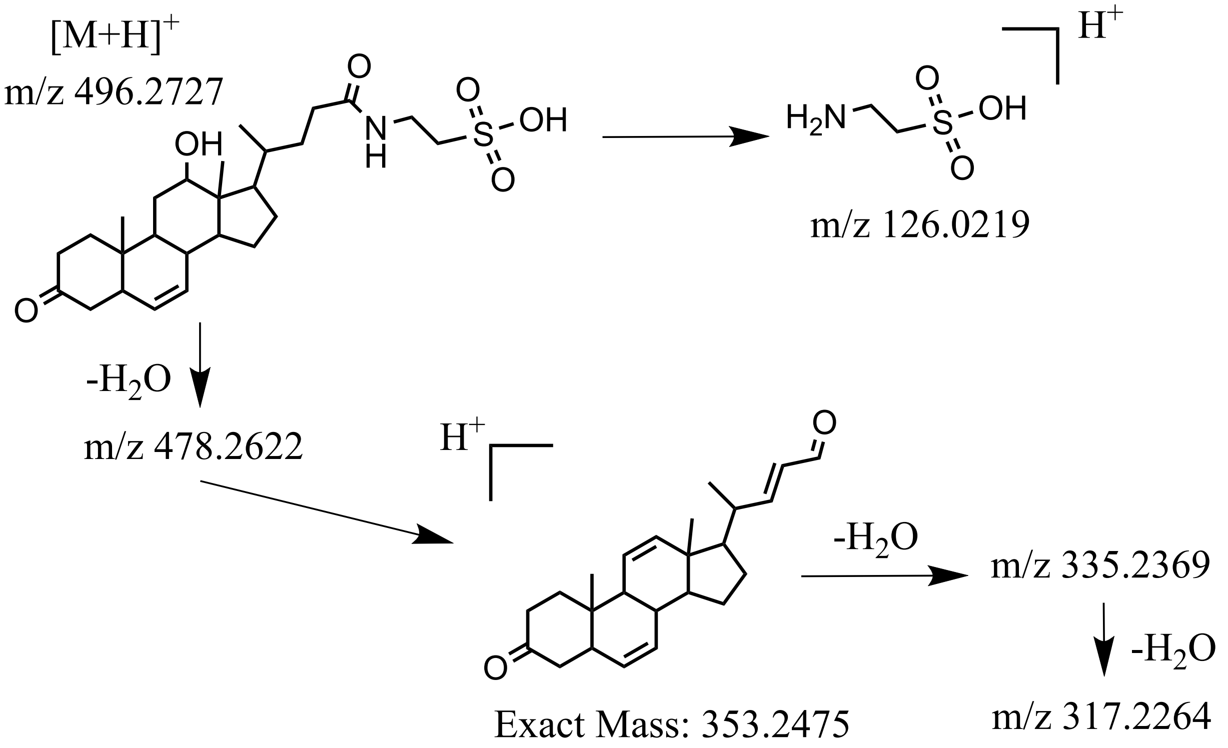

Supplementary Fig. 13: Manual fragmentation analysis of the COSMIC bile acid conjugate 6. Shown is the fragmentation spectrum of m/z 496.2717 at 183.2 seconds. Library ID CCMSLIB00005788121, MetabolomicsUSI spectrum link.

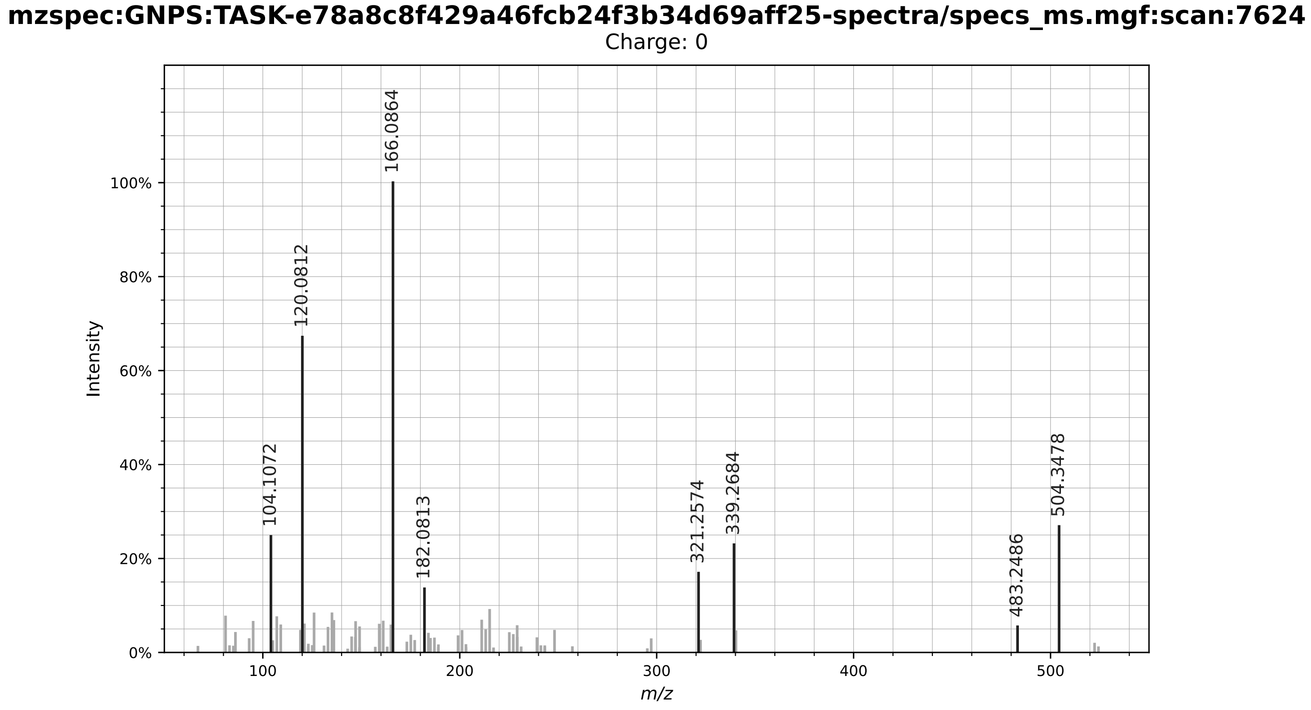

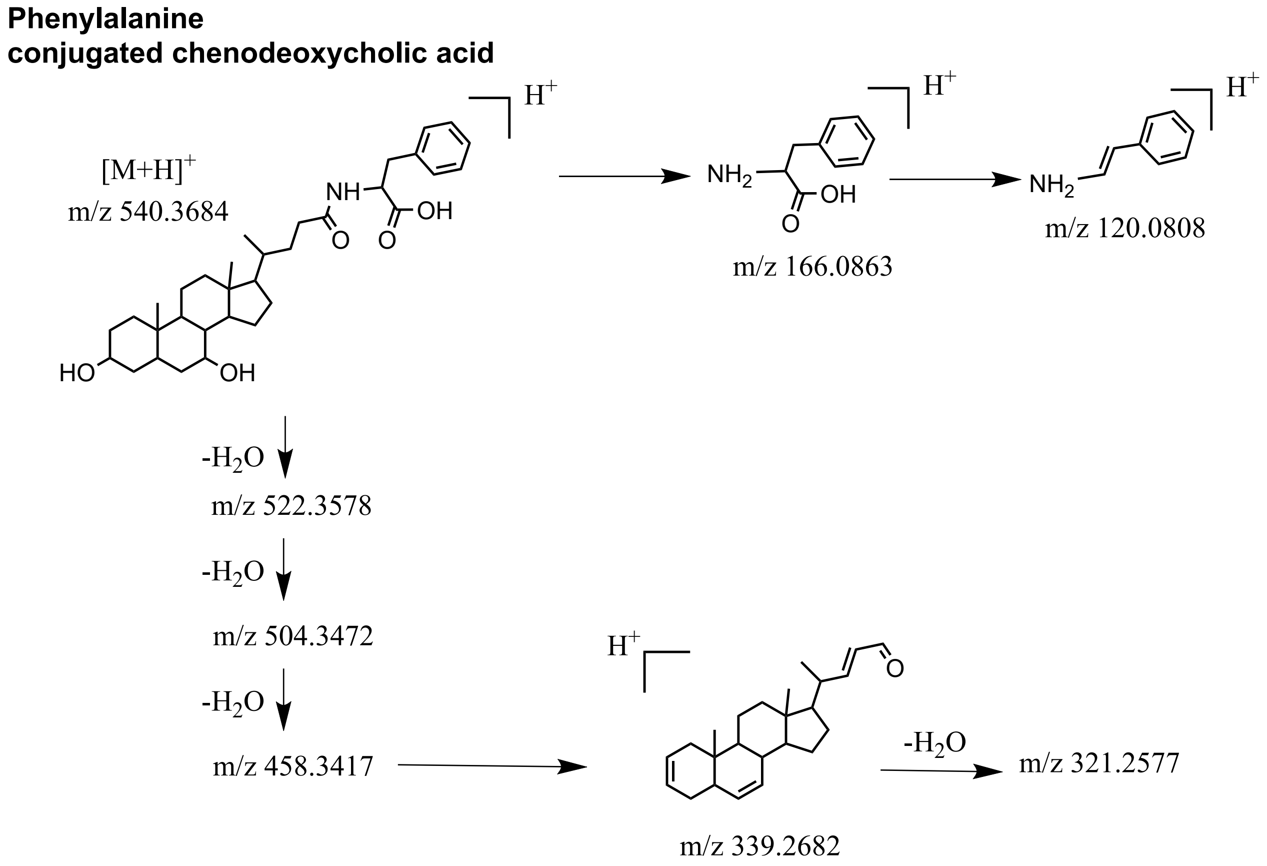

Supplementary Fig. 14: Manual fragmentation analysis of the COSMIC bile acid conjugate 7. Shown is the fragmentation spectrum of m/z 540.3689 at 303.0 seconds. Library ID CCMSLIB00005467952, MetabolomicsUSI spectrum link.

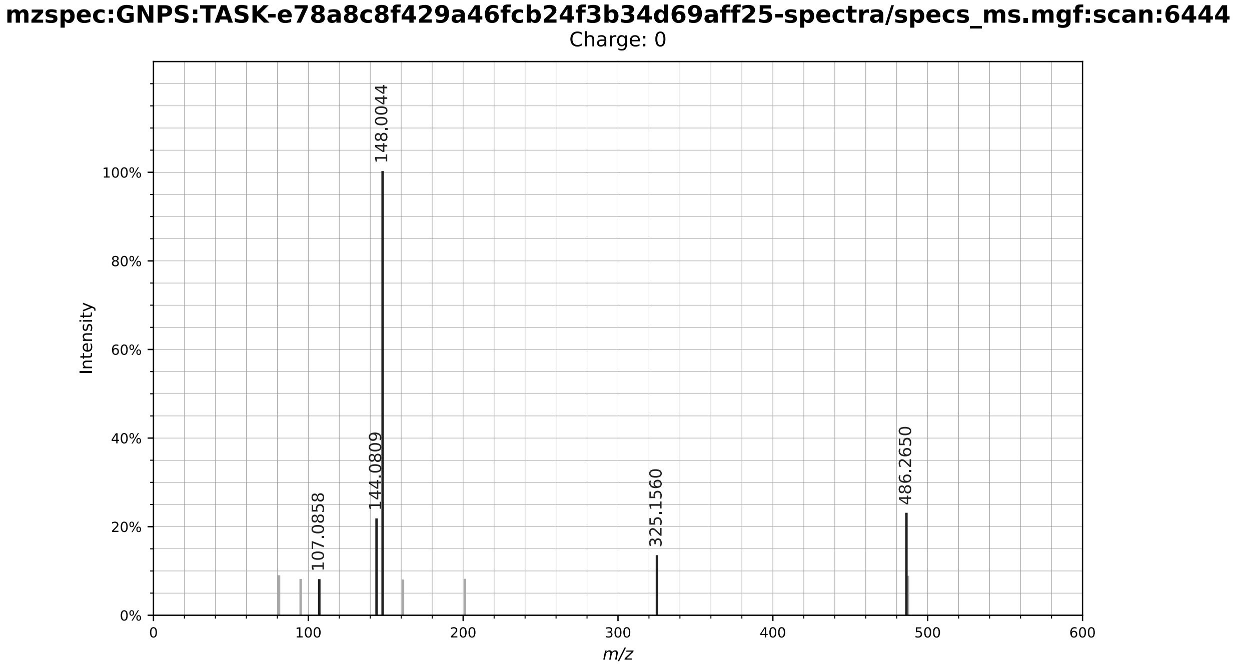

Deoxycholenic acid

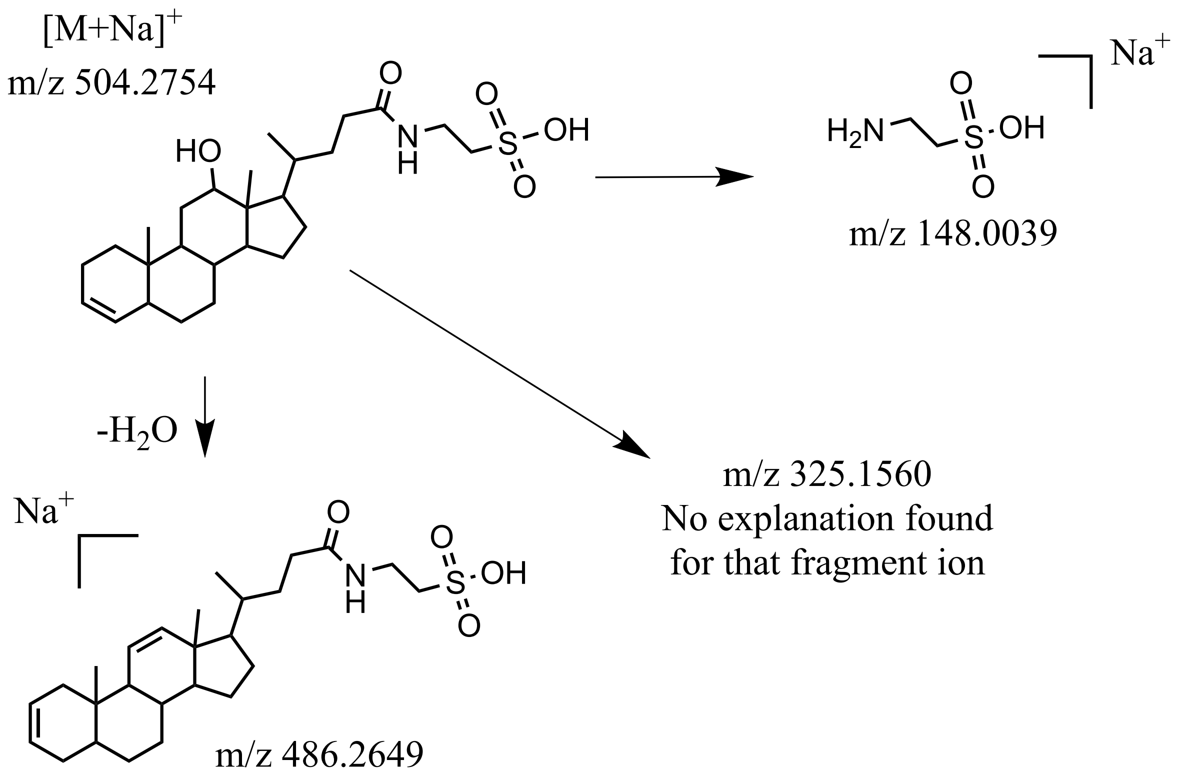

Supplementary Fig. 15: Manual fragmentation analysis of the COSMIC bile acid conjugate 8. Shown is the fragmentation spectrum of m/z 504.2742 at 260.1 seconds. Library ID CCMSLIB00005788122, MetabolomicsUSI spectrum link.

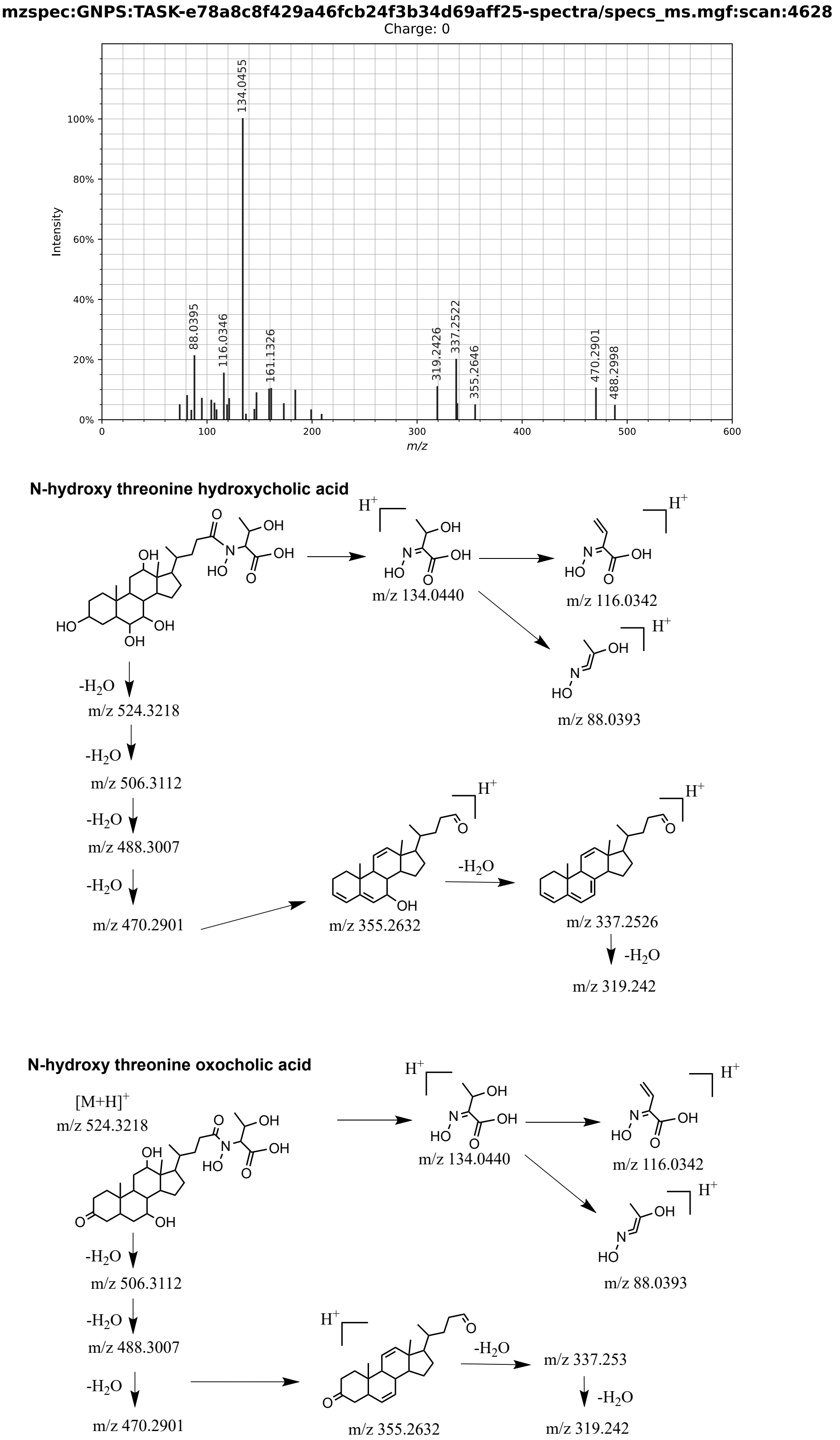

Supplementary Fig. 16: Manual fragmentation analysis of the COSMIC bile acid conjugate 9. Shown is the fragmentation spectrum of m/z 524.3222 at 199.57 seconds. Library ID CCMSLIB00005788123, MetabolomicsUSI spectrum link.

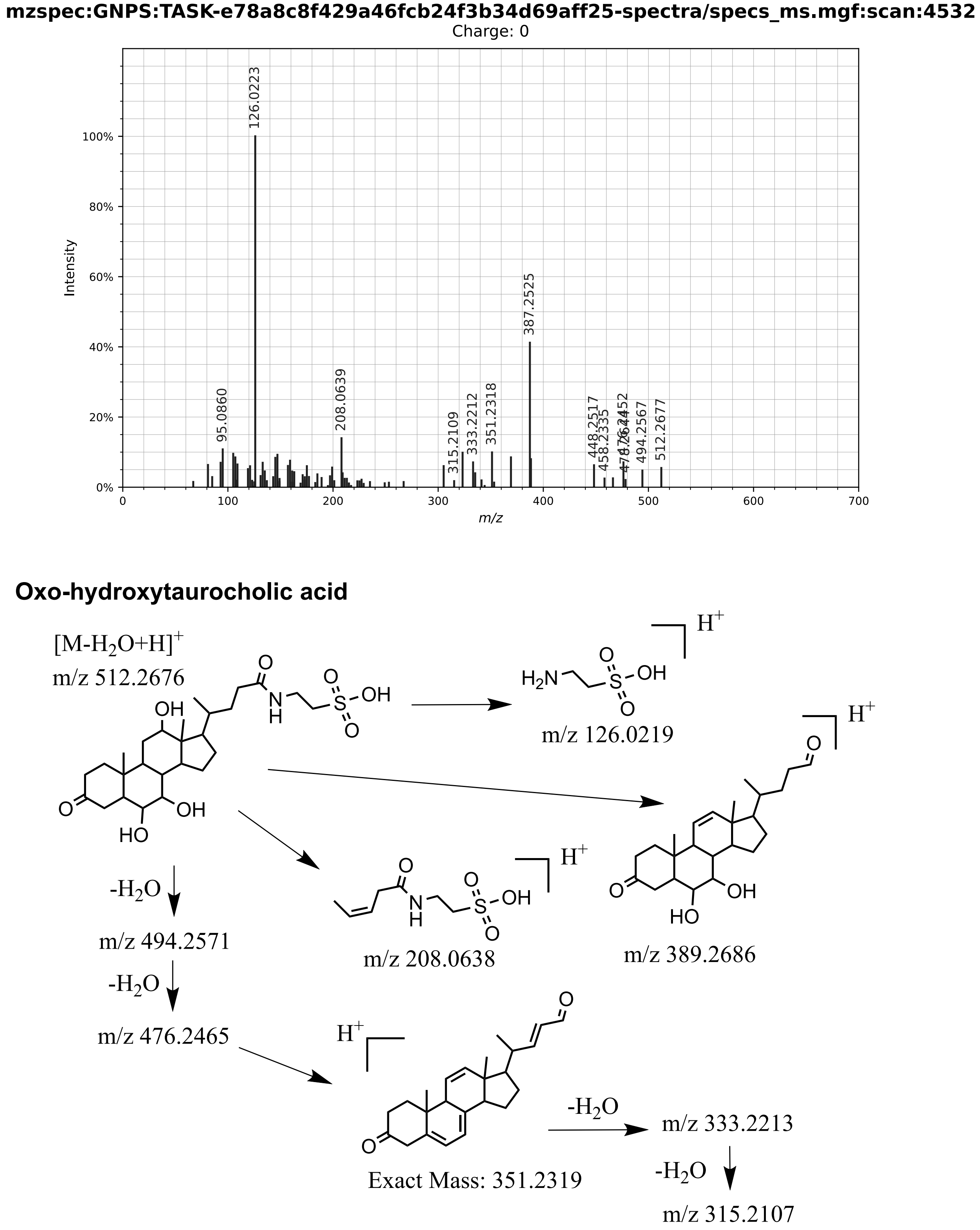

Supplementary Fig. 17: Manual fragmentation analysis of the COSMIC bile acid conjugate 10. Shown is the fragmentation spectrum of m/z 512.2691 at 197.38 seconds. Library ID CCMSLIB00005788124, MetabolomicsUSI spectrum link.

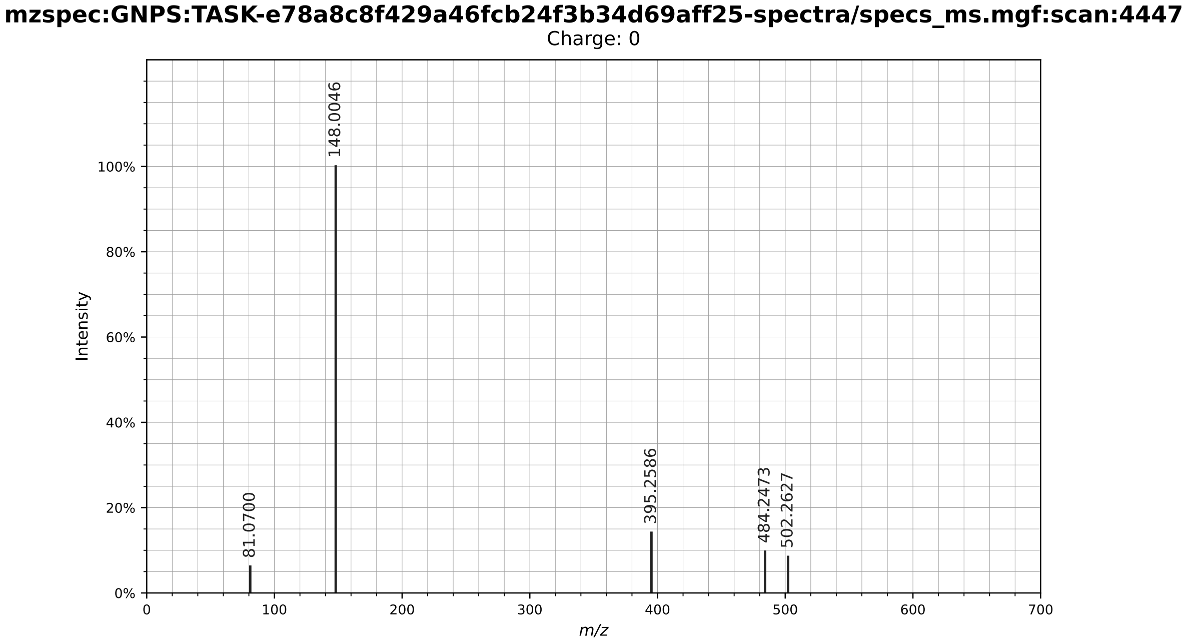

Taurocholenic acid

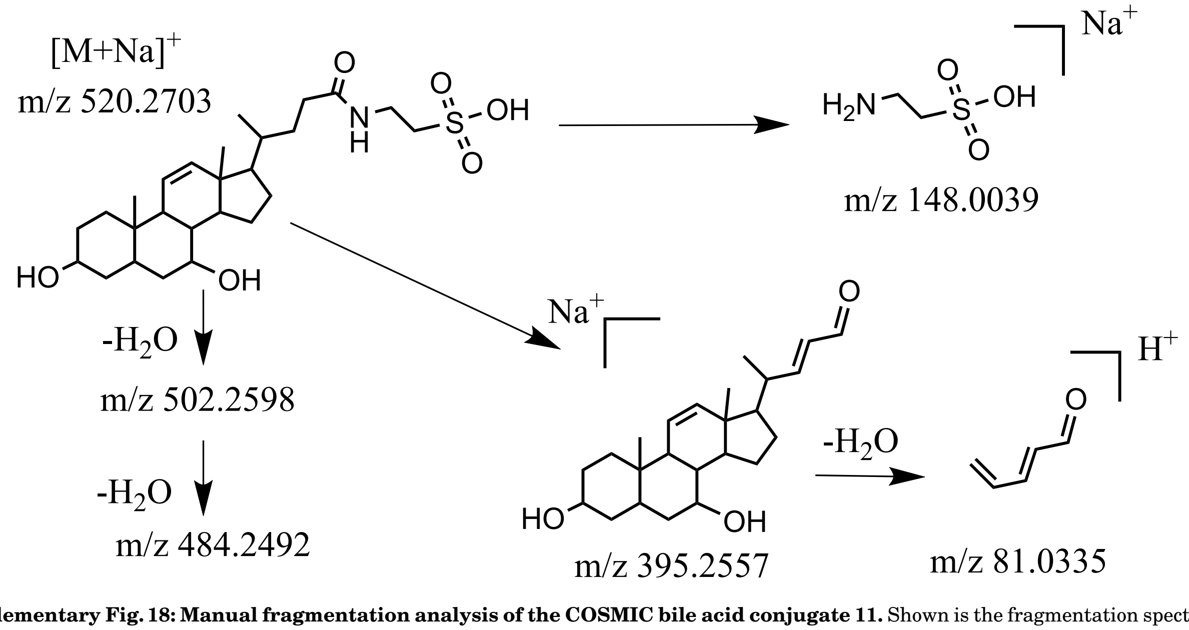

Supplementary Fig. 18: Manual fragmentation analysis of the COSMIC bile acid conjugate 11. Shown is the fragmentation spectrum of m/z 520.2728 at 195.1 seconds. Library ID CCMSLIB00005788125, MetabolomicsUSI spectrum link.

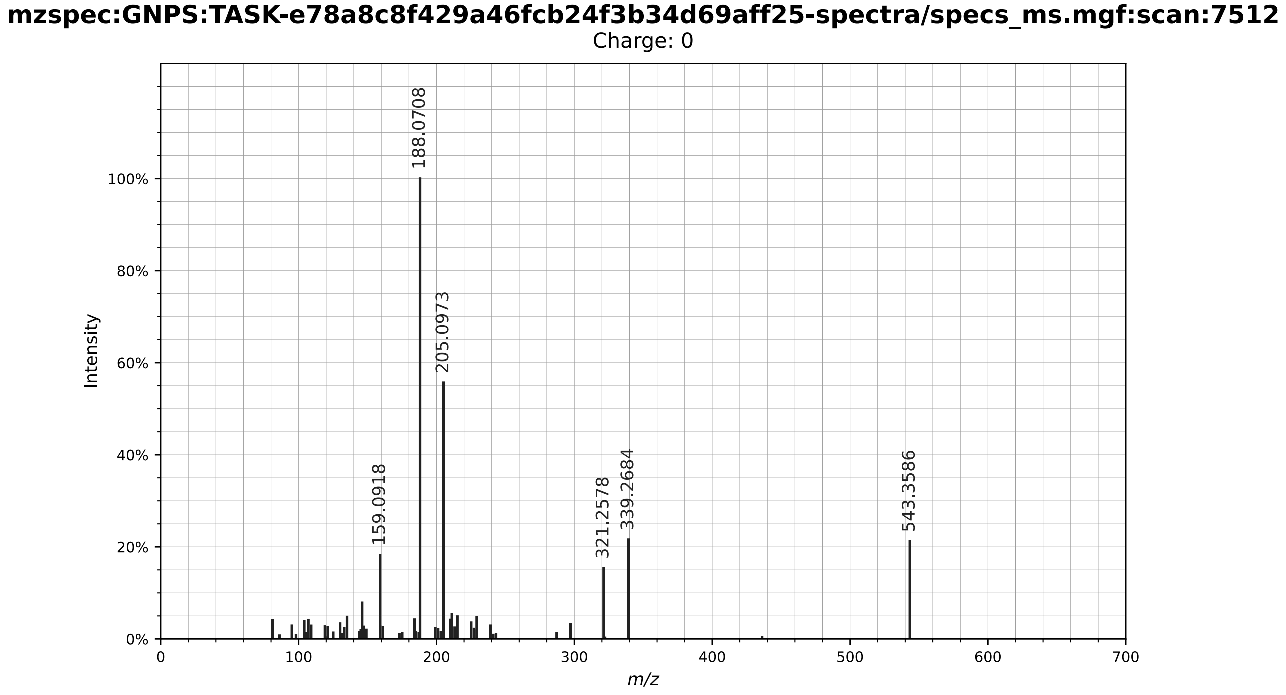

Tryptophan conjugated chenodeoxycholic acid

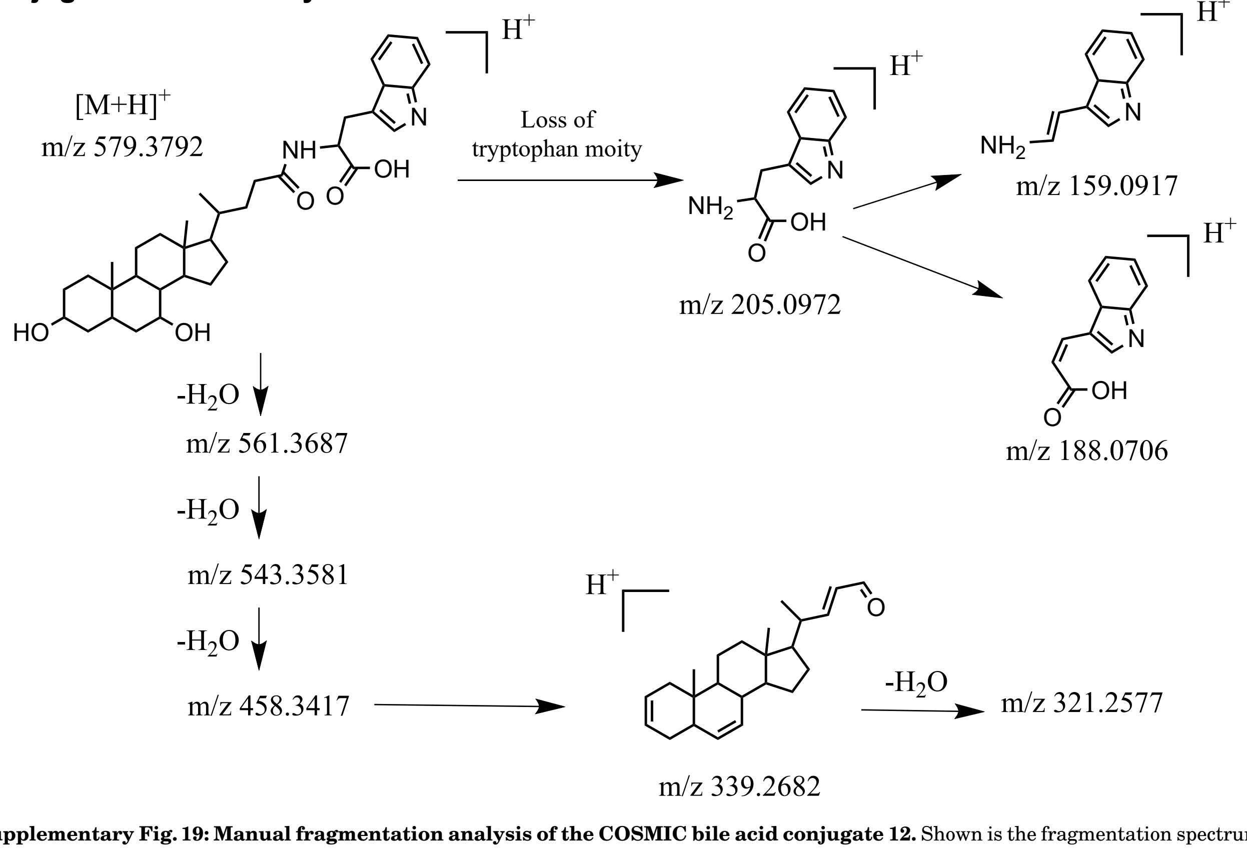

Supplementary Fig. 19: Manual fragmentation analysis of the COSMIC bile acid conjugate 12. Shown is the fragmentation spectrum of m/z 579.3788 at 299.1 seconds. Library ID CCMSLIB00005436493, MetabolomicsUSI spectrum link.

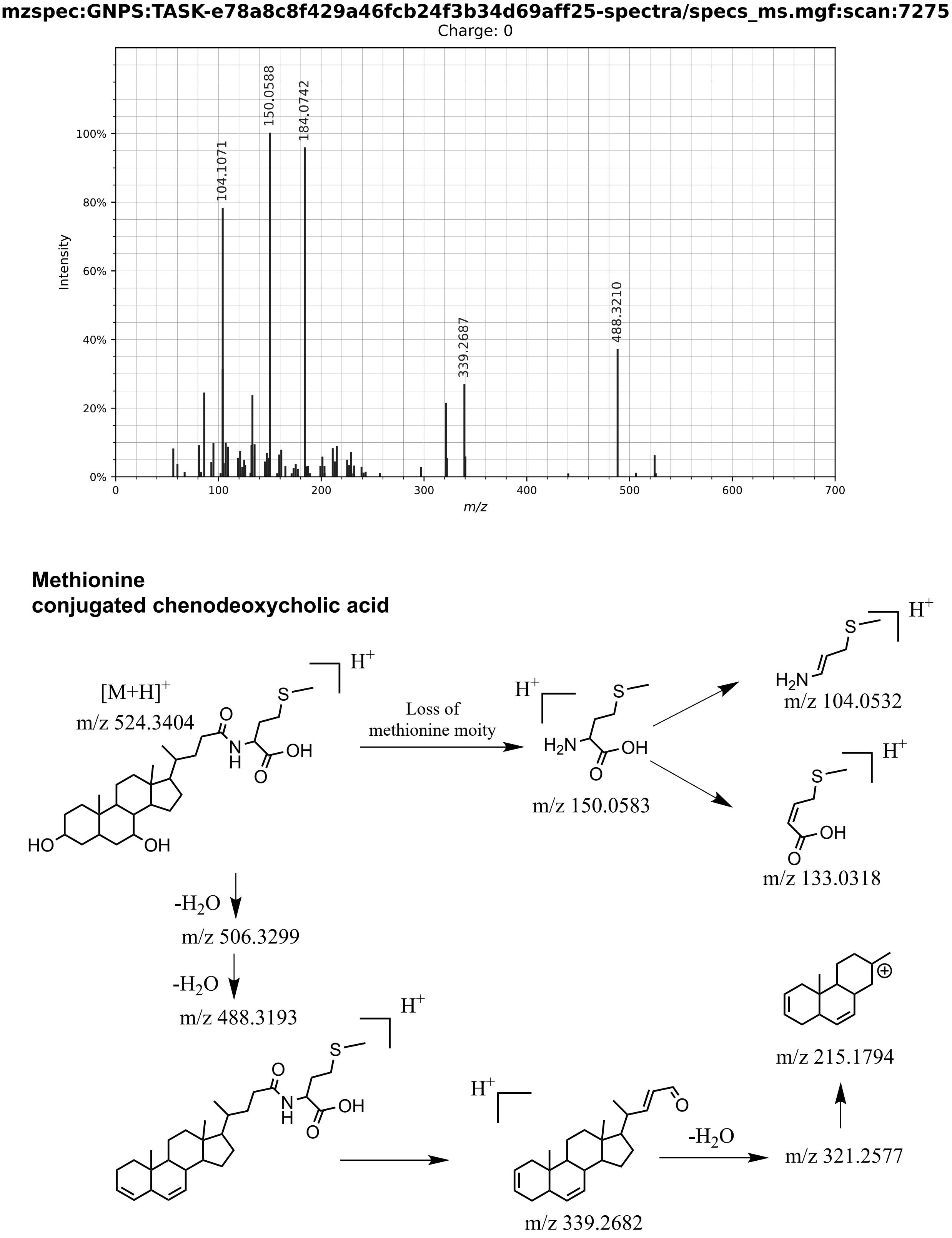

Supplementary Fig. 20: Manual fragmentation analysis of methionine-conjugated CDCA. Shown is the fragmentation spectrum of m/z 524.3392 at 291.99 seconds. Library ID CCMSLIB00005725539, MetabolomicsUSI spectrum link.

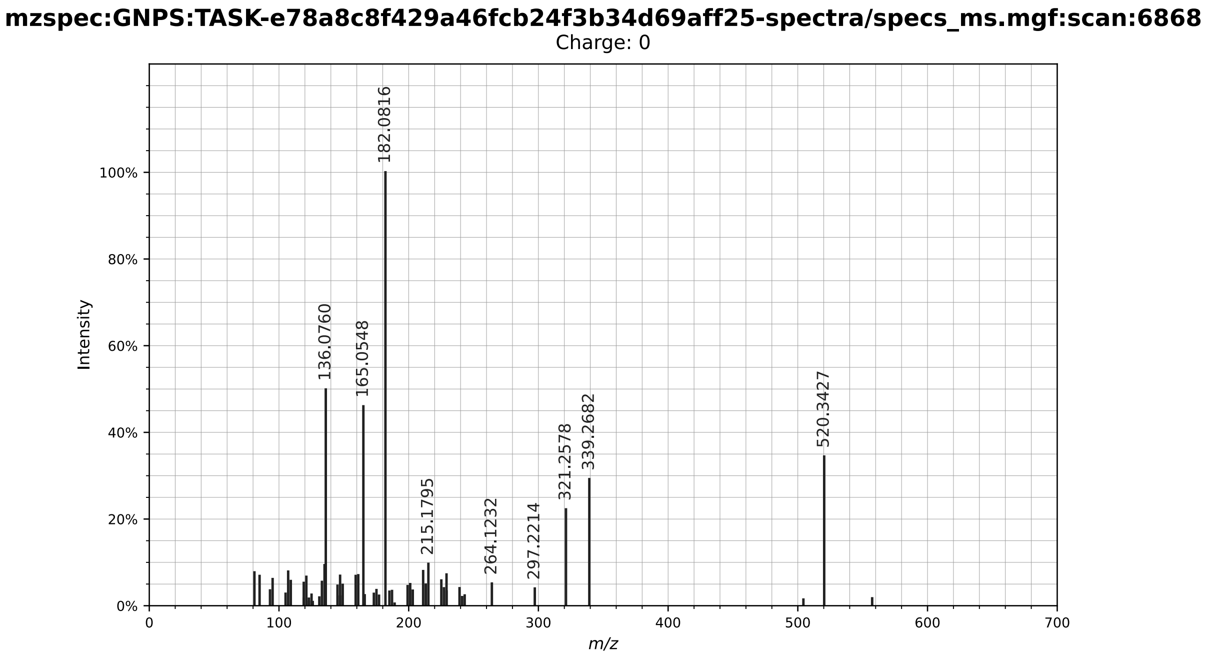

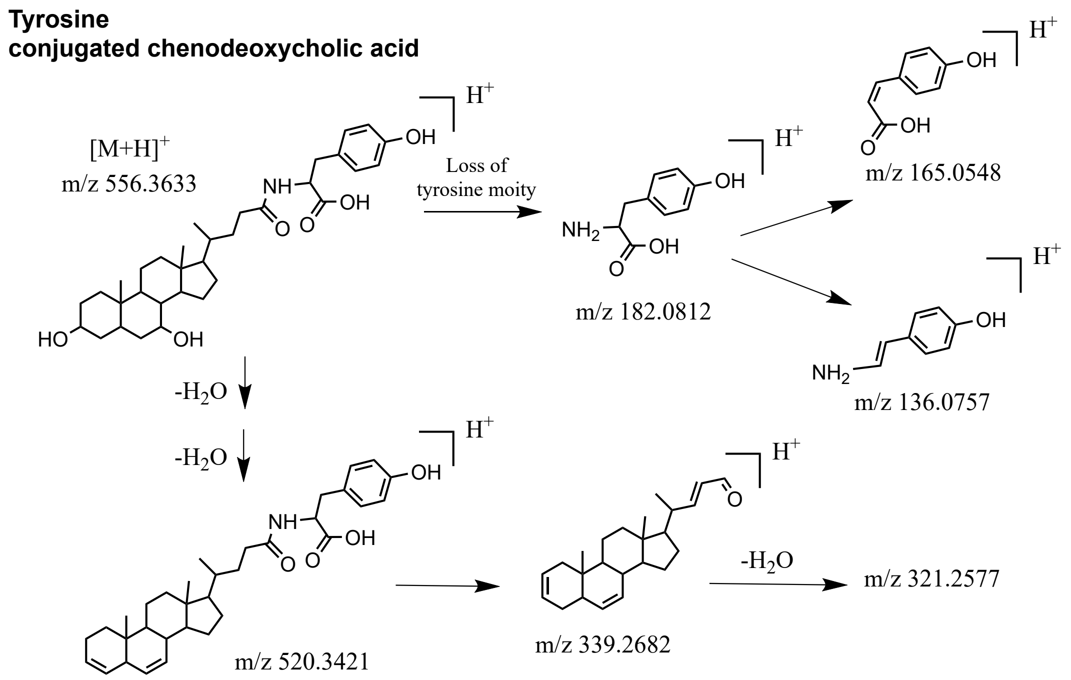

Supplementary Fig. 21: Manual fragmentation analysis of tyrosine-conjugated CDCA. Shown is the fragmentation spectrum of m/z 556.3633 at 278.33 seconds. Library ID CCMSLIB00005467948, MetabolomicsUSI spectrum link.

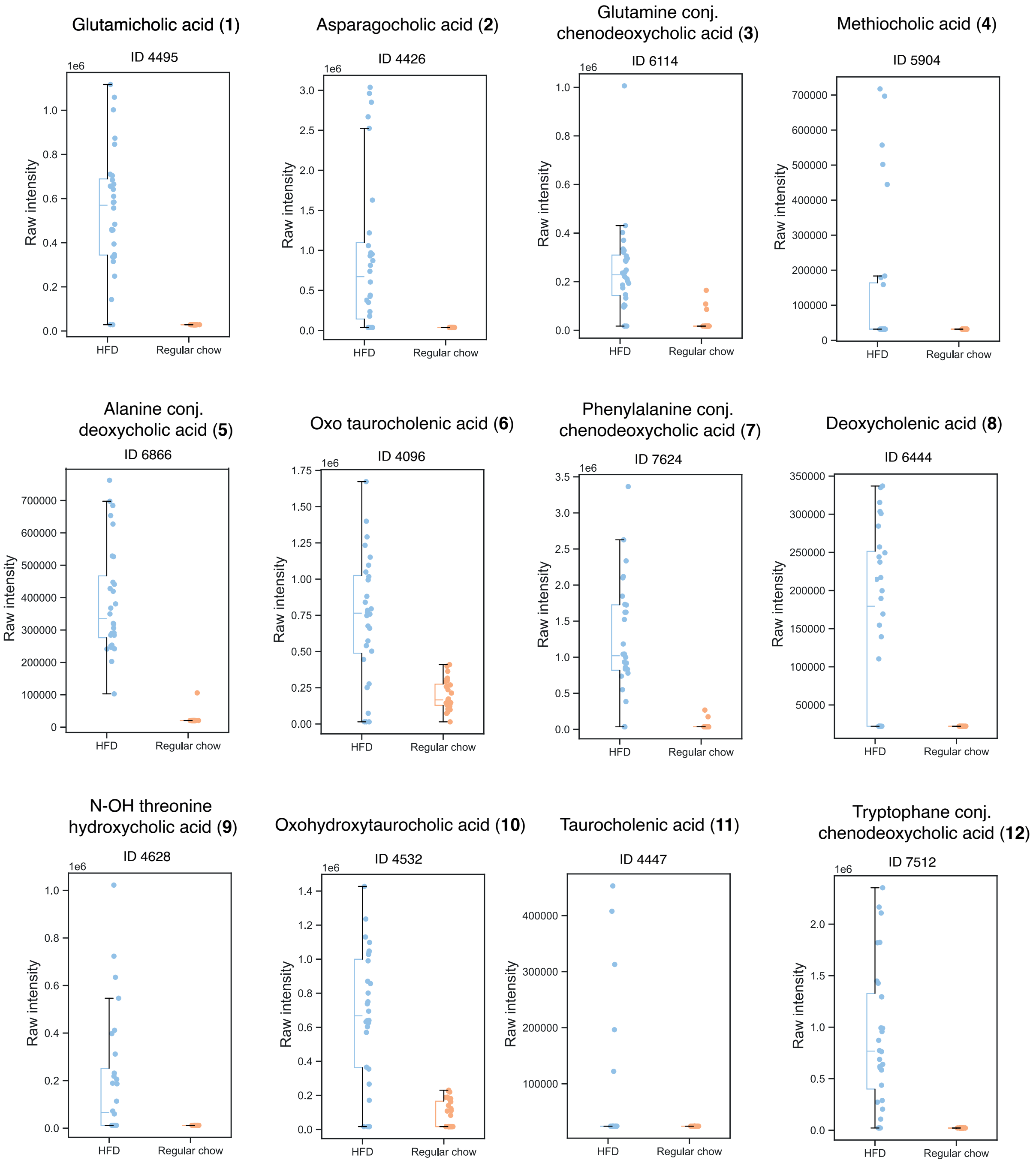

Supplementary Fig. 22: Distribution of raw intensities of novel bile acid conjugates in the mice fecal dataset. Mice were subject to a regular diet (regular chow) or under high fat diet (HFD). These bile acid conjugates **1–12** are more abundant in the HFD group. Box plots depict the first and third quartiles, as well as the median. Whiskers extend to the smallest and largest value, but no further than $1.5 \times$ IQR from the hinges, where IQR is the interquartile range. $N = 56$ independent biological experiments.

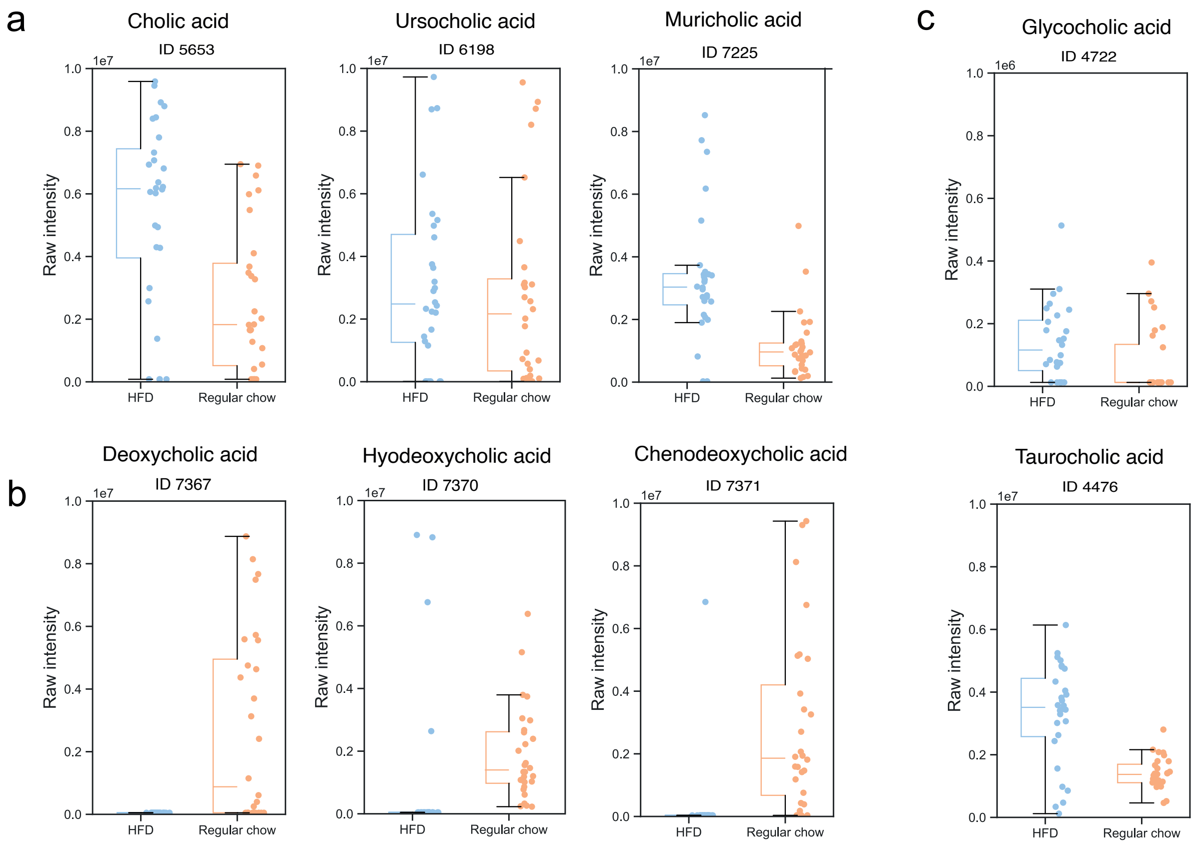

Supplementary Fig. 23: Distribution of raw intensities of standard bile acids in the mice fecal dataset. Mice were subject to a regular diet (regular chow) or under high fat diet (HFD). The abundance of primary bile acids (a) is slightly higher in the HFD group, while the secondary bile acids (b) are relatively depleted in the HFD group. The standard conjugated bile acids (c) also have a higher abundance in the HFD group. Box plots depict the first and third quartiles, as well as the median. Whiskers extend to the smallest and largest value, but no further than 1.5 × IQR from the hinges, where IQR is the interquartile range. $N = 56$ independent biological experiments.

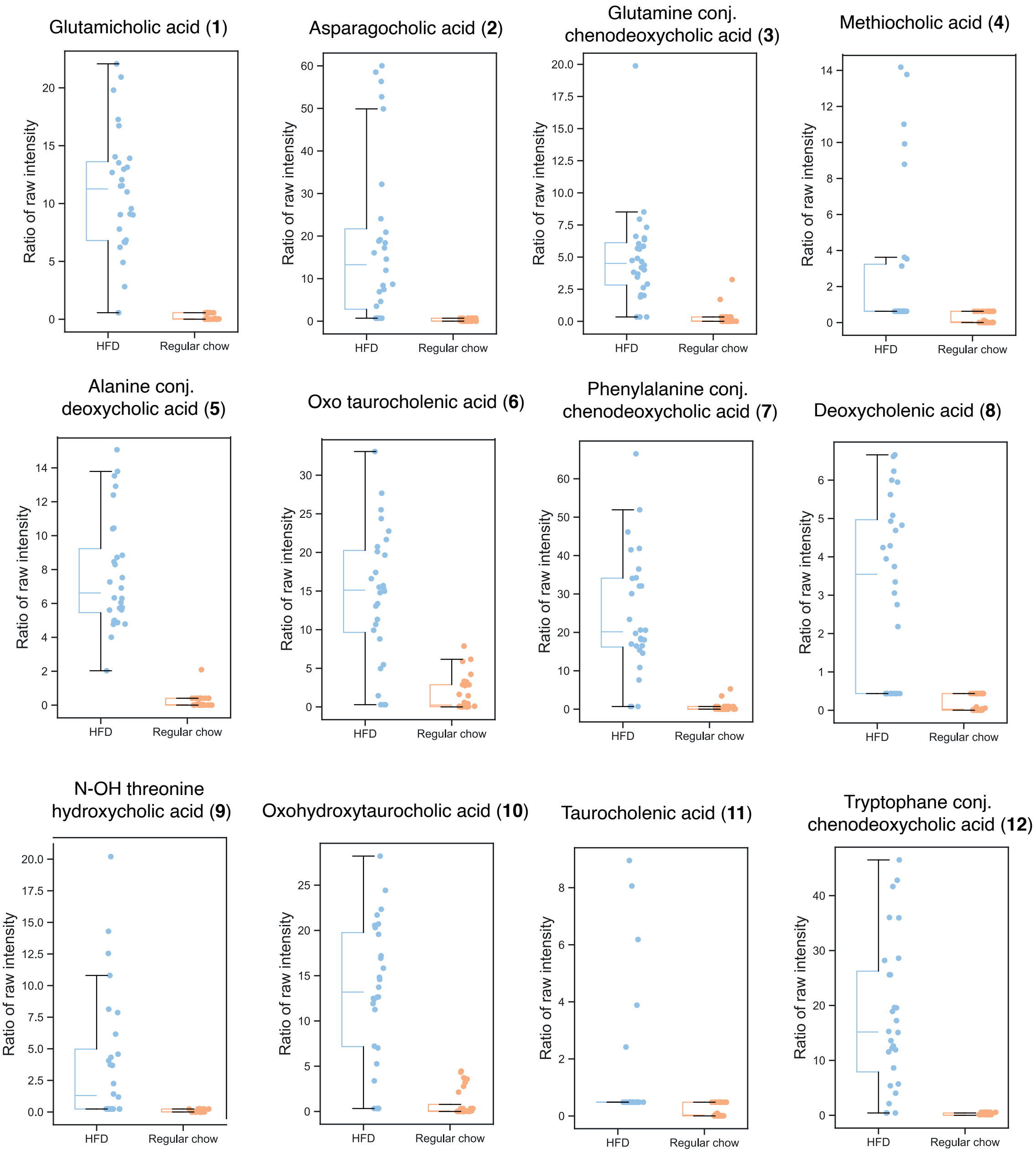

Supplementary Fig. 24: Distribution of ratios of the novel bile acid conjugates over deoxycholic acid in the mice fecal dataset. We compute the ratio between the raw intensities of the novel bile acid conjugates **1**–**12** and deoxycholic acid as a standard bile acid. Ratios are higher in the high-fat diet (HFD) group. Box plots depict the first and third quartiles, as well as the median. Whiskers extend to the smallest and largest value, but no further than $1.5 \times$ IQR from the hinges, where IQR is the interquartile range. $N = 56$ independent biological experiments.

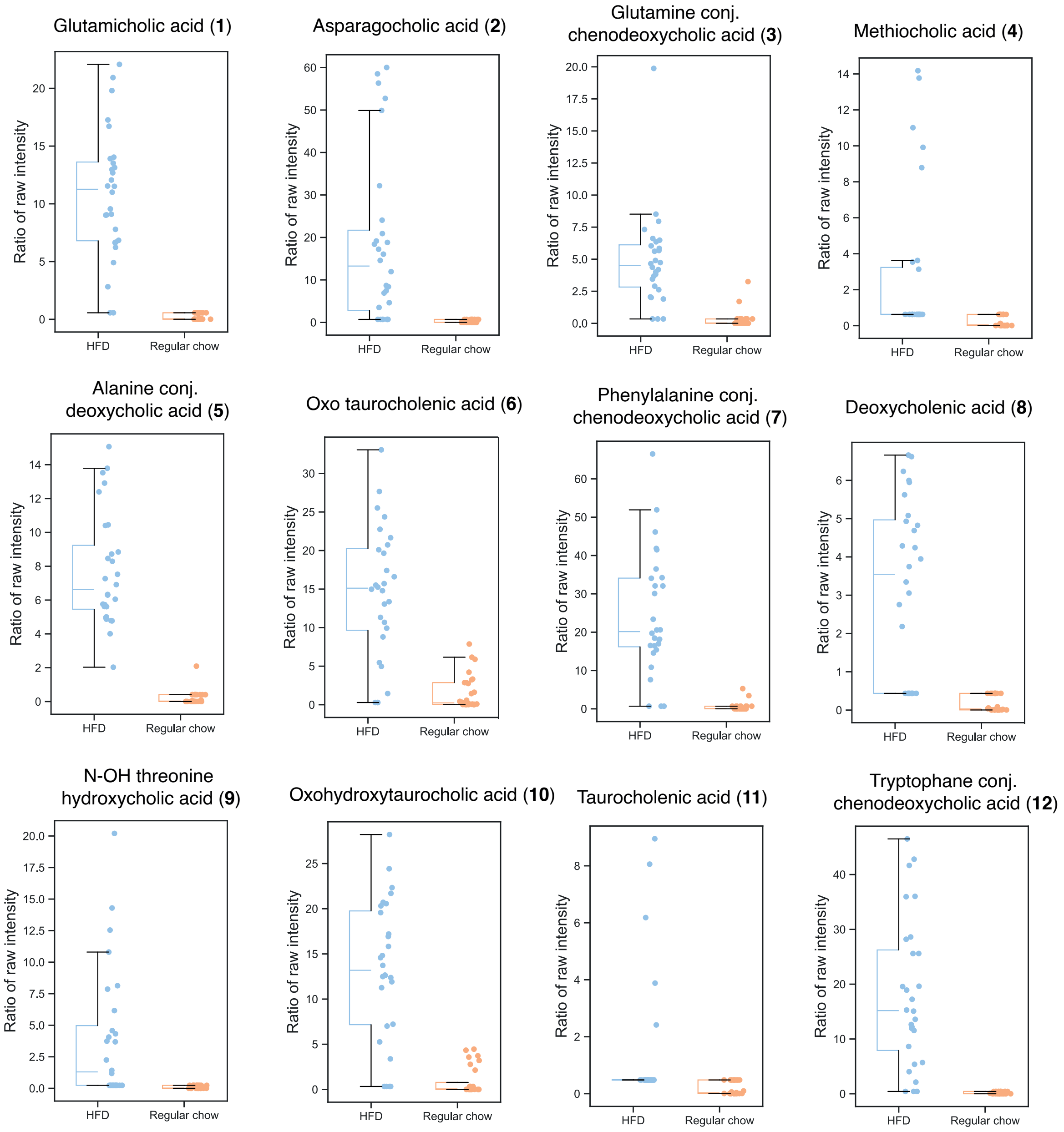

Supplementary Fig. 25: Distribution of ratios of the novel bile acid conjugates over hyodeoxycholic acid in the mice fecal dataset. We compute the ratio between the raw intensities of the novel bile acid conjugates **1**–**12** and hyodeoxycholic acid as a standard bile acid. Ratios are higher in the high-fat diet (HFD) group. Box plots depict the first and third quartiles, as well as the median. Whiskers extend to the smallest and largest value, but no further than 1.5 × IQR from the hinges, where IQR is the interquartile range. $N = 56$ independent biological experiments.

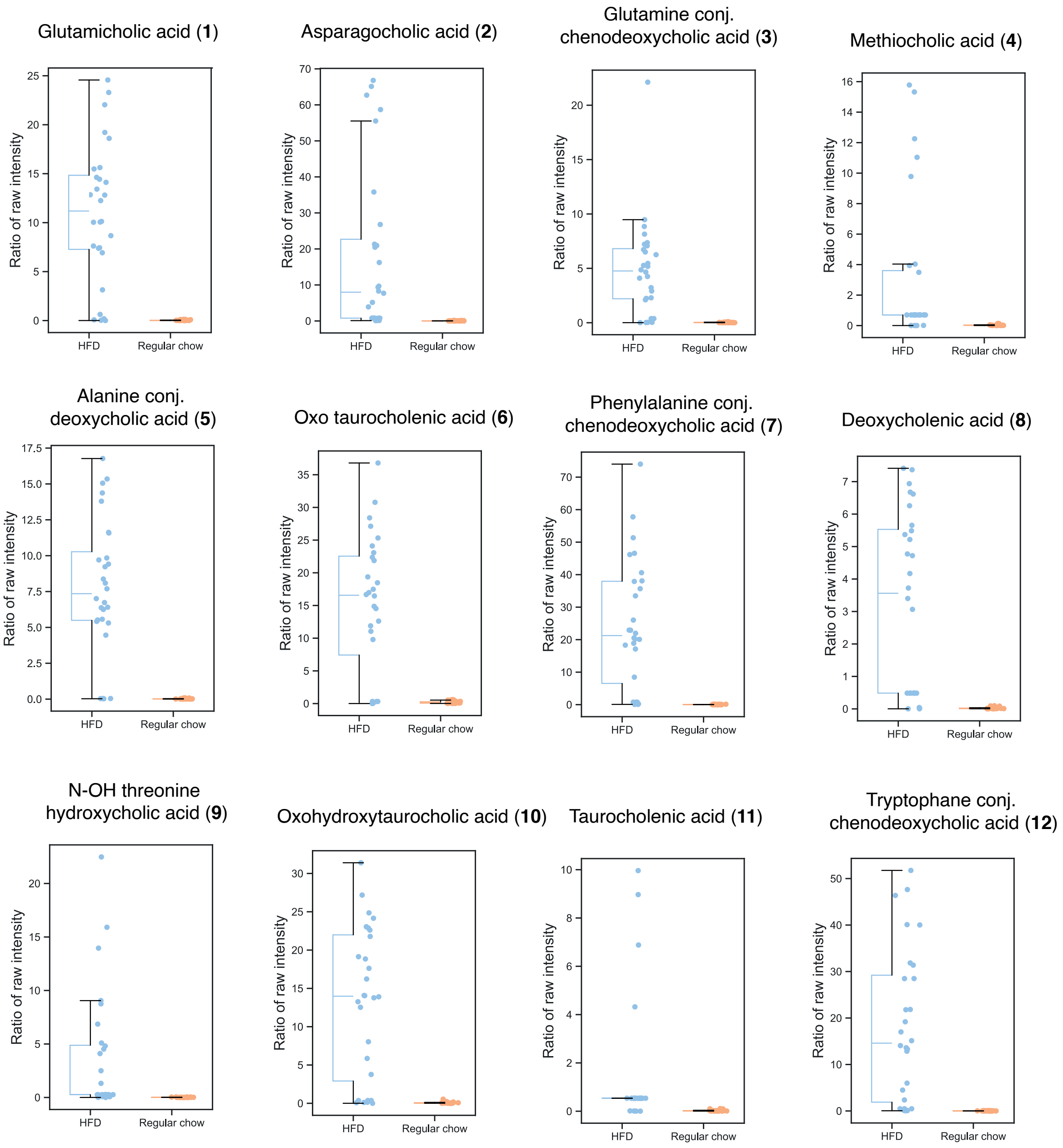

Supplementary Fig. 26: Distribution of ratios of the novel bile acid conjugates over taurocholic acid in the mice fecal dataset. We compute the ratio between the raw intensities of the novel bile acid conjugates **1–12** and taurocholic acid as a standard bile acid. Ratios are higher in the high-fat diet (HFD) group. Box plots depict the first and third quartiles, as well as the median. Whiskers extend to the smallest and largest value, but no further than 1.5 × IQR from the hinges, where IQR is the interquartile range. $N = 56$ independent biological experiments.

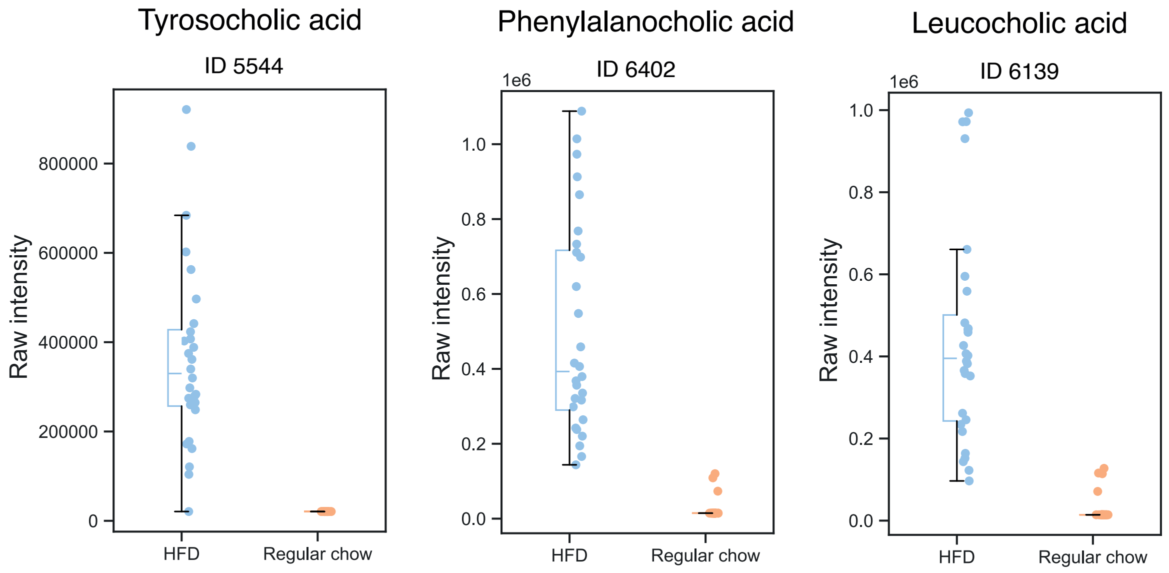

Supplementary Fig. 27: Distribution of raw intensities of three recently discovered bile acid conjugates in the mice fecal dataset. Bile acid conjugates tyrosocholic acid, phenylalanocholic acid, and leucocholic acid were first described by Quinn *et al.* (*Nature* 579, 123—129, 2020) and were also observed in the present study. Similar to the novel bile acid conjugates bac1–**12**, these bile acid conjugates are more abundant in the high-fat diet (HFD) group. Box plots depict the first and third quartiles, as well as the median. Whiskers extend to the smallest and largest value, but no further than 1.5 × IQR from the hinges, where IQR is the interquartile range. $N = 56$ independent biological experiments.

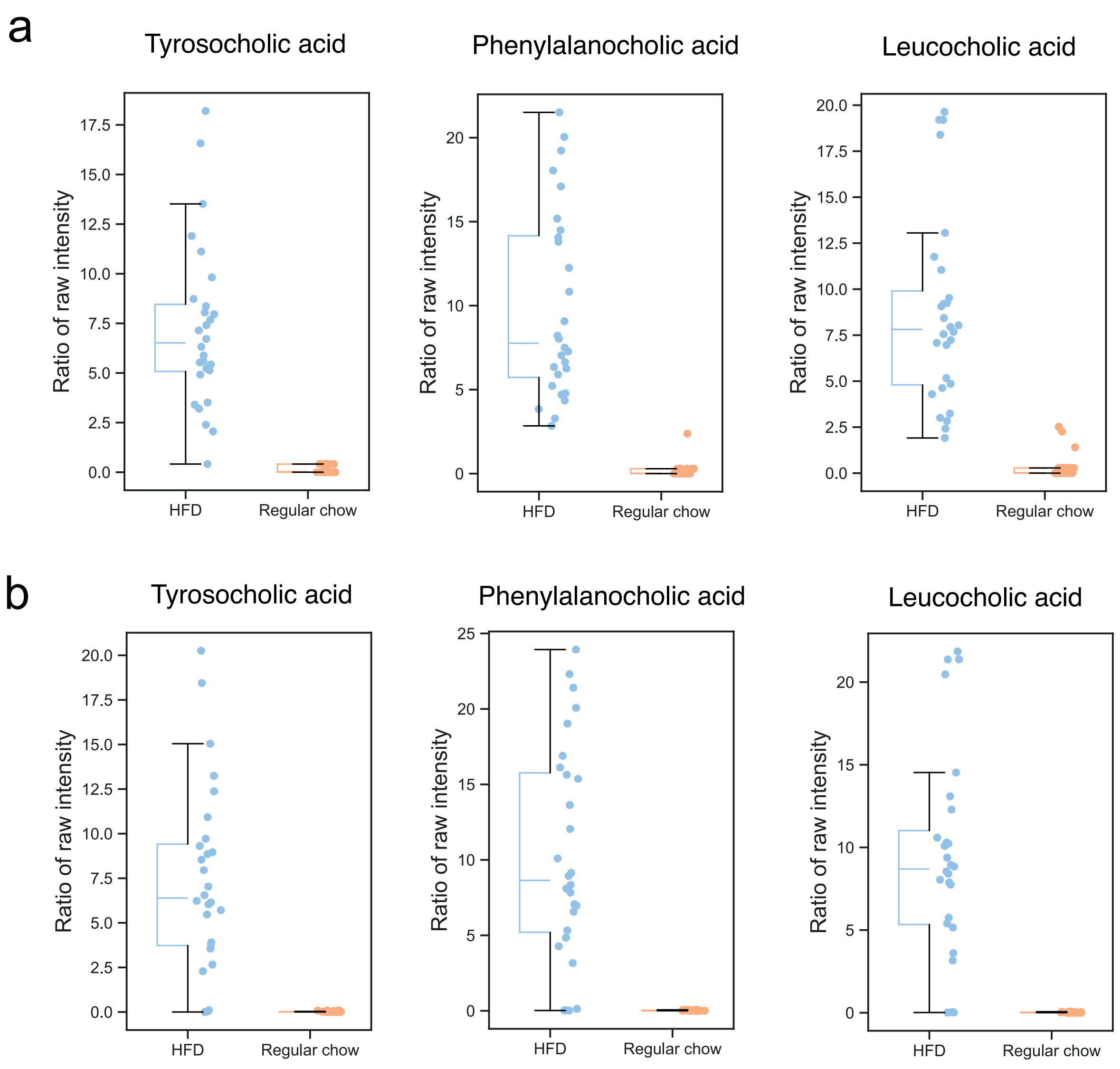

Supplementary Fig. 28: Distribution of ratios of three recently discovered bile acid conjugates over standard bile acid in the mice fecal dataset. We compute the ratio between the raw intensities of the bile acid conjugates (tyrosocholic acid, phenylalanocholic acid, leucocholic acid) from Quinn *et al.* (*Nature* 579, 123—129, 2020), and (a) deoxycholic or (b) hyodeoxycholic acid as standard bile acids. Similar to the novel bile acids conjugates **1**–**12**, ratio are higher in the high-fat diet (HFD) group. Box plots depict the first and third quartiles, as well as the median. Whiskers extend to the smallest and largest value, but no further than $1.5 \times$ IQR from the hinges, where IQR is the interquartile range. $N = 56$ independent biological experiments.

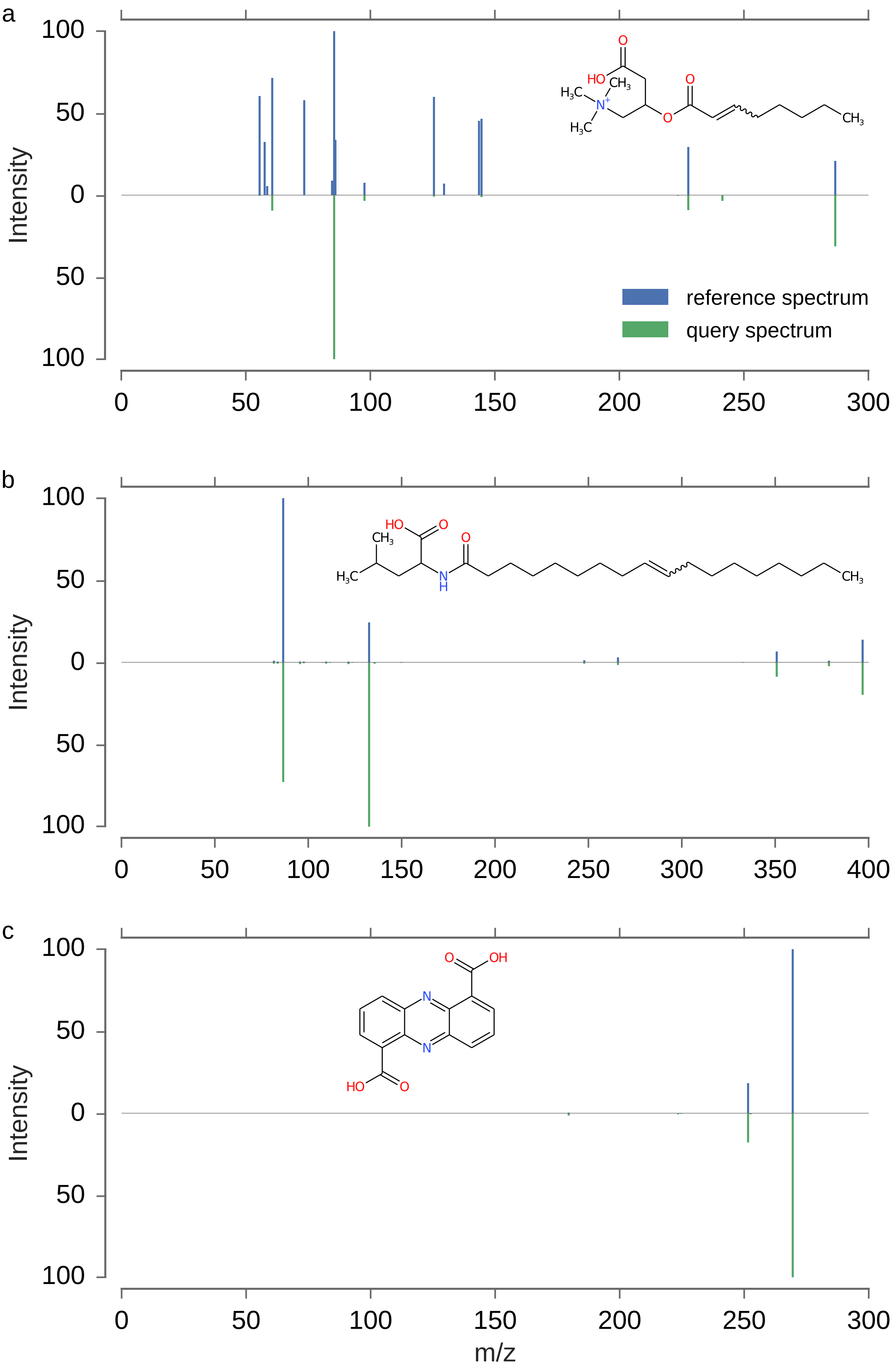

Supplementary Fig. 29: Mirror plots of fragmentation spectra for novel structure annotations in the human dataset. Reference spectra above, query spectra below the x-axis. Reference spectra of 2E-octenoyl-carnitine measured on a Sciex X500R QTOF using a collision energy ramp from 20 to 50 eV (a), N-Oleyl-Leucine measured on a Bruker maXis UHR-ToF using a collision energy of 10 eV (b) and phenazine-1,6-dicarboxylic acid measured on a Bruker maXis UHR-ToF using a collision energy of 10 eV (c). All query spectra were measured on Q Exactive Orbitrap instruments with 30 eV (a) and 21 eV (b,c), respectively. Recall that “novel structure” refers to the fact that no training data for CSI:FingerID or COSMIC were available for this structure.

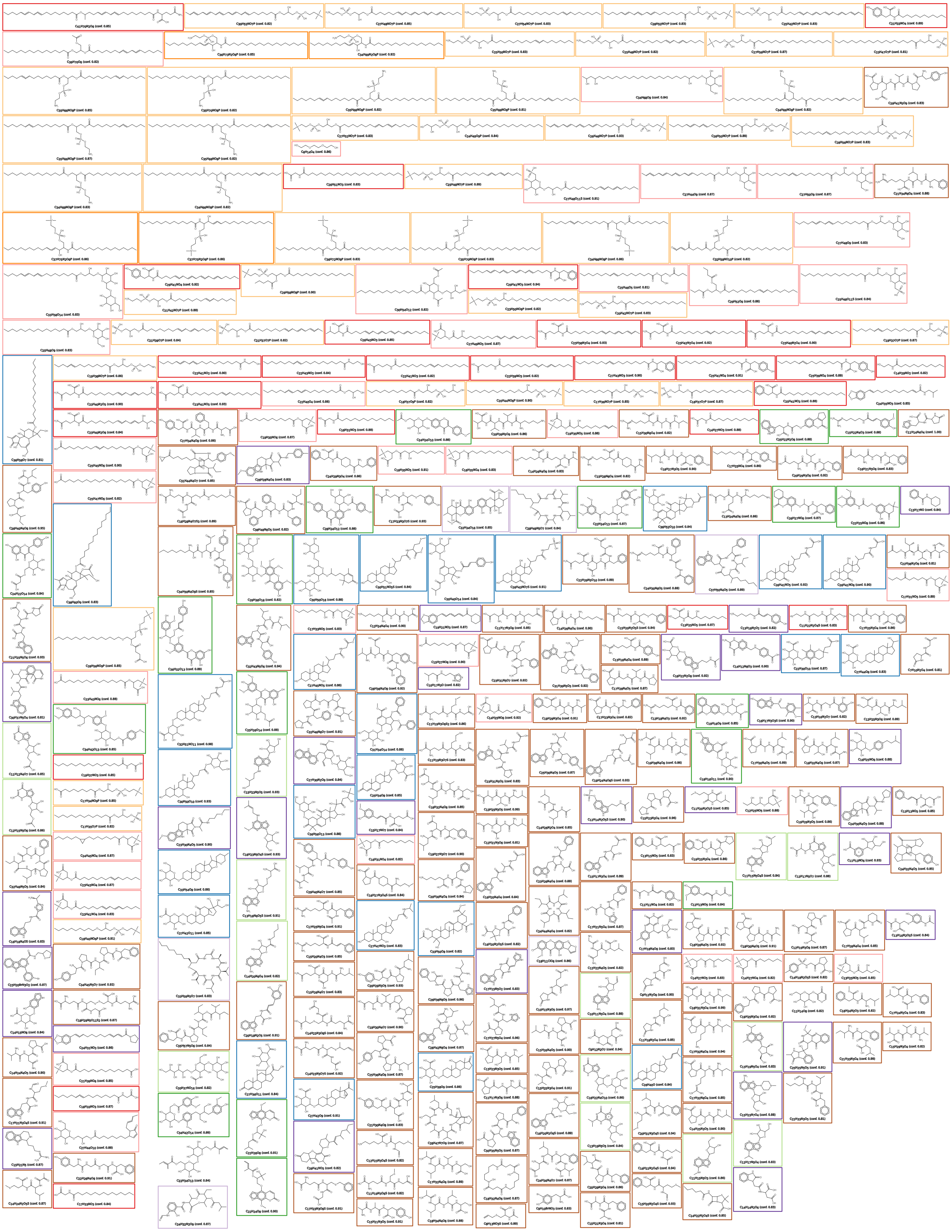

Supplementary Fig. 30: The 1,715 novel molecular structures annotated with high confidence in the Orbitrap dataset. Confidence score threshold 0.64 was used. Structures are shown with identification number (ID), molecular formula and COSMIC confidence score. Colors indicate compound classes. Lipid structures must again be interpreted with some care.

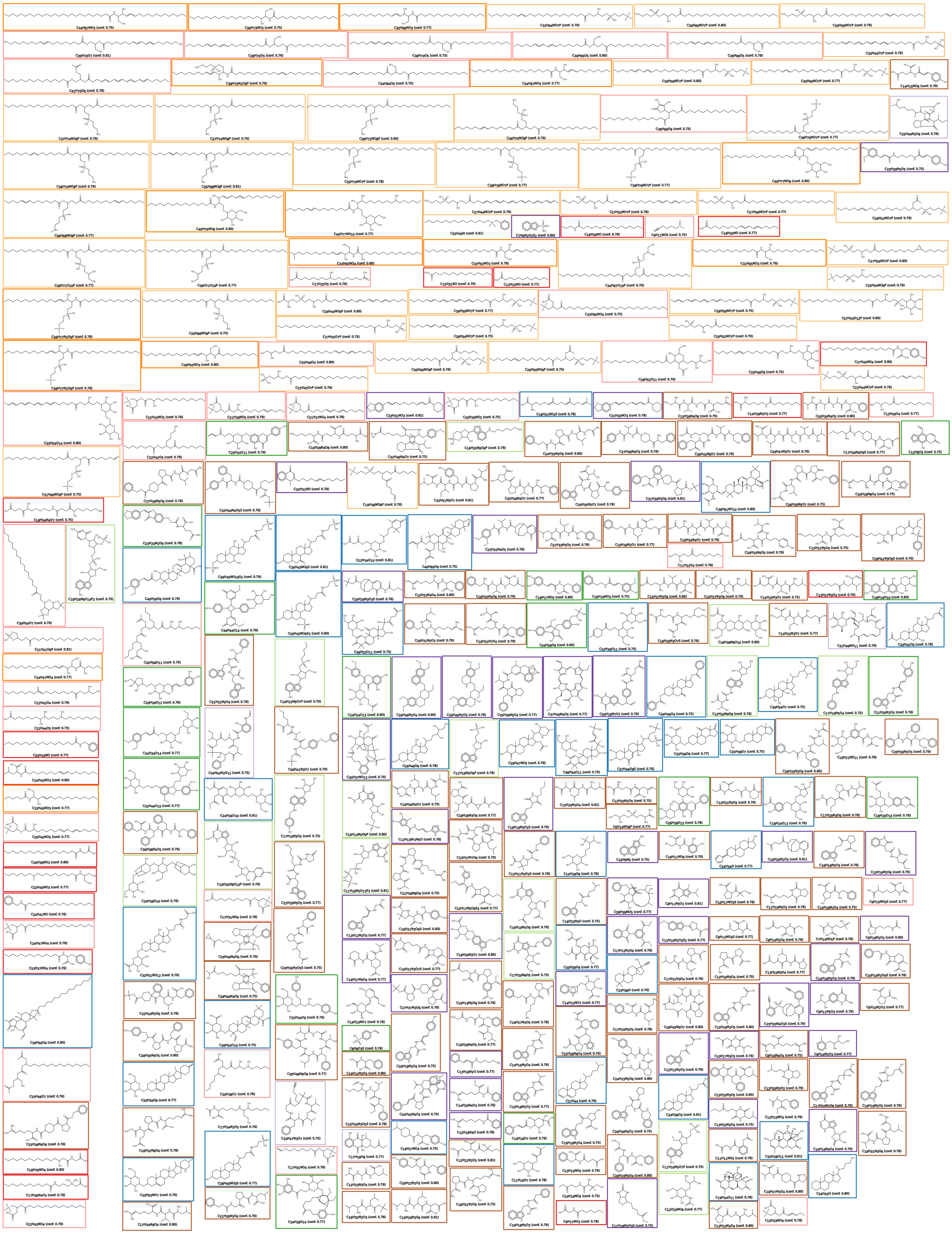

Supplementary Fig. 30: The 1,715 novel molecular structures annotated with high confidence in the Orbitrap dataset (cont.).

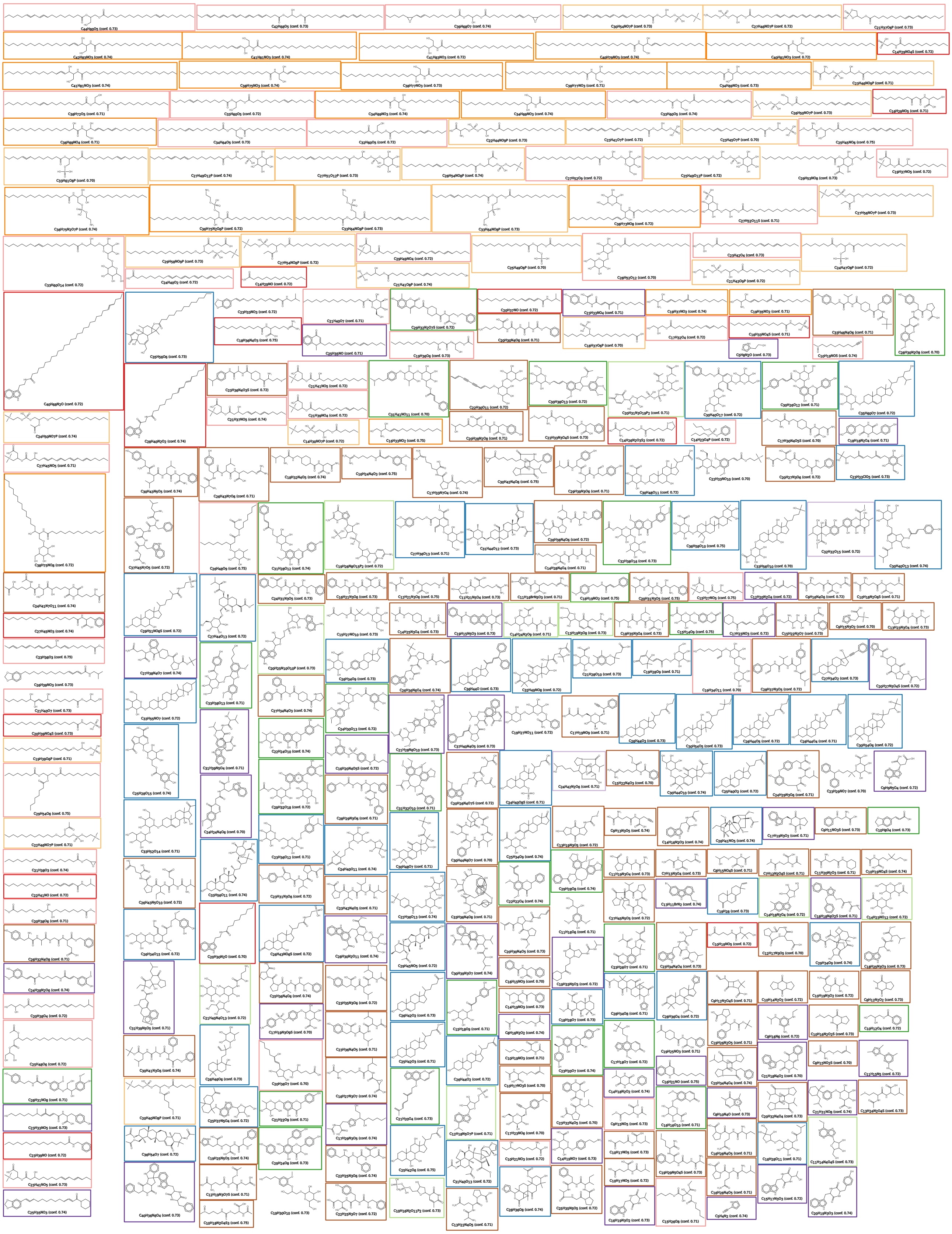

Supplementary Fig. 30: The 1,715 novel molecular structures annotated with high confidence in the Orbitrap dataset (cont.).

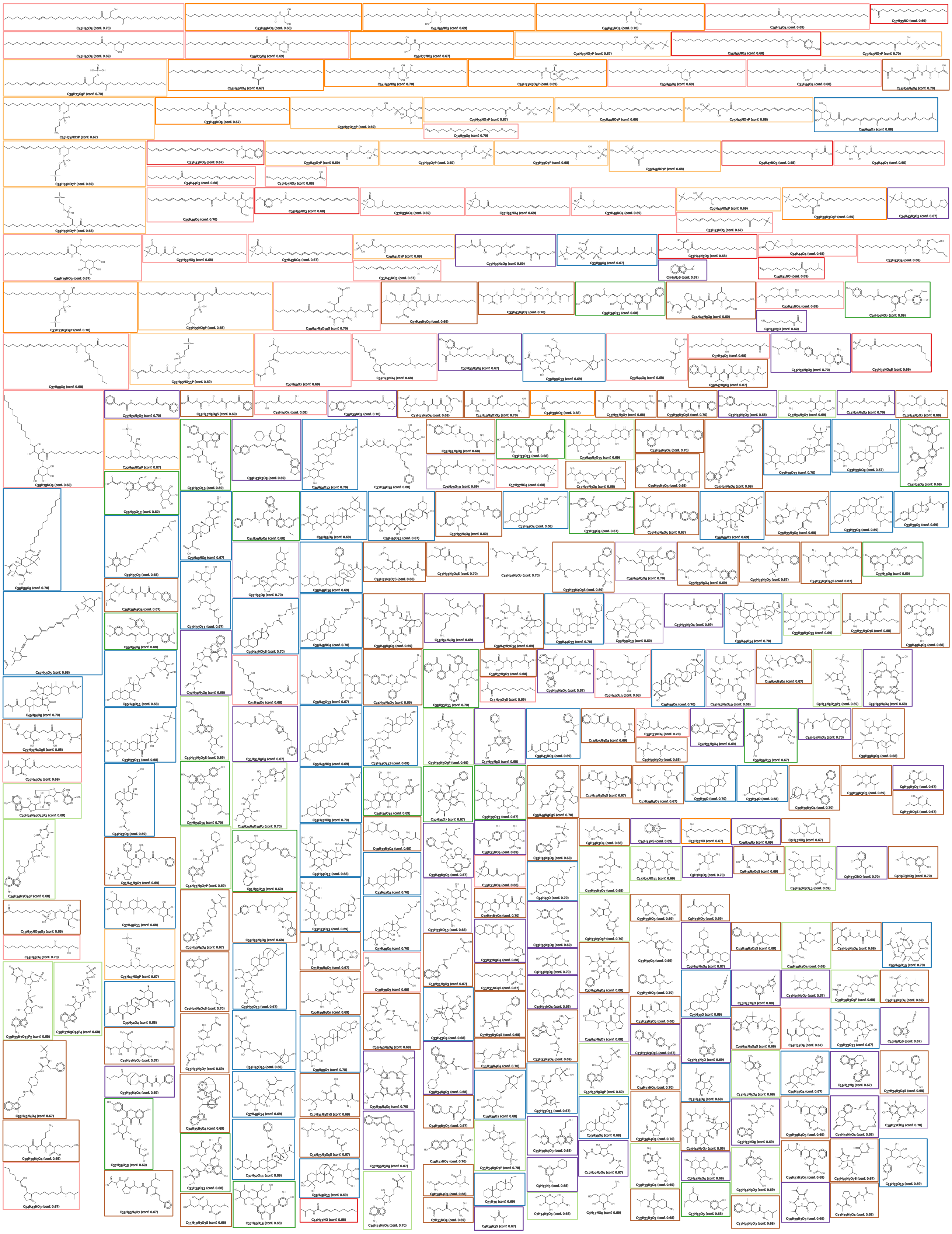

Supplementary Fig. 30: The 1,715 novel molecular structures annotated with high confidence in the Orbitrap dataset (cont.).

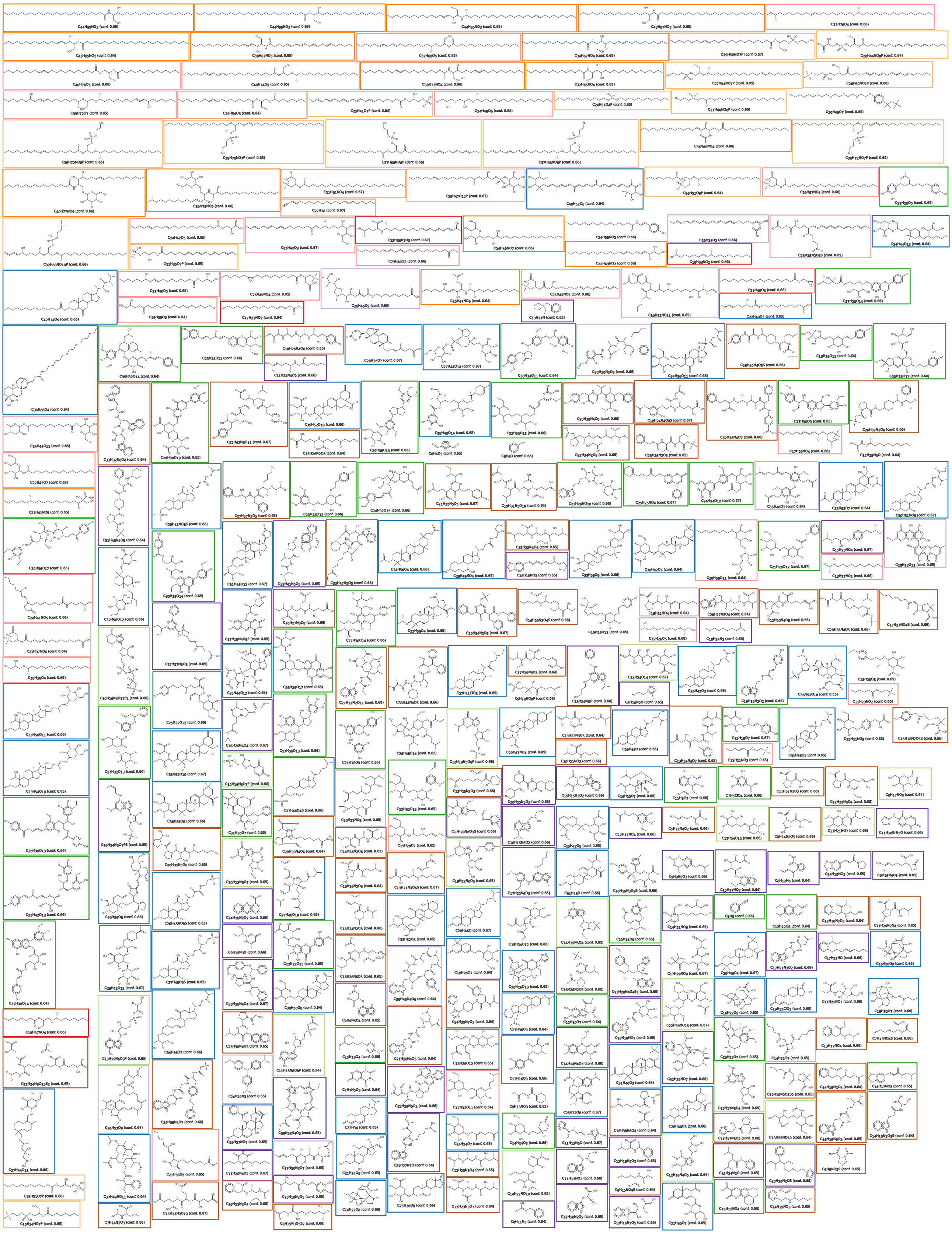

Supplementary Fig. 30: The 1,715 novel molecular structures annotated with high confidence in the Orbitrap dataset (cont.).
