# Supplementary material for: High-confidence structural annotation of metabolites absent from spectral libraries
Source: Nat Biotechnol. 2021 Oct 14;40(3):411–21. doi: 10.1038/s41587-021-01045-9 (PMC8926923; doi:10.1038/s41587-021-01045-9)
Supplement: Supplementary file 2 — Reporting Summary [file 41587_2021_1045_MOESM2_ESM.pdf]

nature research

Corresponding author(s): Sebastian Böcker

Last updated by author(s): Jul 29, 2021

Reporting Summary

Nature Research wishes to improve the reproducibility of the work that we publish. This form provides structure for consistency and transparency in reporting. For further information on Nature Research policies, see our Editorial Policies and the Editorial Policy Checklist.

Statistics

For all statistical analyses, confirm that the following items are present in the figure legend, table legend, main text, or Methods section.

n/a	Confirmed	
☐	☒	The exact sample size (*n*) for each experimental group/condition, given as a discrete number and unit of measurement
☒	☐	A statement on whether measurements were taken from distinct samples or whether the same sample was measured repeatedly
☒	☐	The statistical test(s) used AND whether they are one- or two-sided<br>*Only common tests should be described solely by name; describe more complex techniques in the Methods section.*
☒	☐	A description of all covariates tested
☒	☐	A description of any assumptions or corrections, such as tests of normality and adjustment for multiple comparisons
☐	☒	A full description of the statistical parameters including central tendency (e.g. means) or other basic estimates (e.g. regression coefficient) AND variation (e.g. standard deviation) or associated estimates of uncertainty (e.g. confidence intervals)
☒	☐	For null hypothesis testing, the test statistic (e.g. *F*, *t*, *r*) with confidence intervals, effect sizes, degrees of freedom and *P* value noted<br>*Give P values as exact values whenever suitable.*
☒	☐	For Bayesian analysis, information on the choice of priors and Markov chain Monte Carlo settings
☒	☐	For hierarchical and complex designs, identification of the appropriate level for tests and full reporting of outcomes
☒	☐	Estimates of effect sizes (e.g. Cohen's *d*, Pearson's *r*), indicating how they were calculated

Our web collection on statistics for biologists contains articles on many of the points above.

Software and code

Policy information about availability of computer code

Data collection	Sciex 2.0 software was used measuring and preprocessing the Sciex dataset
Data analysis	Beyond the software which is part of this paper, GNPS (https://gnps.ucsd.edu/, version number recorded individually) was used to compute molecular networks and Cytoscape 3.7.1 (https://cytoscape.org/) was used to visualize molecular networks. SIRIUS is available from https://github.com/boecker-lab/sirius/.

For manuscripts utilizing custom algorithms or software that are central to the research but not yet described in published literature, software must be made available to editors and reviewers. We strongly encourage code deposition in a community repository (e.g. GitHub). See the Nature Research guidelines for submitting code & software for further information.

Data

Policy information about availability of data

All manuscripts must include a data availability statement. This statement should provide the following information, where applicable:

- Accession codes, unique identifiers, or web links for publicly available datasets
- A list of figures that have associated raw data
- A description of any restrictions on data availability

The Sciex dataset has been deposited on GNPS, accession numbers CCMSLIB00006581625 to CCMSLIB00006581938. Input mzML/mzXML files are available at MassIVE (https://massive.ucsd.edu/) with accession nos. MSV000082973 (mice fecal dataset); MSV000084630 (mass spectrometry analysis of the synthetic standards for Phe-CDCA and Trp-CDCA); MSV000083559, MSV000079651, MSV000080167, MSV000080469, MSV000080533, MSV000080627, MSV000081351, MSV000082261, MSV000082629, MSV000082630 (human dataset). See Supplementary Table 4 for accession numbers of the Orbitrap dataset. Metadata for synthetic standards of Phe-CDCA and Trp-CDCA were deposited with the dataset (id no. MSV000084630). Fragmentation spectra of Phe-CDCA and Trp-CDCA were deposited on GNPS (CCMSLIB00005467952 and CCMSLIB00005716808). Fragmentation spectra of all other manually confirmed bile acid conjugates were also

deposited on GNPS; see Supplementary Table 2 for individual spectra ids. Fragmentation spectra of N-oleyl-leucine (RP029701 ([M+H]+, 10 eV), RP029702 ([M+H]+, 20 eV), RP029703([M+H]+, 40 eV)) and phenazine-1,6-dicarboxylic acid (RP018701 ([M+H]+, 10 eV), RP018702 ([M+H]+, 20 eV), RP018703 ([M+H]+, 40 eV)) are available from MassBank. The fragmentation spectrum of (2E)-octenoyl-carnitine was deposited on GNPS (CCMSLIB00006581932). Parameters and results of LC-MS/MS processing for the mice fecal dataset are available at https://gnps.ucsd.edu/ProteoSAFe/status.jsp?task=e78a8c8f429a46fcb24f3b34d69aff25. The bile acid conjugate structure database is available at https://github.com/lfnothias/Combinatorial_BileAcids_DB_COSMIC. Spectral libraries generated from the high-confidence COSMIC annotations of the mice fecal, human and Orbitrap datasets are available from https://bio.informatik.uni-jena.de/cosmic/. The biomolecule structure database is a union of the following structure databases: HMDB (http://www.hmdb.ca), KNApSAcK (http://kanaya.naist.jp/knapsack), CHEBI (https://www.ebi.ac.uk/chebi/), KEGG (http://www.kegg.jp/), HSDB (https://toxnet.nlm.nih.gov/cgi-bin/sis/htmlgen?HSDB), MACONDA (http://www.maconda.bham.ac.uk), BIOCYC (http://biocyc.org/), structures from GNPS (https://gnps.ucsd.edu), biological subset of ZINC (http://zinc.docking.org), structures from MassBank (http://www.massbank.jp), UNDP (http://pkuxxj.pku.edu.cn/UNPD), PLANTCYC (http://pmn.plantcyc.org/), YMDB (http://www.ymdb.ca/compounds/YMDB). SMILES of all structures in the biomolecule structure database can be downloaded from https://bio.informatik.uni-jena.de/cosmic/. The PubChem structure database is available for download from https://ftp.ncbi.nlm.nih.gov/pubchem/Compound/ (Jan 16, 2019).

Field-specific reporting

Please select the one below that is the best fit for your research. If you are not sure, read the appropriate sections before making your selection.

☒ Life sciences ☐ Behavioural & social sciences ☐ Ecological, evolutionary & environmental sciences

For a reference copy of the document with all sections, see nature.com/documents/nr-reporting-summary-flat.pdf

Life sciences study design

All studies must disclose on these points even when the disclosure is negative.

Sample size	For the bile acids fold change analysis, we analyzed N = 56 independent biological experiments.
Data exclusions	For the bile acids fold change analysis, only data from the high-fat diet and standard diet groups were used.
Replication	For the bile acids study, a single LC-MS experiment was performed per sample.
Randomization	For the bile acids study, the sequence of LC-MS sample acquisition was randomised.
Blinding	Does not apply to the study.

Reporting for specific materials, systems and methods

We require information from authors about some types of materials, experimental systems and methods used in many studies. Here, indicate whether each material, system or method listed is relevant to your study. If you are not sure if a list item applies to your research, read the appropriate section before selecting a response.

Materials & experimental systems

n/a	Involved in the study
☒	☐ Antibodies
☒	☐ Eukaryotic cell lines
☒	☐ Palaeontology and archaeology
☒	☐ Animals and other organisms
☒	☐ Human research participants
☒	☐ Clinical data
☒	☐ Dual use research of concern

Methods

n/a	Involved in the study
☒	☐ ChIP-seq
☒	☐ Flow cytometry
☒	☐ MRI-based neuroimaging
